# Deconvoluting Nonlinear Catalyst–Substrate Effects in the Intramolecular Dirhodium-Catalyzed C–H Insertion of Donor/Donor Carbenes Using Data Science Tools

**DOI:** 10.1021/acscatal.3c04256

**Published:** 2023-12-11

**Authors:** Lucas W. Souza, Beck R. Miller, Ryan C. Cammarota, Anna Lo, Ixchel Lopez, Yuan-Shin Shiue, Benjamin D. Bergstrom, Sarah N. Dishman, James C. Fettinger, Matthew S. Sigman, Jared T. Shaw

**Affiliations:** † Department of Chemistry, University of Utah, Salt Lake City, Utah 84112, United States; ‡ Department of Chemistry, University of California, Davis, California 95616, United States

**Keywords:** dirhodium, C–H insertion, data science, nonlinear, catalyst-substrate interactions

## Abstract

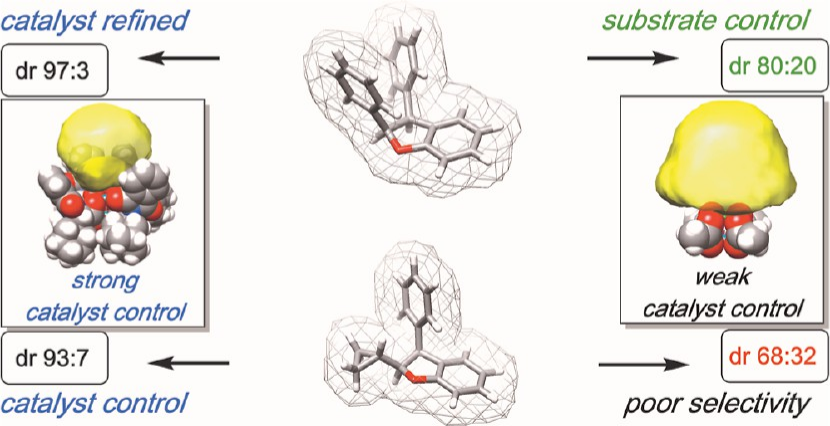

Interactions between catalysts and substrates can be highly complex and dynamic, often complicating the development of models to either predict or understand such processes. A dirhodium(II)-catalyzed C–H insertion of donor/donor carbenes into 2-alkoxybenzophenone substrates to form benzodihydrofurans was selected as a model system to explore nonlinear methods to achieve a mechanistic understanding. We found that the application of traditional methods of multivariate linear regression (MLR) correlating DFT-derived descriptors of catalysts and substrates leads to poorly performing models. This inspired the introduction of nonlinear descriptor relationships into modeling by applying the sure independence screening and sparsifying operator (SISSO) algorithm. Based on SISSO-generated descriptors, a high-performing MLR model was identified that predicts external validation points well. Mechanistic interpretation was aided by the deconstruction of feature relationships using chemical space maps, decision trees, and linear descriptors. Substrates were found to have a strong dependence on steric effects for determining their innate cyclization selectivity preferences. Catalyst reactive site features can then be matched to product features to tune or override the resultant diastereoselectivity within the substrate-dictated ranges. This case study presents a method for understanding complex interactions often encountered in catalysis by using nonlinear modeling methods and linear deconvolution by pattern recognition.

## Introduction

**Figure 1:**
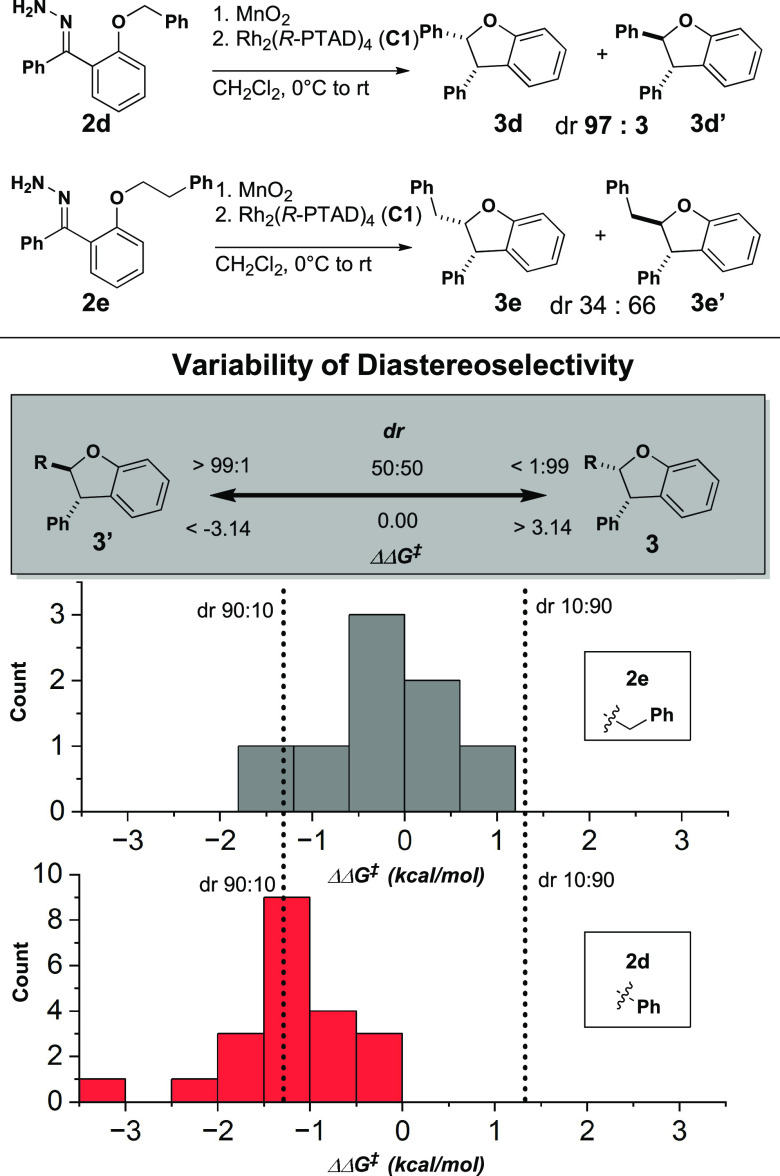
(Top) Previous work with donor/donor carbenes highlighting changes in diastereoselectivity. (Bottom) Histograms of diastereoselectivity observed for select substrates.

When optimizing reaction conditions for stereoselectivity, experimental effort is often focused on a single model substrate.^1,2^ This is practical because evaluating many substrates can require significant time/resources for reaction development.^3^ While this approach often concludes with achieving the desired stereoselectivity for a single substrate, these conditions do not necessarily translate to new substrates, thereby requiring additional rounds of catalyst evaluation. As an example, in a previous study by one of our teams, a dirhodium(II)-catalyzed C–H insertion of donor/donor carbenes into 2-alkoxybenzophenones to form benzodihydrofurans was optimized to proceed with high diastereoselectivity for a select number of substrates.^4^ However, seemingly minor structural changes to the substrate were observed to yield disparate diastereomeric outcomes under the same conditions (Figure 1).

Data science tools have been developed to address this common issue by exploring how catalyst and substrate structural features impact reaction outcomes.^5−8^ Specifically, the correlation of molecular features to selectivity using multivariate linear regression (MLR) has provided a tool to connect mathematical relationships to fundamental structural properties related to the origins of selectivity.^9−11^ These methods have been used to successfully elucidate the origins of selectivity between catalysts and substrates, both individually and combinatorially.^12−15^

However, automated efforts to co-optimize multiple reaction components often exhibit limited accuracy in predicting out-of-sample data points due to the complexity of these interactions.^16,17^ Variation in multiple structural features influencing the physical interaction between reaction components is challenging to capture without directly computing the combined structure. The resources required for such computations increase substantially with combinatorial pairings of multiple catalysts and substrates across ensembles of catalytically relevant conformations. Thus, a method for indirectly analyzing interaction effects from the properties of individual components is highly sought after.

In this context, we selected the intramolecular dirhodium(II)-catalyzed C–H insertion of donor/donor carbenes into 2-alkoxybenzophenones as a case study. This is due to the wide variability in observed diastereoselectivity dependent on catalyst–substrate interactions (Figure 1). Many structurally diverse dirhodium(II) paddlewheel complexes are commercially available for this reaction type, and accessing variation in the substrate is straightforward, allowing for rapid modulation of structural features for both reaction components.^18^ We initially evaluated MLR statistical modeling techniques but found this approach did not sufficiently capture the complex relationship between catalyst and substrate features. Thus, we applied nonlinear feature transformations to effectively build statistical models that can predict the diastereoselectivity for new catalyst–substrate pairings. However, nonlinear models are notoriously difficult to interpret in a chemically meaningful way. Analyzing subsets of the data partitioned by trends in chemical space allowed for the deconvolution of these nonlinear parameters, yielding a set of general guidelines for optimizing diastereoselectivity through the careful feature pairing of both reaction components.

Herein, we present a data science-enabled analysis of the diastereoselectivity for an intramolecular dirhodium(II)-catalyzed C–H insertion of donor/donor carbenes. A diverse experimental matrix informed by data science was employed to efficiently sample catalyst–substrate interaction effects. Analysis of the resultant descriptor relationships identified by statistical models provides a platform for global structural analysis to achieve optimal diastereoselectivity for diverse catalyst–substrate pairings. Furthermore, the applicability of nonlinear modeling methods to complex systems is demonstrated through the analysis of the interplay between catalyst and substrate interactions without requiring extensive calculations.

## Results and Discussion

### Generation of a Diverse Experimental Matrix

**Figure 2:**
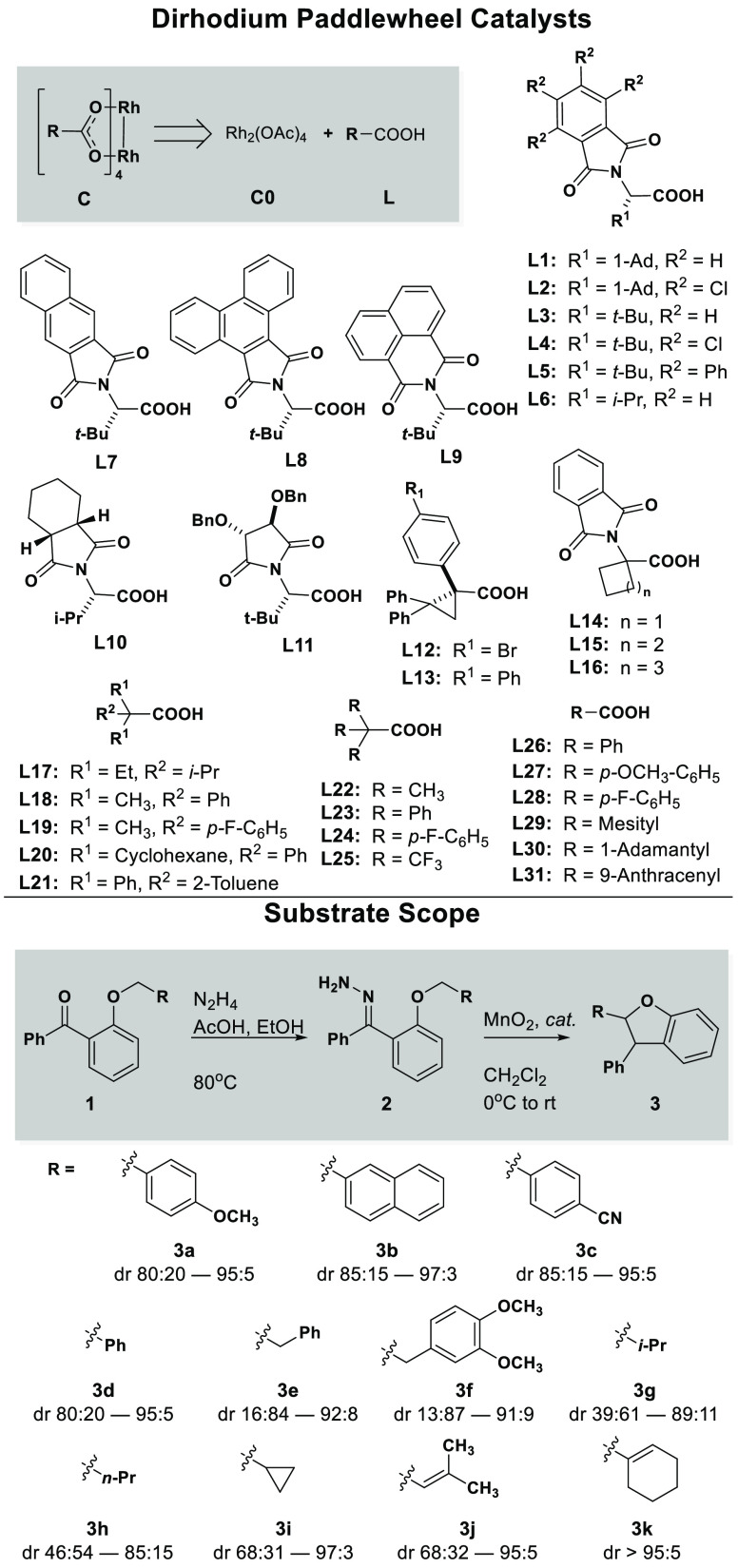
Catalyst and substrate scope. Chiral and achiral carboxylate ligands were selected by literature precedent. Observed dr ranges vary greatly, depending upon substrate identity.

**Figure 3:**
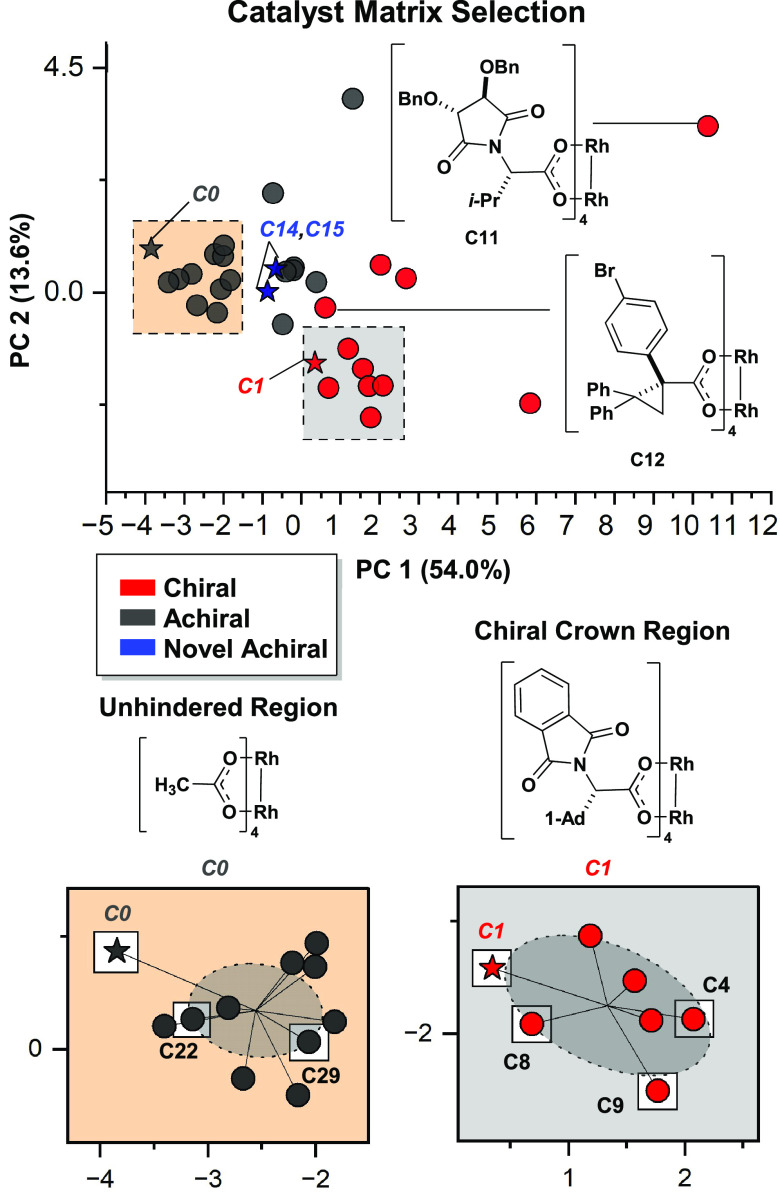
Selection of a diverse set of catalysts by PCA enhanced feature representation for MLR modeling and external model validation. A combined screen of global and local catalyst selection allowed for efficient sampling of the feature space.

A representative reaction matrix was employed to efficiently sample structures of interest and to provide better statistical sampling of reaction outputs for downstream analysis (Figure 2). For catalyst selection, electronic and steric descriptors were calculated from a single conformer computed by DFT based on the X-ray crystal structure coordinates of the full catalysts (see Supporting Information). Using these descriptors, a chemical space representation was constructed using principal component analysis (PCA) to highlight feature diversity across the available catalysts (Figure 3).

Two distinct regions of chemical space were identified by the similarity of catalyst features. Chiral structures near Rh_2_ (*S*-PTAD)_4_ (**C1**) lie in the chiral crown catalyst region, as they all generate distinct C_4_-symmetric crown conformations in solution.^19−21^ Achiral structures near Rh_2_ (OAc)_4_ (**C0**) lie in the unhindered region. These catalysts are not encumbered by bulky ligands and feature more accessible axial pockets with a larger void space. Catalysts were selected from within these regions of chemical space based on their relative proximity to **C0** and **C1**. Additionally, catalysts **C10–12** and **C17** were selected from across the chemical space to sample diverse catalyst features.

**Scheme 1:**
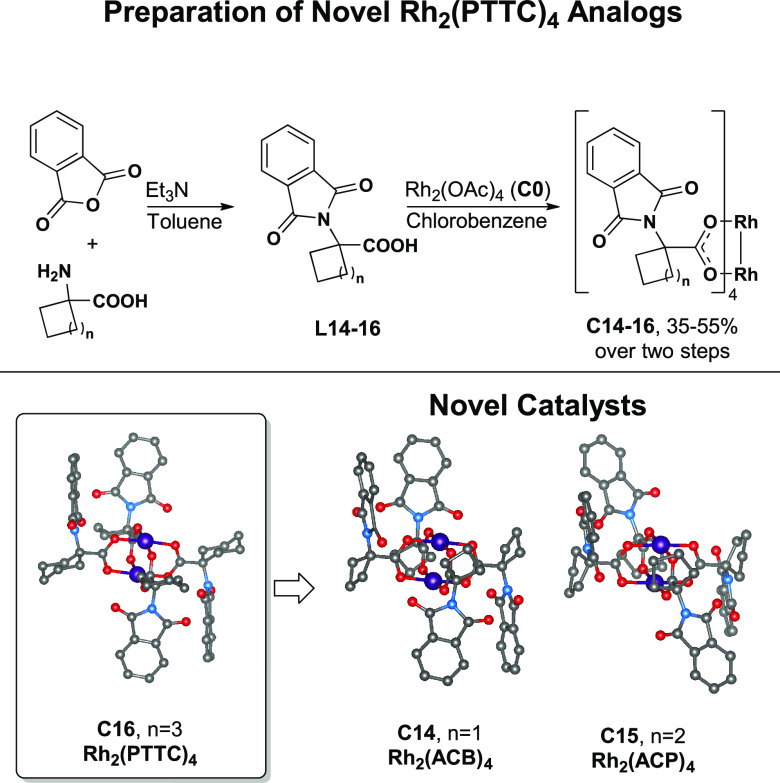
Synthesis and Single-Crystal X-ray Structures of Rh_2_ (PTTC)_4_ and Novel Achiral Catalysts

Two novel catalysts, **C14** and **C15**, were also synthesized (Scheme 1). Inspired by **C16**, the additions of **C14** and **C15** expanded the feature diversity of achiral catalysts that mimic the chiral crown environment. The structures of these catalysts were obtained by single-crystal X-ray diffraction. These catalysts fall between the **C0** and **C1** regions of chemical space, indicating they possess a unique combination of properties. In addition to the 18 catalysts selected for the training set, catalysts **C14–C16** were withheld for the external validation of the final statistical models.

A similar approach was applied to substrate selection. Previously evaluated substrates included a limited array of benzyl, alkyl, allyl, and propargyl ethers. Additional substrates were selected to enhance the electronic and steric feature diversity within these categories (Figure S13). Propargyl ethers readily form the intramolecular dipolar cycloaddition product in significant quantities^4^ and thus were excluded from the modeling set. Of the 10 substrates selected, two were reserved for external validation of the final statistical models (see the Supporting Information). Finally, the representative reaction components were paired in a combinatorial matrix for data collection.

### Parameterization of Representative Catalysts and Substrates

Current understanding of the impact of catalyst symmetry on degrees of freedom supports that conformers with the highest symmetry are traditionally regarded as imparting the highest selectivity.^22−25^ Consequently, transition state studies for these systems are commonly performed on C_4_ chiral crown conformations derived from the X-ray crystal structures of chiral catalysts.^4,26^ However, conformations with lower symmetry states or disrupted chiral crowns have been shown to outperform their C_4_ counterparts for select systems, indicating that a more holistic approach to catalyst parameterization must be considered.^19,27,28^ Catalysts were considered as conformational ensembles for parameterization, and an automated analysis of Rh pocket symmetry was employed (see Supporting Information).

Many spatial descriptors have been developed^29−31^ and successfully applied in the quantifying reactive features of mononuclear catalysts.^32,33^ Dirhodium catalysts pose a distinct challenge for these traditional descriptors with two separate axial pockets that can be symmetric or exhibit differential reactivity.^34−36^ We recently reported the development of spatial modeling for approachable rigid targets (SMART) as a specialized set of molecular descriptors designed for reactive site cavities.^37^ SMART descriptors can quantify steric factors that enable the unique reactivity of dirhodium paddlewheel complexes, such as the absolute pocket volume at each axial catalyst position (*V*_*CAVITY*_) and the extent of hindrance, or entry surface area (*ESA*) exerted on the pocket by surrounding ligands. These descriptors enable quantification of the variation of ligand effects across conformational ensembles.

**Figure 4:**
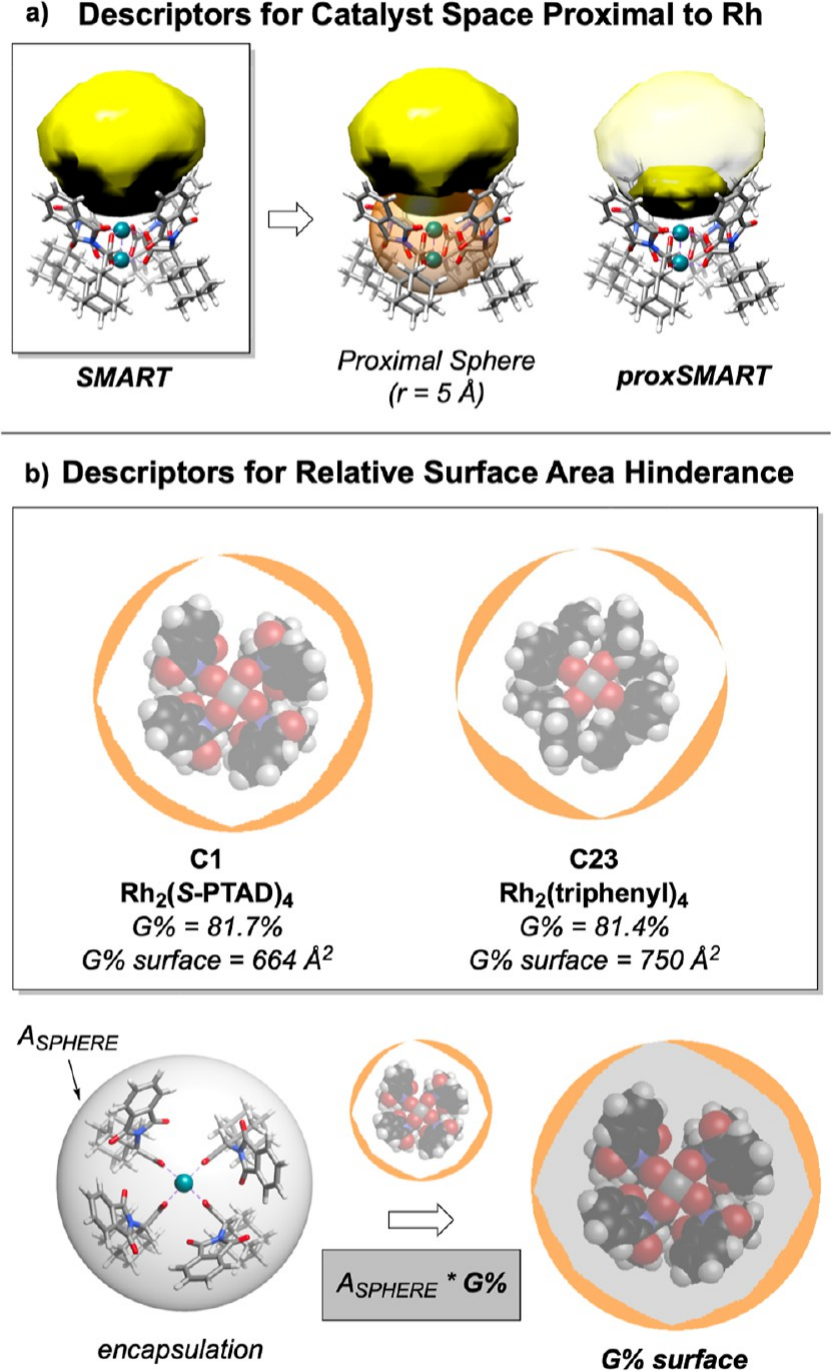
Computation of novel (a) proximal SMART descriptors and (b) *G* % *surface*.

**Figure 5:**
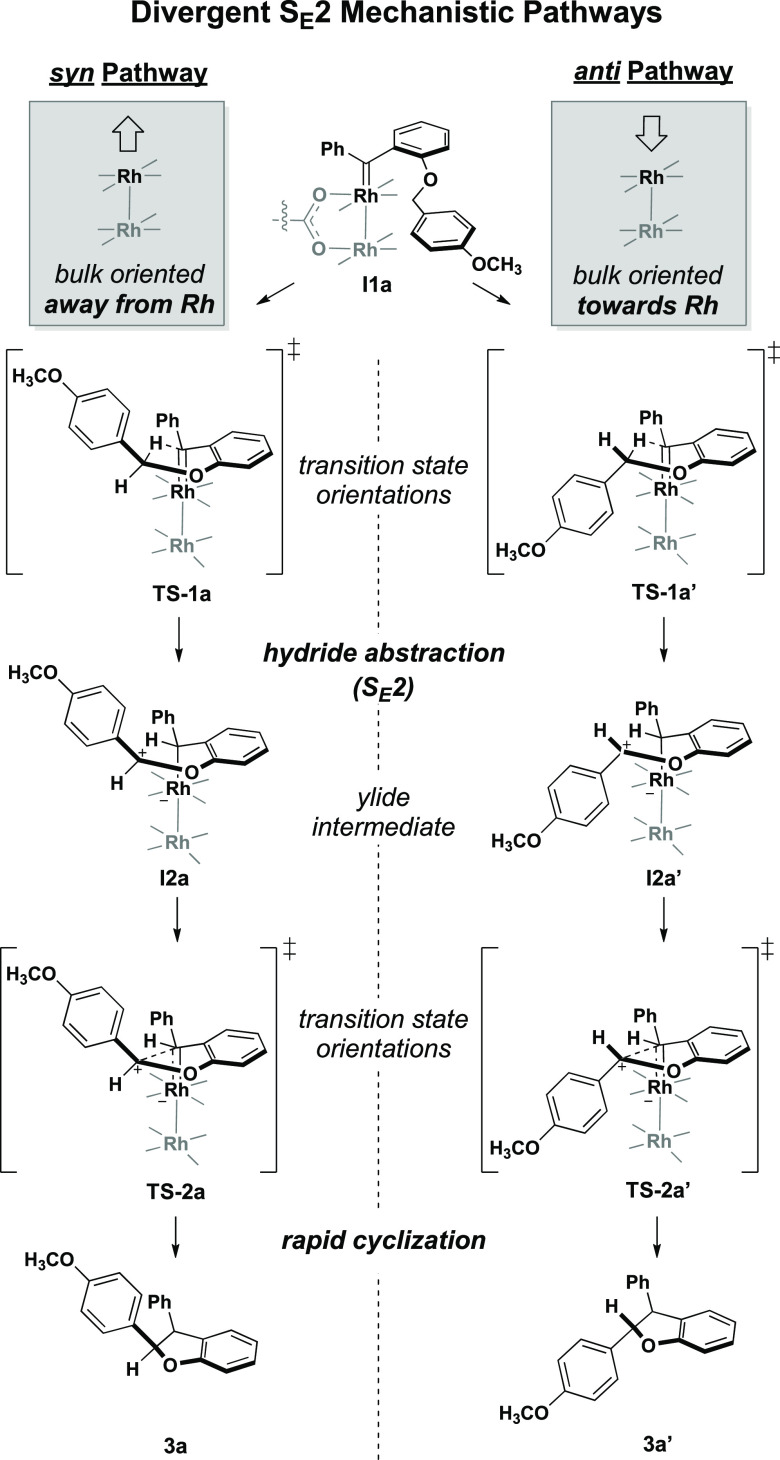
Key intermediates and transition states in the S_E_2 mechanism of C–H insertion.

A modified set of SMART parameters was developed and implemented to capture steric effects near the binding Rh, such as the proximal volume (*proxV*_*CAVITY*_) and proximal ESA (*proxESA*) (Figure 4a). The geometries of the *syn* and *anti*-transition states computed by Shaw and Fox^4^ indicate a reliance upon the spatial orientation of the dirhodium-carbene intermediate **I1a** to separate the divergent pathways (Figure 5). The *syn* pathway is achieved when the substituent is directed upward away from the Rh-binding face, whereas the *anti*-diastereomer forms when this group flips downward. It is hypothesized that **I1** conformationally fluctuates until an orientation promoting cyclization is achieved. Thus, catalyst steric features proximal to the Rh active site were quantified to capture the direct ligand influence on the orientation of cyclization. Similarly, the steric demands of cyclization for each substrate were parameterized from the resultant cyclized products. Steric molecular descriptors were calculated for the ground-state *syn*- and *anti*-products to efficiently simulate the conformational demands of the transition state.

The steric descriptor *G* % developed by Guzei and Wendt^31^ was also utilized to capture ligand hindrance around a metal center by calculating the cone angles of the surrounding ligands. However, this descriptor is measured as an absolute percentage of hindrance inhibiting comparison between catalysts of different sizes. For this purpose, we also developed the descriptor *G* % *surface*, which contextualizes *G* % to the area of a sphere encompassing the catalyst conformer (Figure 4b). The output of *G* % *surface* is the absolute surface area unhindered by ligands, providing a relative comparison of unhindered reactive space for structurally diverse catalysts (other molecular descriptors are described in the Supporting Information).

**Figure 6:**
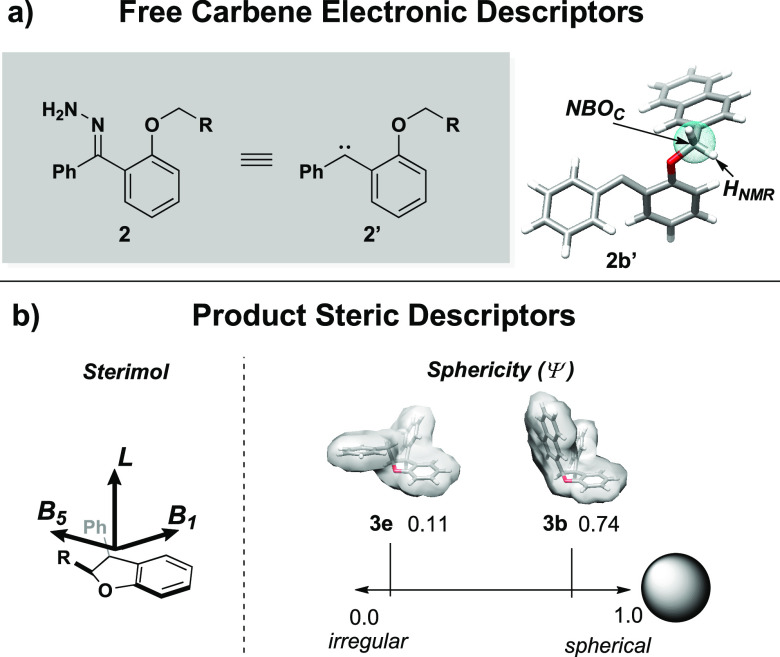
Computation of (a) free carbene electronic descriptors and (b) cyclized product steric descriptors.

Substrate electronic descriptors such as carbene NBO charge (*NBO_C_*) and NMR shifts (*H*_NMR_) were calculated from free carbene surrogates of 2-alkoxybenzophenone substrates (Figure 6a). Since the steric environment required for intramolecular cyclization is product-like (Figure S8), steric descriptors such as Sterimol (*L*, *B*_1_, *B*_5_) and product sphericity (*Ψ*) were computed from conformational ensembles of the ground state *syn*-product (Figure 6b).

### Underperformance of Linear Statistical Models

**Figure 7:**
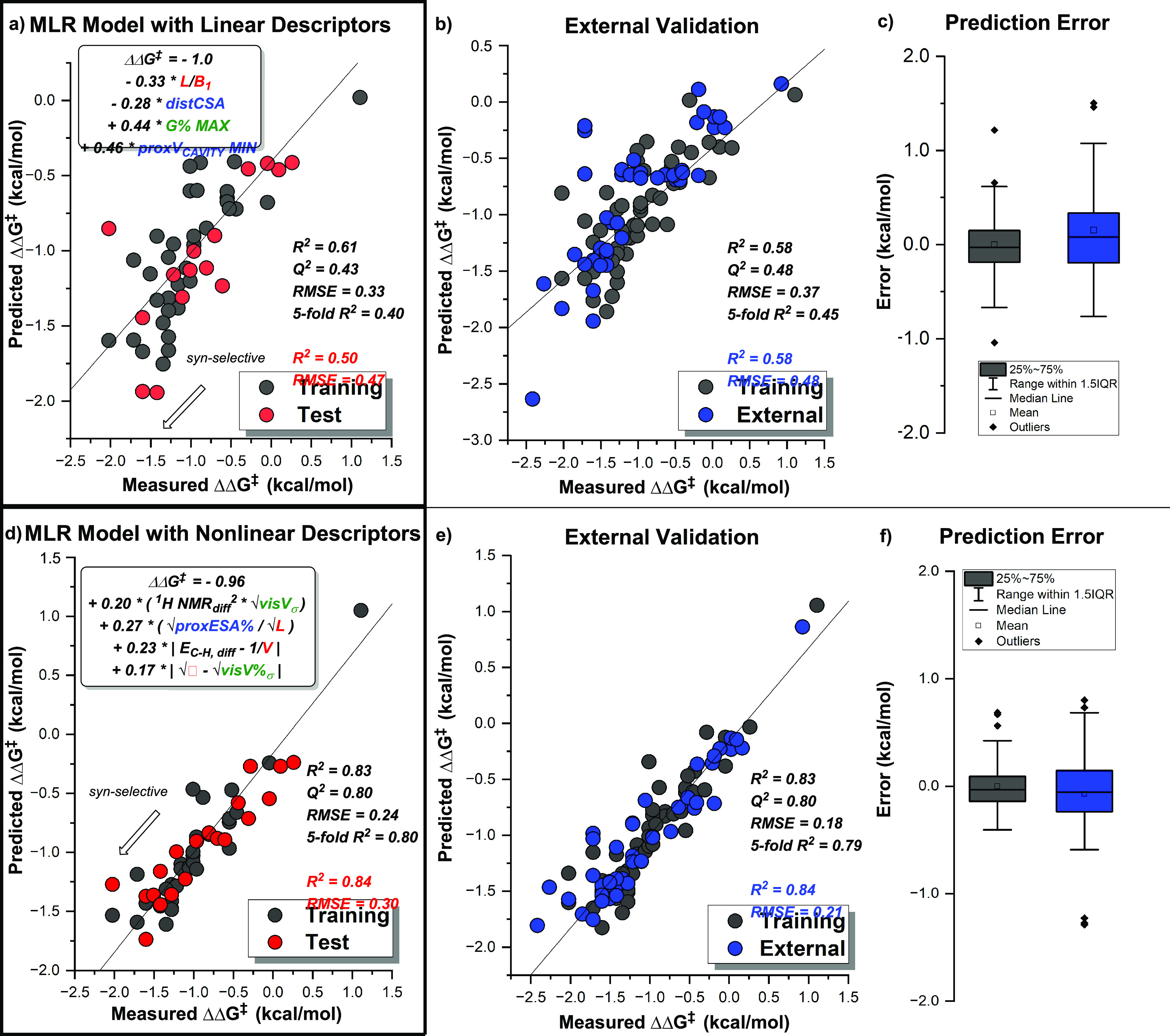
(a) Performance of the best MLR model obtained by regressing linear parameters, where more negative values correlate to higher *syn*-selectivity. Training data shown in black (*R*^2^ = 0.61, *Q*^2^ = 0.43, fivefold *R*^2^ = 0.40, RMSE = 0.33) and test data shown in red (*R*^2^ = 0.50, RMSE = 0.47) and (b) performance of a linear model on external test data. Training data are shown in black (*R*^2^ = 0.58, *Q*^2^ = 0.48, fivefold *R*^2^ = 0.45, RMSE = 0.37), and external data are shown in blue (*R*^2^ = 0.58, RMSE = 0.48). (c) Statistical spread of prediction error for the training (black) and external (blue) data sets. (d) Performance of the best MLR model obtained by regressing nonlinear parameters generated by the SISSO algorithm. Training data are shown in black (*R*^2^ = 0.83, *Q*^2^ = 0.80, fivefold *R*^2^ = 0.80, RMSE = 0.24), and test data are shown in red (*R*^2^ = 0.84, RMSE = 0.30). (e) Performance of a nonlinear model on external test data. Training data are shown in black (*R*^2^ = 0.83, *Q*^2^ = 0.80, fivefold *R*^2^ = 0.79, RMSE = 0.18), and external data are shown in blue (*R*^2^ = 0.84, RMSE = 0.21). (f) Statistical spread of prediction error for the training (black) and external (blue) data sets.

As the next step, the experimental data were combined and regressed with the DFT-level descriptors gathered for the conformational ensembles using MLR. A 50% training/test split was employed for model development using a y-equidistant algorithm to select the training set. From the training set of 43 data points, a linear model was developed with modest statistical accuracy ( training *R*^2^ = 0.61, *Q*^2^ = 0.43) (Figure 7a). Modest accuracy was also observed for the external validation set (test *R*^2^ = 0.58) (Figure 7b,c). Substrate and catalyst effects for similar subsets of data were modeled well, while more diverse structures exhibited large errors (Figures S14 and S15). This result supported the conclusion that the nature of catalyst–substrate interactions influencing diastereoselectivity is complex and dynamic. Essentially, the changing requirements for the features of both reaction components cannot be accurately quantified by traditional linear models.

### Performance of Nonlinear Descriptor Modeling

As a result, we turned to the recently developed sure independence screening and sparsifying operator (SISSO) with the goal of capturing nonlinear effects.^38^ This algorithm performs a series of algebraic functions on the original molecular descriptors set, casting the original molecular descriptors into a set of nonlinear parameters that can be regressed using MLR techniques. A modified version of the SISSO algorithm was applied to generate nonlinear parameters from the DFT descriptors (Supporting Information). The same training/test splitting method and external validation sets used previously were employed. A top performing model (training *R*^2^ = 0.83, *Q*^2^ = 0.80, fivefold *R*^2^ = 0.80, RMSE = 0.24) (Figure 7d) was identified as capable of predicting diastereoselectivity for the external validation set with significantly improved accuracy over linear modeling efforts (test *R*^2^ = 0.84, RMSE = 0.30) (Figure 7e,f).

**Figure 8:**
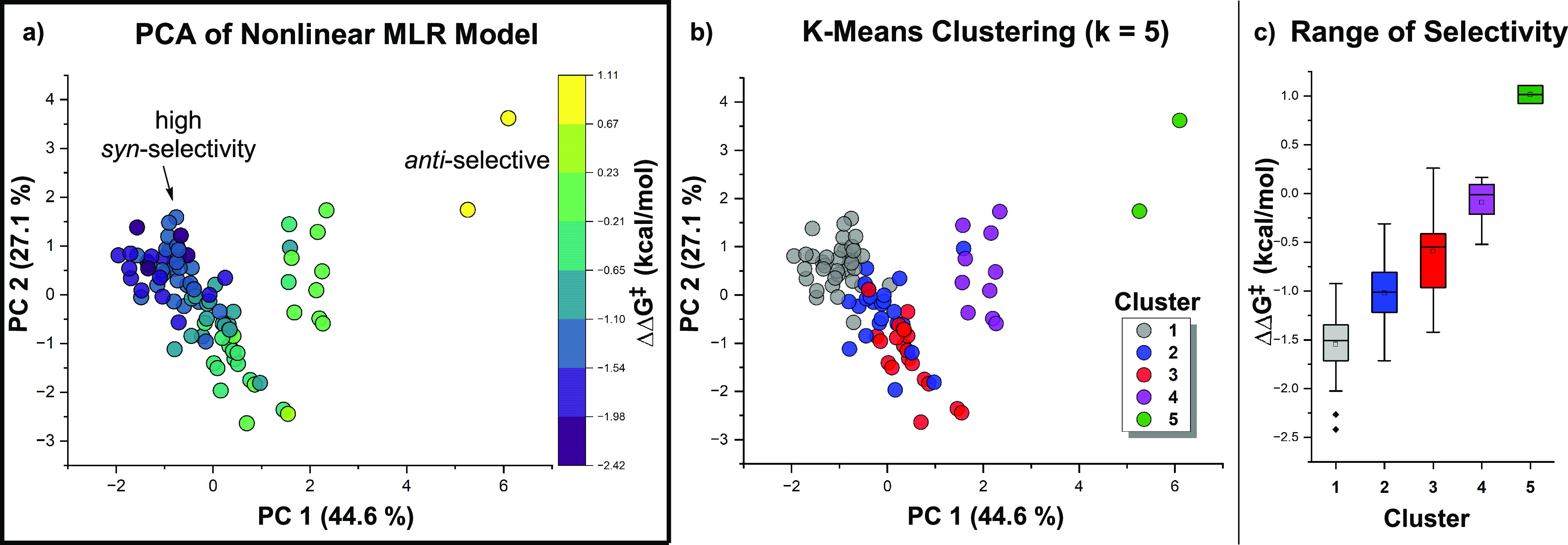
(a) PC space map of the data matrix constructed from nonlinear model parameters with percent variance of the first two PCs. (b) K-means clustering (*k* = 5) divides the space into distinct clusters, with the corresponding diastereoselectivity ranges shown in (c).

Interpretation was initially aided by visualizing the decisions made by the model to partition the data into poorly and highly diastereoselective regions. This was accomplished through the construction of a PC chemical space map from four nonlinear model parameters (Figure 8a). Using the K-means algorithm, the space was divided into five clusters (Figure 8b), which intuitively divided the chemical space according to the trends in observed diastereoselectivity (Figure 8c).

Three major groups are observed in the PC space. The largest island contains data points containing mainly *syn*-selective reactions with a tail region of lower selectivity. Within this island, cluster 1 (gray) contains the reactions yielding the highest *syn*-selective data points, while clusters 2 (blue) and 3 (red) have decreased average selectivity and a larger range of ΔΔ*G*^‡^. The smallest and most distal group (cluster 5, green) contains the two reactions that produce *anti*-selective results, and an island of nonselective data (cluster 4, purple) lies between the two highly selective regions. Due to the nonlinear nature of the data set, subsets of data representing distinct trends in selectivity were analyzed together to deconvolute complex interaction effects into mechanistically interpretable explanations for differing reaction outcomes.

### Catalyst and Product Features Leading to High *syn*-Selectivity

**Figure 9:**
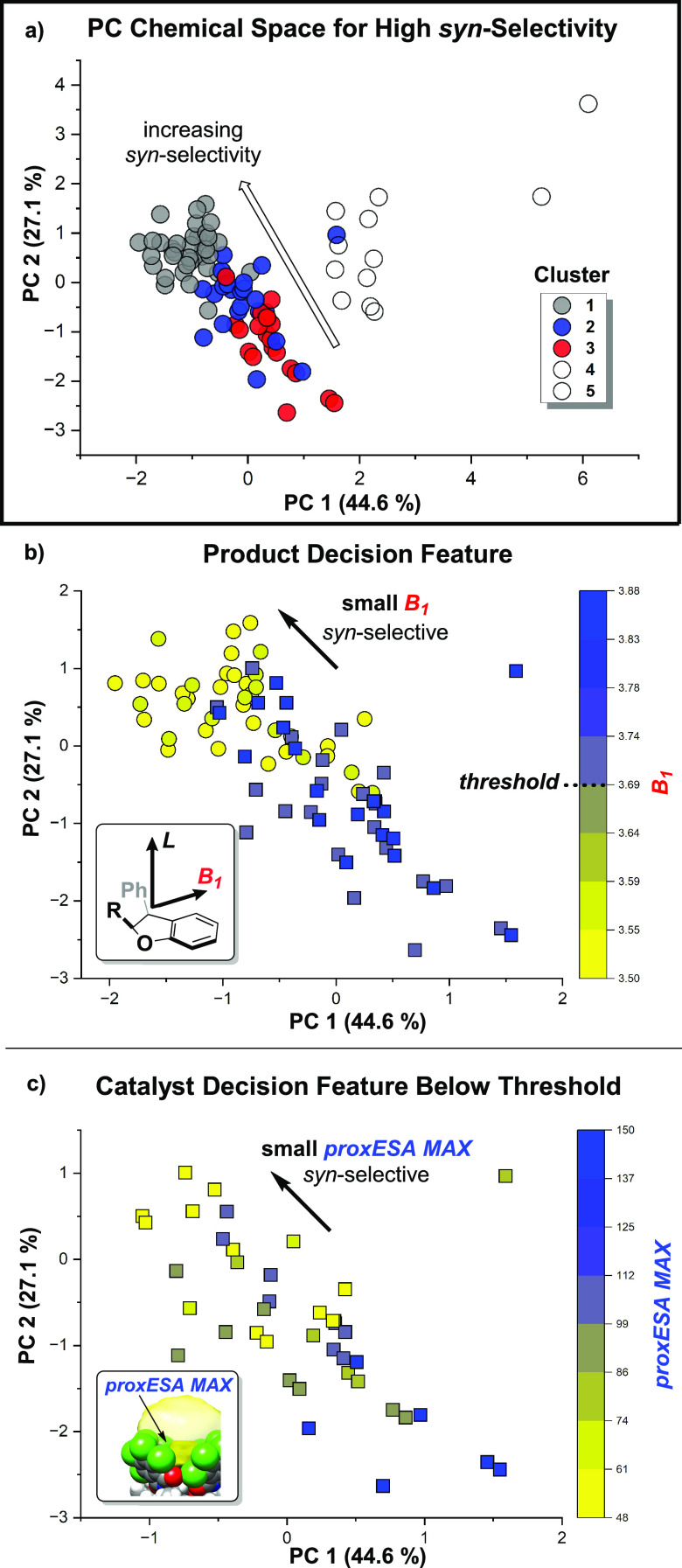
Decision tree describing the relationships between catalyst and substrate interactions and their implications on the degree of *syn*-selectivity in (a) clusters 1–3. (b) Decision tree node for the product descriptor *B*_1_. A *B*_1_ value smaller than the threshold value (circle, ≤3.69 Å) correlates to higher *syn*-selectivity. Points above the threshold (square, >3.69 Å) can still be *syn*-selective. (c) Decision tree node for the catalyst descriptor *proxESA MAX*. Data above the *B*_1_ threshold are further sorted by catalyst features. A small *proxESA MAX* leads to higher *syn*-selectivity.

Clusters 1–3 contain data points with the greatest spread of dr, spanning from highly *syn*-selective to unselective [ΔΔ*G*^‡^ (kcal/mol) < −2.4–0.2, dr >97:3–39:61] (Figure 9a). As the next step, we assessed which molecular descriptors are the most influential for selectivity by constructing a decision tree (Figure S18). The first node of this decision tree divides outcomes based on the Sterimol parameter *B*_1_ of the *syn*-product. Plotting this descriptor in PC space reveals that reactions yielding products with high *syn*-selectivity generally exhibit a small *B*_1_ value (Figure 9b, circle). This suggests that narrow *syn*-products (small *B*_1_) inherently encourage higher *syn*-selectivity.

Interestingly, several outlier points with a large *B*_1_ value still yield high *syn*-selectivity (Figure 9b, square). A second decision node defined by the catalyst descriptor *proxESA MAX* provides insights into how steric hindrance can override the innate propensity of the substrate to control the product diastereoselectivity. Outlier points with large product *B*_1_ values are paired with catalysts possessing a low *proxESA MAX* value (Figure 9c), leading to an increase in *syn*-selectivity. A smaller *proxESA MAX* for a catalyst promoting *syn*-selectivity supports the previous hypothesis that more hindered catalysts better differentiate diastereomeric transition states and are more selective.

**Figure 10:**
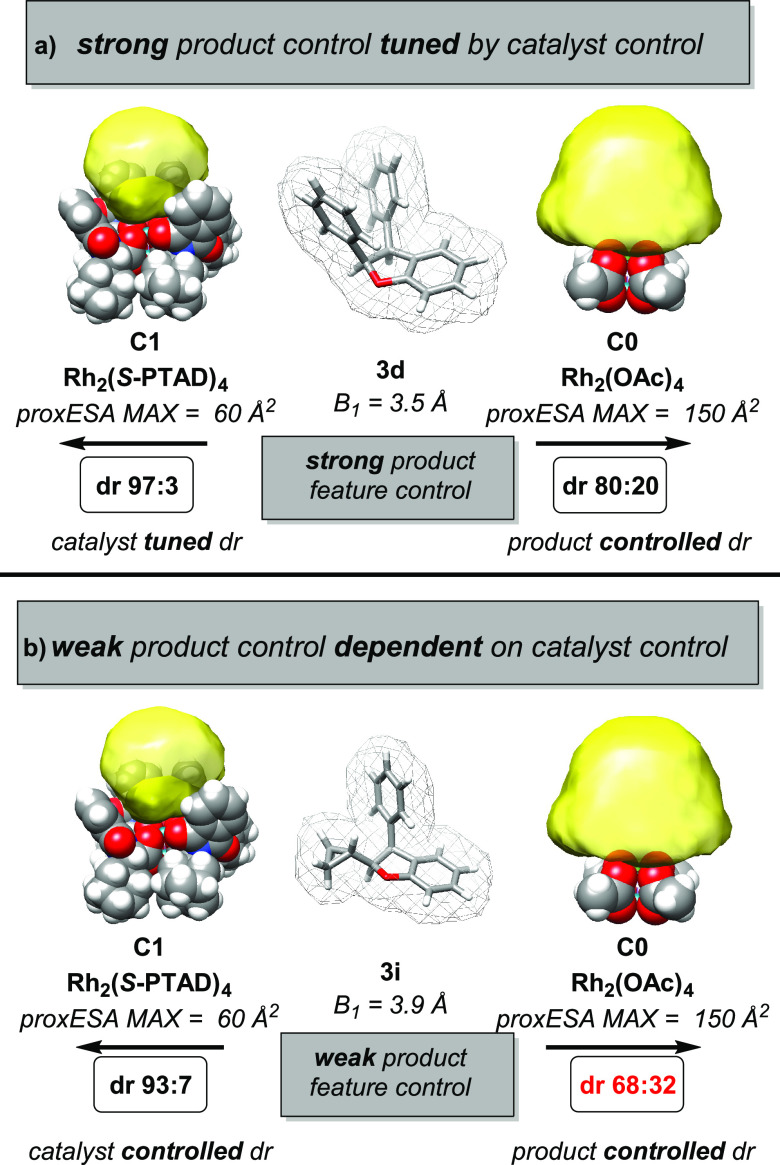
Catalyst feature interactions with product features in clusters 1–3. (a) Product steric feature *B*_1_ dictates intrinsic *syn*-selectivity preference. (b) Catalyst feature *proxESA MAX* can be used to override or fine-tune this preference for *syn*-selective optimization.

In the analysis of the molecular features for optimizing *syn*-selectivity, substrates are shown to possess an innate preference for diastereoselectivity based on the steric features of their resultant products. Favorable product formation can be tuned for optimal *syn*-selectivity through increasing catalyst hindrance (Figure 10a). Additionally, pairing unfavorable product features with a more hindered catalyst can overcome this preference and result in *syn*-selectivity for an otherwise unselective substrate (Figure 10b). This substrate control vs catalyst control was similarly observed by one of our teams in the C–H insertion of stereogenic centers.^39^

### Catalyst and Product Features Leading to High *anti*-Selectivity

**Figure 11:**
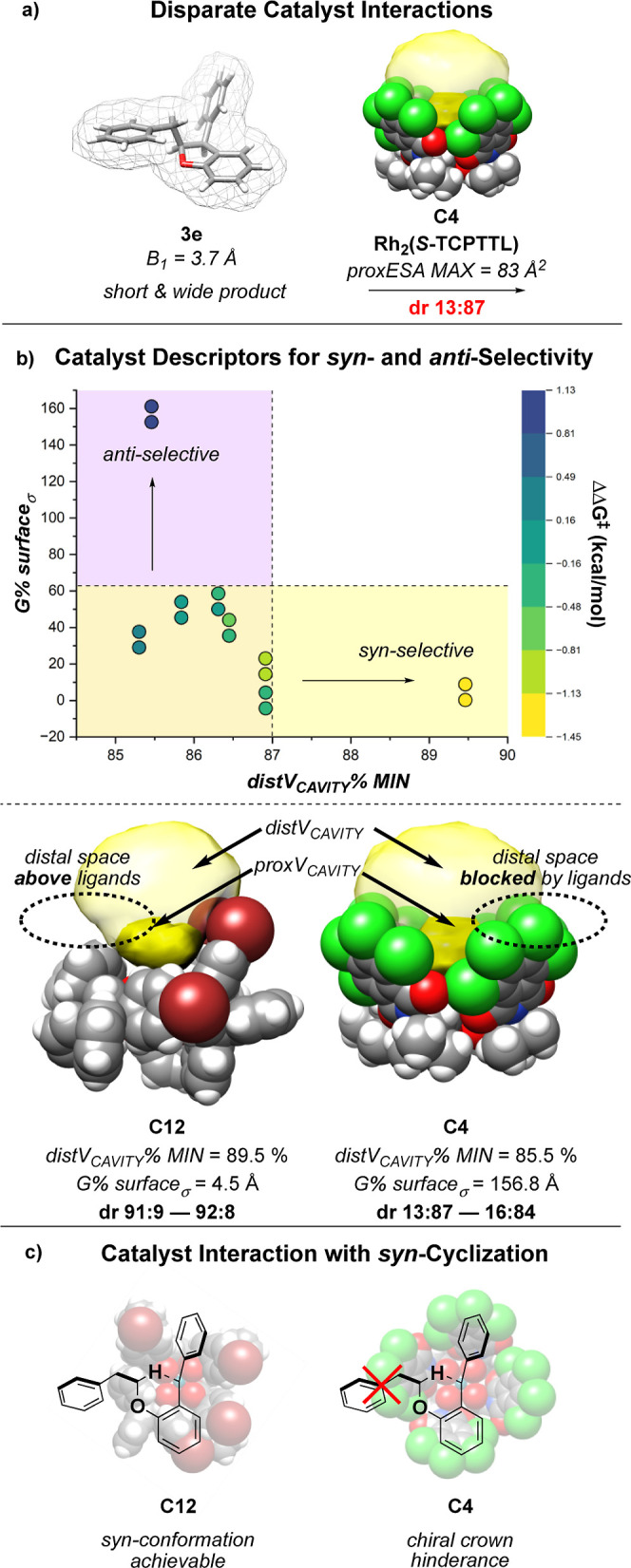
(a) Disparate trends of the diastereoselectivity in products **3e**. (b) Catalyst descriptors *G* % *surface*_σ_ and *distV*_*CAVITY*_ % *MIN* dictate the differential *syn*- and *anti-*selectivity observed with substrates **2e** and **2f**. (c) Increased distal steric hindrance blocks the formation of the *syn*-transition state.

Though optimizing *syn*-selectivity was our initial goal, two unusually *anti*-selective reactions were observed with the hindered catalyst Rh_2_ (*S*-TCPTTL) (**C4**) that was previously shown to promote high *syn*-selectivity. Substrates **2e** and **2f** show a divergence from the previously assessed catalyst-product-feature pairing guidelines. The minimum widths of **3e** (*B*_1_ = 3.7 Å) and **3f** (*B*_1_ = 3.7 Å) suggest that these products are unselective. However, pairing these substrates with the hindered catalyst **C4** results in *anti*-selectivity (Figure 11a).

To expose the source of this divergence, two descriptors were identified that capture the interaction of catalyst effects with products **3e** and **3f**. Catalyst features quantifying the minimum percentage of distal pocket volume (*distV*_CAVITY_ % *MIN*) and the weighted standard deviation of the catalyst hindrance (*G* % *surface*_σ_ ) were found to be influential on the outcome of cyclization (Figure 11b).

The descriptor *distV*_*CAVITY*_ % *MIN* describes the ratio of the total pocket volume between the regions proximal and distal to Rh. A larger minimum percentage of distal volume promotes high *syn*-selectivity for these substrates. This is likely attributed to the demanding shape of transition state **TS-1**. The substituent bulk of **3e** and **3f** is oriented perpendicularly to the Rh-binding axis, resulting in an atypically wide *syn*-product. Optimal catalyst **C12** has a large *distV*_*CAVITY*_ % *MIN*, allowing the substrate to adopt the necessary wide conformation for *syn*-cyclization over the top of the ligands. Smaller *distV*_*CAVITY*_ % *MIN* leads to significantly less *syn*-selectivity, as the area distal to Rh cannot accommodate *syn*-cyclization.

*G* % *surface*_σ_ describes the relative flexibility of the catalyst pocket by how variable the hindrance is throughout a conformational ensemble. A larger *G* % *surface*_σ_ indicates that the positions of ligands in hindered conformations deviate more significantly through ligand bond rotation. **C4** is one of the most hindered catalysts in this case study and has a low *distV*_*CAVITY*_ % *MIN.* This large hindrance at the distal region may block the formation of the wide *syn*-transition state. **C4** also has an unusually high *G* % *surface*_σ_. Thus, the conformation can significantly fluctuate, interrupting the network of ligand interactions that leads to high hindrance. The *anti*-cyclization observed with this catalyst is hypothesized to be dependent upon the slipping or rotation of the ligands away from the chiral crown conformation, allowing the formation of **TS-1′** by the downward orientation of the substituent.

Products **3e** and **3f** have distinct shape requirements that interact with the catalysts differently from the other substrates analyzed previously. Catalyst features defining the amount of distal pocket space and the variability of steric hindrance were found to be influential to the observed outcome. In these cases, more distally hindered catalysts resulted in low selectivity, while low distal hindrance promoted *syn* selectivity by allowing cyclization to occur over the top of the ligands. *Anti*-cyclization occurs when hindered catalysts are flexible and slip out of highly hindered states, creating a large enough opening between ligands for the *anti*-conformation to form.

### Feature Interactions for Globally Optimizing Diastereoselectivity

**Figure 12:**
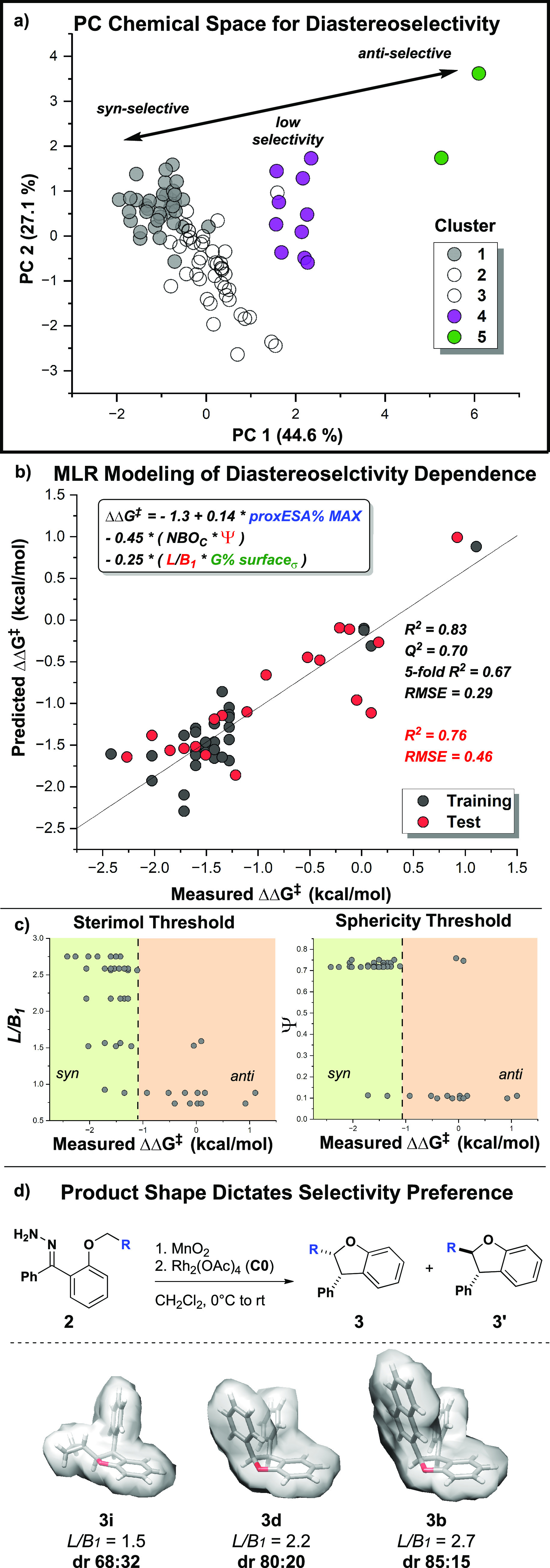
MLR modeling of catalyst and substrate effects in (a) clusters 1, 4, and 5. (b) MLR model for optimization of diastereoselectivity. (c) Cross terms of the MLR model. Both show a dependence on product steric features, *L*/*B*_1_ and Ψ. (d) Relationship between substrate steric feature *L*/*B*_1_ and diastereoselective preference.

With specific interactions explaining cases of both high *syn*- and *anti*-selectivity, we then revisited the use of MLR to generate a model providing a global understanding of the relationship between catalyst–substrate interactions and diastereoselectivity. Clusters 1 (gray, ΔΔ*G*^‡^ (kcal/mol) < −2.4 to −0.92), 4 [purple, ΔΔ*G*^‡^ (kcal/mol) −0.52–0.16], and 5 [green, ΔΔ*G*^‡^ (kcal/mol) 0.92–1.1] were regressed together to minimize the nonlinear effects from substrate vs catalyst control for *syn*-selectivity (Figure 12a). A training/test split of 40% by the Kennard–Stone algorithm on a data set of 49 points resulted in a model with one linear catalyst descriptor and two interaction terms (Figure 12b). This model performs well (training *R*^2^ = 0.83, *Q*^2^ = 0.70, fivefold *R*^2^ = 0.69, RMSE = 0.29) and can predict test points with good accuracy (test *R*^2^ = 0.76, RMSE = 0.46).

**Figure 13:**
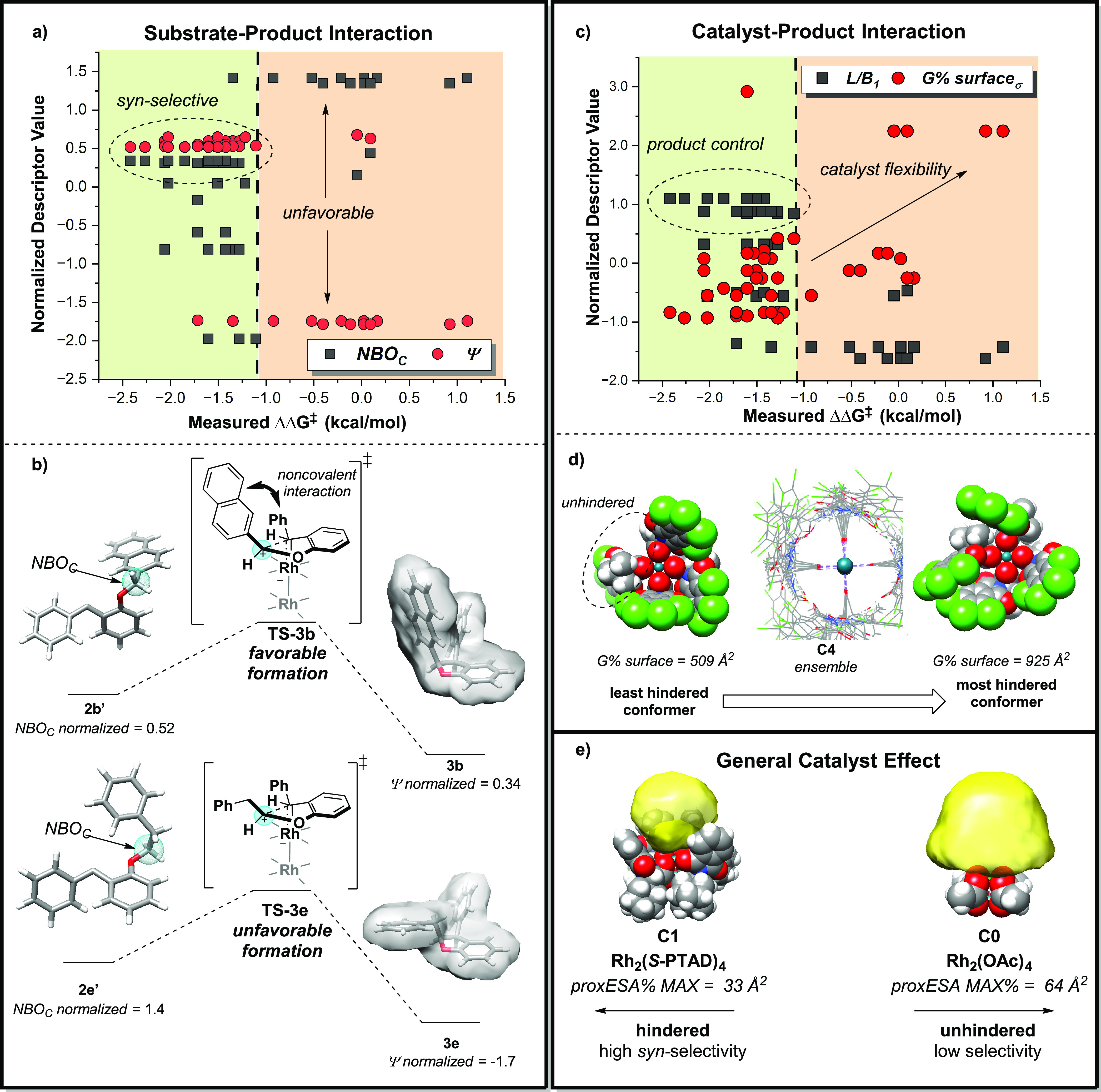
Summary of interaction effects and impact of molecular features on diastereoselectivity. (a) Interaction term of the product steric descriptor Ψ and free carbene electronic descriptor *NBO_C_*. (b) Impact of substrate–product interaction on the *syn*-cyclization pathway. (c) Interaction term of catalyst descriptor *G* % *surface*_σ_ and product steric descriptor *L*/*B*_1_. (d) As catalyst flexibility increases, conformers may become unhindered enough to promote *anti*-cyclization. (e) General catalyst influence for tuning the diastereoselectivity depends upon *proxESA* % *MAX*.

The model asserts that a lower maximum percentage of the proximal ESA (*proxESA* % *MAX*) correlates to higher *syn*-selectivity. A low *proxESA* % *MAX* conveys that the percentage of the unhindered surface area of the pocket proximal to Rh is small for the least hindered conformer. Thus, a small *proxESA* % *MAX* indicates that a catalyst is highly hindered regardless of the conformation accessed, reflecting a previous analysis that more hindered catalysts promote high *syn*-selectivity (Figure 13e).

Two interaction terms were found to be required to build a statistically sound model. Both exhibit dependence on the steric properties of the *syn*-product (Figure 12c). The descriptor L/*B*_1_, as seen in a previous analysis, can be interpreted as a measure of compactness depending on the orientation of the substituent bulk. The sphericity of the *syn*-product (Ψ) is another measure of compactness calculated from the surface area and volume of the molecule. A more detailed understanding of these descriptors can be found by plotting them directly as a function of ΔΔ*G*^‡^. Essentially, the descriptors act to classify how a less compact product yields a lower *syn*-selectivity (Figure 12d).

Of the remaining descriptors from the interaction terms, the substrate descriptor *NBO_C_* defines the NBO charge at the insertion carbon, computed from the lowest energy conformer of the analogous free carbene. When the normalized descriptor values of *NBO_C_* and Ψ were plotted as a function of ΔΔ*G*^‡^, a relationship between product shape and substrate electronics was revealed (Figure 13a). It is hypothesized that the interaction between *NBO*_*C*_ and Ψ captures the favorability of achieving the *syn*-transition state depending on the conjugative effects of the substituent of the free carbene and the shape of the target product (Figure 13a). The favorability of a larger electron density captures the conjugative effects of the alkoxy substituent on the insertion carbon. Substituents with more π conjugation lead to more negative NBO charges. This effect is hypothesized to indicate the extent of noncovalent interactions between the alkoxy substituent and the adjacent aryl group (Figure 13b). Stronger interactions may encourage the formation of **TS-3**. This is supported by trends of product structure across the nonlinear modeling PC space where aryl substituents are found to be inherently *syn*-selective, whereas alkyl substituents have a broader range of diastereoselectivity that can be overridden by catalyst effects (Figure S19), and by comparison of transition state energies for different substrates (Figure S21).

To illustrate this, an inherent *syn*-favorability is observed for **3b** as large *L*/*B*_1_ (2.7 Å) and Ψ (0.72) describe a narrow and compact product. This is aided by higher electron density at the insertion carbon of the free carbene substrate **2b′** (NBO_C_ = −0.029) due to increased conjugative effects from the naphthyl substituent. Strong noncovalent interactions between substituents lead to the favorable formation of **TS-3b**, while the product shape further indicates a lower barrier to the formation of the cyclized *syn*-product (Figure 12b). Conversely, **3e** has a small *L*/*B*_1_ (0.88) and Ψ (0.11), describing a wide and irregular product. The positive *NBO_C_* (2.0 × 10^5^) further indicates less electron density at the insertion carbon of **2e′**. These effects cumulate as an increased difficulty for the formation of **TS-3e**, further exacerbated by an unfavorable *syn*-product shape. This overall leads to an intrinsic unfavorability for *syn*-cyclization in **2e**, which can be overridden or fine-tuned with catalyst effects.

The second interaction term, *G* % *surface*_σ_, was defined previously in the analysis of the origins of *anti*-selectivity. In this model, the product descriptor *L*/*B*_1_ interacts with *G* % *surface*_σ_ (Figure 13c). As assessed previously, substrates with a large *L*/*B*_1_ are intrinsically *syn*-selective, so the catalyst descriptor *G* % *surface*_σ_ does not override the selectivity of the product, dominating overall diastereoselectivity. Rather, this interaction largely dictates the interactions in products with weak control. Unfavorable products (small *L*/*B*_1_) paired with a catalyst with a low *G* % *surface*_σ_ result in an overall low *syn*-selectivity. The reactions become more unselective with increased flexibility. This is reflected by the linear catalyst descriptor *proxESA* % *MAX*, as more hindered catalysts paired with intrinsically unselective products increase *syn*-selectivity. *Syn*-selectivity decreases with higher catalyst flexibility as the catalyst is no longer able to override the intrinsic product unfavorability, as pockets of unhindered space about the Rh may form more consistently (Figure 13d).^4,26,37^

## Conclusions

In summary, we developed a method of analysis for nonlinear catalyst–substrate interaction effects and their impact on diastereoselectivity. Using a diverse array of dirhodium(II) catalysts and substituted 2-alkoxybenzophenone substrates, we constructed a diverse experimental reaction matrix with the aid of data science tools. These components were computed individually, and reactions were modeled combinatorially using the nonlinear SISSO algorithm. This allowed the complex interplay between catalyst and substrate features to be mechanistically deconvoluted, and the component interactions leading to *syn*- and *anti*-selective reactions were revealed through MLR.

It was found that product effects largely dictate the range of diastereoselectivity observed for a given substrate, while more subtle catalyst effects come into play within that range. Two unusually *anti*-selective reactions were found to be the result of divergent catalyst features that are important in the interaction with cyclized product sterics. The mechanistic codependency of catalyst and substrate features presented serves to offer key insights into design features for expedited optimization of donor/donor carbene cyclizations through C–H insertion and presents a blueprint for harnessing the performance of nonlinear modeling methods without sacrificing mechanistic interpretability. Future applications of these nonlinear analysis techniques will be applied to interrogate other complex interactions between reaction components in catalysis.

## Supporting Information Available

Crystal structure of **C14** (CIF)
Crystal structure of **C16** (CIF)
Crystal structure of **C15** (CIF)
MLR and SISSO algorithm input data (XLSX)
Catalyst characterization, synthetic details, computational details, and supplementary discussion (PDF)

## Supplementary Material

cs3c04256_si_001.cif

cs3c04256_si_002.cif

cs3c04256_si_003.cif

## Abbreviations

MLR: multivariate linear regression
PCA: principle component analysis
