# Supplementary material for: Deconvoluting Nonlinear Catalyst–Substrate Effects in the Intramolecular Dirhodium-Catalyzed C–H Insertion of Donor/Donor Carbenes Using Data Science Tools
Source: ACS Catal. 2023 Dec 11;14(1):104–15. doi: 10.1021/acscatal.3c04256 (PMC10775150; doi:10.1021/acscatal.3c04256)
Supplement: Supplementary file 5 — cs3c04256_si_005.pdf [file cs3c04256_si_005.pdf]

Supporting Information for

Deconvoluting Nonlinear Catalyst-Substrate Effects in the Intramolecular Dirhodium-Catalyzed C–H Insertion of Donor/Donor Carbenes using Data Science Tools

Lucas W. Souza[2‡], Beck R. Miller[1‡], Ryan C. Cammarota[1], Anna Lo[2], Ixchel Lopez[2], Yuan-Shin Shiue[2], Benjamin D. Bergstrom[2], Sarah N. Dishman[2], James C. Fettinger[2], Matthew S. Sigman[1]* and Jared T. Shaw[2]*.

[1]Department of Chemistry, University of Utah, Salt Lake City, UT 84112, United States

[2]Department of Chemistry, University of California, Davis, CA 95616, United States

Email: jtshaw@ucdavis.edu
matt.sigman@ utah.edu

Table of Contents

I. General Considerations and Instrumentation……………………………………….S1
II. General Procedures………………………………………………………………S1
III. Computational Methods and Details……………………………………………. S11
   a. Dirhodium(II) Complexes………………………………………………. S11
   b. Calculation of Catalyst Molecular Descriptors…………………………..S14
   c. Substrates and *syn*-Products…………………………………………….. S19
   d. Selection of Diverse Structures by PCA…………………………………S21
   e. Linear Modeling Discussion……………………………………………..S24
   f. Nonlinear Parameter Generation and Modeling…………………………S30
   g. Hydride Abstraction Transition State Calculations………………………S33
IV. References………………………………………………………………………..S37
V. Spectral Data……………………………………………………………………..S40

I. General Considerations and Instrumentation

Chemicals were purchased and used without further purification. Solvents were dried on a JC Meyer solvent system or purchased anhydrous where required. Reactions requiring anhydrous conditions were performed under argon; glassware was flame dried under vacuum immediately prior to use and allowed to cool under reduced pressure; liquid reagents, solutions or solvents were added via syringe through rubber septa; solid reagents were added under a flow of argon. Reactions were monitored by TLC on Kieselgel 60 F254 (Merck) plates and detected by examination under UV light (254 nm and 365 nm). Flash column chromatography was performed using silica gel [Merck, 230−400 mesh (40−63 μm)], unless otherwise stated. Microwave reactions were conducted using a Biotage Initiator^{TM} 2.0, employing 2.45 GHz microwaves. Accurate mass measurements were recorded on positive ESI mode in methanol. Extracts were concentrated *in vacuo* using both a rotary evaporator at a pressure of 15 mmHg (diaphragm pump), and a high vacuum line at a pressure of 0.1 mmHg (oil pump) at room temperature. ^{1}H and ^{13}C spectra were measured in the solvent stated at 400 or 600 MHz and 101 or 151 MHz respectively. ^{1}H and ^{13}C NMR chemical shifts are quoted in parts per million (ppm) and referenced to TMS (TMS: ^{1}H = 0.0 ppm and ^{13}C = 0.0 ppm), coupling constants (*J*) are given in Hertz (Hz). Multiplicities are abbreviated as: br (broad), s (singlet), d (doublet), t (triplet), q (quartet), p (pentet) and m (multiplet) or combinations thereof. Diasteroselectivties were measured by ^{1}H NMR. Catalysts with known structures were obtained by donation from the laboratory of Professor Joe Fox or were purchased when commercially available.[1–3]

II. General Procedures

General Procedure A for alkylation. To a flame dried 50 mL round bottom flask was added 2-hydroxybenzophenone (1.0 equiv.), purged under high vacuum, and backfilled with argon. To the flask was added dry acetonitrile (0.1M), the respective bromide (1.3-1.5 equiv.), and 12 hour oven dried Cs_2CO_3 (3 equiv.). The reaction was stirred at 50 ° C for 12 hours. The reaction mixture was allowed to cool, and solvent was removed by rotatory evaporation. The residue was then resuspended in dichloromethane and washed with deionized water. The organic layer was dried over Na_2SO_4, filtered, and solvent was removed in vacuo. The resulting crude material was purified by flash column chromatography to yield the desired ketone.

General Procedure B for hydrazone formation. To a solution the respective ketone (1 equiv) in anhydrous EtOH (0.1M) was added AcOH (2 equiv) and anhydrous hydrazine (10 equiv). The reaction mixture was heated in a microwave reactor at 160 °C for 1-3 hours. The reaction was allowed to cool then diluted with Et_2O and washed with water. The organic layer was removed and the aqueous layer was washed three times with Et_2O. The combined organic layer was washed with water and dried over Na_2SO_4. The crude reaction mixture was purified by flash column chromatography to yield the desired hydrazone.

General Procedure C (two-pot insertion). To a flame-dried scintillation vial under argon atmosphere was added the desired hydrazone (1 equiv) followed by anhydrous CH_3CN (0.01 M). To the vial was added MnO_2 (8 equiv). The resulting dark suspension was stirred until full conversion of the starting material was observed by TLC. The reaction mixture was filtered over Celite into a new flame-dried, argon backfilled 20 mL scintillation vial using the same solvent. The magenta solution was cooled to 0 °C and the desired rhodium catalyst was added (0.01 equiv). The reaction mixture was warmed to room temperature and allowed to stir from 10 min to 12 h.

The crude reaction mixture was concentrated *in vacuo* and purified by flash column chromatography to yield the desired insertion product. Diastereomeric ratios were determined by crude ^{1}H NMR in which conversion appeared to be greater than 90%. For known compounds, only general experimentals and ^{1}H NMR data were reported. For novel compounds, full chacterization and experimental data has been reported including isolated yields. Absolute and relative stereochemistry has been confirmed in previous work from our lab.[4–7]

General Procedure D for Catalyst Synthesis
To a flame dried 50 mL round bottom flask was added $Rh_2(OAc)_4$ (**C0**) (1 equiv) followed by the requisite ligand (4.1 equiv) and chlorobenzene. To the flask was added a 100 mL addition funnel filled with oven dried sodium bicarbonate (30 equiv). A cotton plug was placed in the stopcock in order to prevent solid from falling into the reaction. The stopcock was opened, and the reaction was heated to reflux. A heat gun was used to heat up the glass and the entire reaction vessel was wrapped in aluminum foil. (If working correctly, solvent should be refluxing through the arm of the addition funnel and dripping down through the bicarbonate and back into the reaction flask.) After 12 hours the reaction mixture was cooled and diluted in Ethyl Acetate (100 mL). The organic layer was washed with sat. $NaHCO_3$ 3x25 mL and 3x with water. The aqueous layer was back extracted with 3x 25 mL of EtOAc. The organic layer was combined, dried over anhydrous Na_2SO_4, filtered, and solvent was removed by rotary evaporation to yield the crude product.

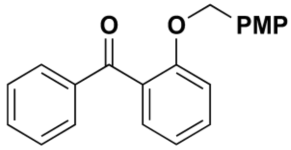

(2-((4-methoxybenzyl)oxy)phenyl)(phenyl)methanone (**1a**) was synthesized according to general procedure A using 2-hydroxybenzophenone (1 g, 5 mmol), 1-(bromomethyl)-4-methoxybenzene (947 mg, 6 mmol), Cs_2CO_3 (4 g, 15 mmol) and CH_3CN 15 mL). The crude product was purified by flash column chromatography (40:60, CH_2Cl_2:Hexanes) affording **1a** as a clear oil (1.1 g, 72 %). Proton NMR data matched previously reported literature values.[5] ^{1}H NMR (599 MHz, $CDCl_3$) δ 7.80 (d, *J* = 7.7 Hz, 2H), 7.55 (t, *J* = 7.5 Hz, 1H), 7.48 – 7.38 (m, 4H), 7.12 – 6.99 (m, 2H), 6.89 (d, *J* = 8.2 Hz, 2H), 6.72 (d, *J* = 8.1 Hz, 2H), 4.92 (s, 2H), 3.75 (s, 3H).

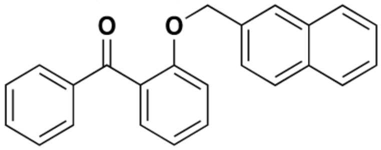

(2-(naphthalen-2-ylmethoxy)phenyl)(phenyl)methanone (**1b**) was synthesized according to general procedure A using 2- hydroxybenzophenone (2 g, 10 mmol), 2-napthylbromide (2.6 g, 12 mmol), Cs_2CO_3 (9.6 g, 30 mmol) and CH_3CN 15 mL). The crude product was purified by flash column chromatography (90:10, Hexanes:EtOAc) affording **1b** as a clear oil (1.1 g, 33 %).^{1}H NMR (599 MHz, $CDCl_3$) δ 7.90 – 7.84 (m, 2H), 7.80 – 7.73 (m, 1H), 7.70 – 7.62 (m, 2H), 7.60 – 7.52 (m, 1H), 7.51 – 7.41 (m, 5H), 7.38 (s, 1H), 7.13 – 7.04 (m, 3H), 5.16 (s, 2H); ^{13}C NMR (151 MHz, $CDCl_3$) δ 196.8, 156.4, 138.4, 133.8, 133.1, 132.9, 132.8, 132.1, 129.9, 129.8, 129.5, 128.4, 128.0, 127.9, 127.6, 126.1, 125.9, 125.4, 124.5, 121.1, 112.8, 70.2. AMM (ESI) *m/z* calcd for $C_{24}H_{19}O_2^+$ $[M+H]^+$ 339.1380, found 339.1381.

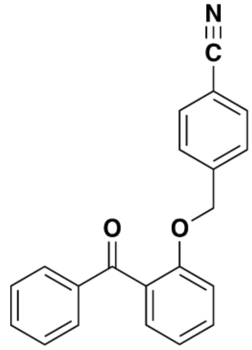

4-((2-benzoylphenoxy)methyl)benzonitrile (**1c**) was synthesized according to general procedure A using 2- hydroxybenzophenone (2 g, 10 mmol), 4-((2-benzoylphenoxy)methyl)benzonitrile (2.3 g, 12 mmol), Cs_2CO_3 (9.6 g, 30 mmol) and CH_3CN 15 mL). The crude product was purified by flash column chromatography (85:15, Hexanes:EtOAc) affording **1c** as a yellow oil (2 g, 64 %). Proton NMR data matched previously reported literature values.[5] ^{1}H NMR (400 MHz, $CDCl_3$) δ 7.84 (d, *J* = 6.9 Hz, 2H), 7.59 (t, *J* = 7.5 Hz, 1H), 7.56 – 7.42 (m, 6H), 7.15 (t, *J* = 7.5 Hz, 1H), 7.08 (d, *J* = 8.1 Hz, 2H), 7.02 (d, *J* = 8.7 Hz, 1H), 5.08 (s, 1H).

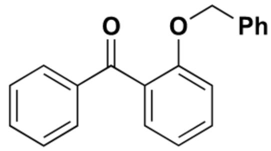

(2-(benzyloxy)phenyl)(phenyl)methanone (**1d**) was synthesized according to general procedure A using 2- hydroxybenzophenone (500 mg, 2.5 mmol), (bromomethyl)cyclopropane (512 mg, 3.0 mmol), Cs_2CO_3 (2.6 g, 7.5 mmol) and CH_3CN 15 mL). The crude product was purified by flash column chromatography (95:5, Hexanes:EtOAc) affording **1d** as a clear oil (700 mg, 97 %). Proton NMR data matched previously reported literature values.[5] ^{1}H NMR (400 MHz, $CDCl_3$) δ 7.84 (d, *J* = 8.4 Hz, 2H), 7.58 (t, *J* = 7.3 Hz, 1H), 7.51 – 7.42 (m, 4H), 7.26 – 7.18 (m, 3H), 7.10 (t, *J* = 7.4 Hz, 1H), 7.05 (d, *J* = 8.7 Hz, 1H), 7.03 – 6.97 (m, 2H), 5.03 (s, 2H).

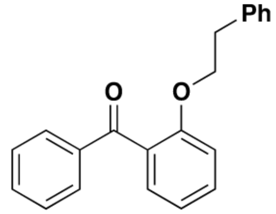

(2-phenethoxyphenyl)(phenyl)methanone (**1e**) was synthesized by following a modified literature procedure,[7] by preparing a suspension of NaH (0.229 g, 5 mmol, 4 equiv) in THF (0.25 M). 2-phenylethanol (0.3 mL, 2.5 mmol, 2 equiv) and 2-fluorobenzophenone (0.274 g, 1.25 mmol, 1 equiv) were then added and the mixture was heated to 60 ºC with stirring overnight. The reaction was then cooled to room temperature, quenched with sat. aq. ammonium chloride (5 mL), washed with brine (10 mL), dried over sodium sulfate, concentrated *in vacuo*, and purified by flash column chromatography (90:10 to 70:30 Hexanes: EtOAc) to afford **1e** (0.217 g, 52%) as a yellow oil. ^{1}H NMR (400 MHz, $CDCl_3$) δ 7.80 (d, *J* = 6.8 Hz, 2H), 7.56 (t, *J* = 7.4 Hz, 1H), 7.46 – 7.37 (m, 4H), 7.18 – 7.12 (m, 3H), 7.03 (t, *J* = 7.4 Hz, 1H), 6.99 – 6.90 (m, 3H), 4.08 (t, *J* = 6.9 Hz, 2H), 2.71 (t, *J* = 6.9 Hz, 2H); ^{13}C NMR (100 MHz, $CDCl_3$) δ 196.9, 156.8, 138.4, 138.1, 132.9, 132.1, 129.8, 129.8, 129.3, 129.0, 128.5, 128.3, 126.5, 120.9, 112.5, 69.5, 35.6; IR (neat) 3061, 3028, 2928 ,2873, 1663, 1598, 1450, 1315, 1295, 1240 cm^{-1}; AMM (ESI) *m/z* calcd $C_{21}H_{19}O_2^+$ $[M+H]^+$ 303.1380, found 303.1382.

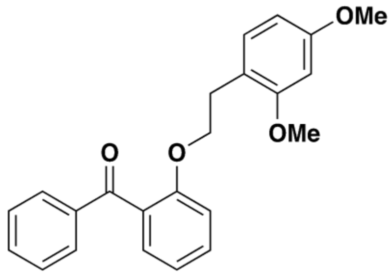

(2-(3,4-dimethoxyphenethoxy)phenyl)(phenyl)methanone (**1f**) was synthesized by following a modified literature procedure,[7] by preparing a suspension of NaH (0.275 g, 5 mmol, 4 equiv) in THF (0.25 M). 2-(3,4-dimethoxy)phenylethanol (0.472 g mL, 2.5 mmol, 2 equiv) and 2-fluorobenzophenone (0.277 g, 1.25 mmol, 1 equiv) were then added and the mixture was heated to 60 ºC with stirring overnight. The reaction was then cooled to room temperature, quenched with sat. aq. ammonium chloride (5 mL), washed with brine (10 mL), dried over sodium sulfate, concentrated *in vacuo*, and purified by flash column chromatography (90:10 to 70:30, Hexanes:EtOAc) to afford **1f** (0.217 g, 52%) as a yellow oil. ^{1}H NMR (400 MHz, $CDCl_3$) δ 7.79 (d, *J* = 6.9 Hz, 2H), 7.55 (t, *J* = 7.4 Hz, 1H), 7.45 – 7.36 (m, 4H), 7.03 (t, *J* = 7.5 Hz, 1H), 6.94 (d, *J* = 8.4 Hz, 1H), 6.67 (d, *J* = 8.0 Hz, 1H), 6.58 (d, *J* = 1.9 Hz, 1H), 6.53 (dd, *J* = 8.0, 2.1 Hz, 1H), 4.08 (t, *J* = 6.8 Hz, 2H), 3.82 (s, 3H), 3.78 (s, 3H), 2.68 (t, *J* = 6.8 Hz, 2H); ^{13}C NMR (101 MHz, $CDCl_3$) δ 196.7, 156.8, 148.8, 147.7, 138.3, 132.9, 132.1, 130.8, 129.8, 129.2, 128.3, 120.9, 120.8, 112.6, 112.4, 111.2, 69.7, 56.0, 55.9, 35.2; IR (neat) 2935, 2834, 1663, 1597, 1259, 1234 cm^{-1}; AMM (ESI) *m/z* calcd $C_{23}H_{23}O_4^+$ $[M+H]^+$ 363.1591, found 363.1595.

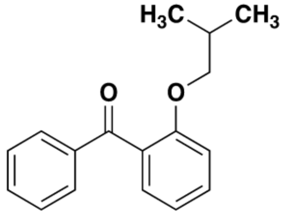

(2-isobutoxyphenyl)(phenyl)methanone (**1g**) was synthesized according to general procedure A using 2- hydroxybenzophenone (2 g, 10 mmol), 1-bromo-2-methylpropane (1.6 g, 12 mmol), Cs_2CO_3 (9.6 mg, 30 mmol) and CH3CN 15 mL). The crude product was purified by flash column chromatography (97:3, Hexanes:EtOAc) affording **1g** as a clear oil (260 mg, 9 %).Proton NMR data matched previously reported literature values.[5] ^{1}H NMR (400 MHz, $CDCl_3$) δ 7.85 – 7.72 (m, 2H), 7.55 (t, *J* = 7.4 Hz, 1H), 7.50 – 7.36 (m, 4H), 7.06 (t, *J* = 7.4 Hz, 1H), 6.96 (d, *J* = 8.3 Hz, 1H), 3.66 (d, *J* = 6.2 Hz, 2H), 1.84 – 1.65 (m, 1H), 0.68 (d, *J* = 6.7 Hz, 6H).

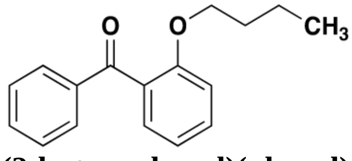

(2-butoxyphenyl)(phenyl)methanone (**1h**) was synthesized according to general procedure A using 2- hydroxybenzophenone (2 g, 10 mmol), 1-bromobutane (1.4 g, 12 mmol), Cs_2CO_3 (9 g, 30 mmol) and CH3CN 15 mL). The crude product was purified by flash column chromatography (90:10, Hexanes:EtOAc) affording **1h** as a yellow oil (1.2 g, 47 %). Proton NMR data matched previously reported literature values.[5] ^{1}H NMR (400 MHz, $CDCl_3$) δ 7.84 – 7.76 (m, 2H), 7.59 – 7.52 (m, 1H), 7.50 – 7.40 (m, 4H), 7.14 – 7.02 (m, 1H), 6.97 (d, *J* = 8.3 Hz, 1H), 3.89 (t, *J* = 6.3 Hz, 1H), 1.43 (p, *J* = 6.3 Hz, 2H), 1.07 (h, *J* = 7.4 Hz, 2H), 0.76 (t, *J* = 7.4 Hz, 3H).

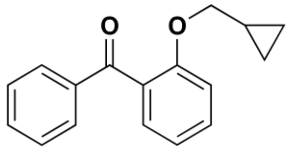

(2-(cyclopropylmethoxy)phenyl)(phenyl)methanone (**1i**) was synthesized according to general procedure A using 2- hydroxybenzophenone 500 mg, 2.5 mmol), (bromomethyl)cyclopropane (416 mg, 3.0 mmol), Cs_2CO_3 (2 g, 7.5 mmol) and CH_3CN 20 mL). The crude product was purified by flash column chromatography (40:60 CH_2Cl_2:Hexanes) affording **1i** as a yellow oil (400 mg, 63 %).^{1}H NMR (599 MHz, $CDCl_3$) δ 7.80 (d, J = 7.9 Hz, 2H), 7.62 – 7.49 (m, 1H), 7.49 – 7.34 (m, 4H), 7.10 – 7.00 (m, 1H), 6.94 (d, J = 8.3 Hz, 1H), 3.75 (d, J = 6.6 Hz, 2H), 0.97 – 0.76 (m, 1H), 0.34 (d, J = 7.9 Hz, 2H), 0.11 – -0.12 (m, 2H); ^{13}C NMR (151 MHz, $CDCl_3$) δ 196.9, 156.8, 138.4, 132.6, 132.0, 129.8, 129.6, 129.3, 128.1, 120.7, 112.8, 72.7, 9.8, 2.7.; AMM (ESI) *m/z* calcd for $C_{17}H_{17}O_2^+$ [M+H]$^+$ 253.1223, found 253.12231.

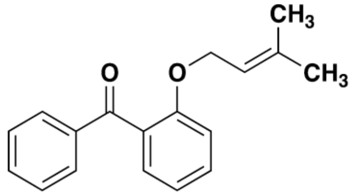

(2-((3-methylbut-2-en-1-yl)oxy)phenyl)(phenyl)methanone (**1j**) was synthesized according to general procedure A using 2- hydroxybenzophenone (2 g, 10 mmol), 1-bromo-3-methylbut-2-ene (2 g, 12 mmol), Cs_2CO_3 (9 g, 30 mmol) and CH3CN 15 mL). The crude product was purified by flash column chromatography (60:40 CH_2Cl_2:Hexanes) affording **1j** as a clear oil (450 mg, 17 %). Proton NMR data matched reported literature values.[5] ^{1}H NMR (400 MHz, $CDCl_3$) δ 7.79 (d, J = 7.0 Hz, 2H), 7.54 (t, J = 7.4 Hz, 1H), 7.48 – 7.33 (m, 4H), 7.03 (t, J = 7.4 Hz, 1H), 6.98 (d, J = 8.3 Hz, 1H), 5.11 (t, J = 6.6 Hz, 1H), 4.45 (d, J = 6.5 Hz, 2H), 1.65 (s, 3H), 1.57 (s, 3H).

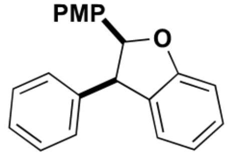

(2*S*,3*R*)-2-(4-methoxyphenyl)-3-phenyl-2,3-dihydrobenzofuran (**3a**) was synthesized by general procedure C using the requisite hydrazone (15 mg, 0.045 mmol), MnO_2 (31 mg, 0.36 mmol), and dirhodium catalyst (1 mol%) in CH_3CN. Proton NMR data matched previously reported literature values.[5] ^{1}H NMR (400 MHz, $CDCl_3$) δ 7.30 – 7.23 (m, 1H), 7.10 (d, J = 7.4 Hz, 1H), 7.05 – 7.00 (m, 4H), 6.97 – 6.88 (m, 3H), 6.77 – 6.64 (m, 2H), 6.60 (d, J = 8.5 Hz, 2H), 5.97 (d, J = 8.8 Hz, 1H), 4.82 (d, J = 8.8 Hz, 1H), 3.68 (s, 3H).

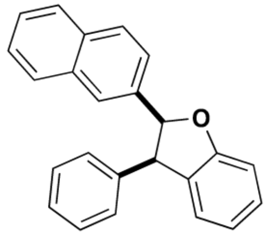

(2*S*,3*R*)-2-(naphthalen-2-yl)-3-phenyl-2,3-dihydrobenzofuran (**3b**) was synthesized by general procedure C using the requisite hydrazone (20 mg, 0.056 mmol), MnO_2 (39.4 mg, 0.45 mmol), and Rh_2(*R*-PTAD)$_4$ (1 mol%) in CH_3CN (isolated as a mixture of diastereomers). The crude product was purified by flash column chromatography (80:20, hexanes:CH_2Cl_2) affording **3b** as a

white solid (16 mg, 88%, 95:5 dr). ^{1}H NMR (major) (400 MHz, CD_2Cl_2) δ 7.71 – 7.65 (m, 2H), 7.62 (s, 1H), 7.50 (d, *J* = 8.5 Hz, 1H), 7.42 – 7.35 (m, 2H), 7.30 (td, *J* = 7.7, 1.4 Hz, 1H), 7.17 – 7.05 (m, 2H), 7.02 (dd, *J* = 8.5, 1.8 Hz, 1H), 6.99 – 6.87 (m, 4H), 6.77 – 6.63 (m, 2H), 6.18 (d, *J* = 8.9 Hz, 1H), 4.95 (d, *J* = 8.9 Hz, 1H).; ^{13}C NMR (major) (151 MHz, $CDCl_3$) δ 160.5, 139.4 135.4, 133.1, 132.9, 131.0, 129.5, 129.2, 128.2, 128.1, 127.9, 127.5, 127.0, 126.4, 126.1, 126.0, 125.9, 124.8, 121.8, 110.2, 89.1, 54.2.; AMM (ESI) *m/z* calcd for $C_{24}H_{19}O^+$ 323.1430, found 323.1431.

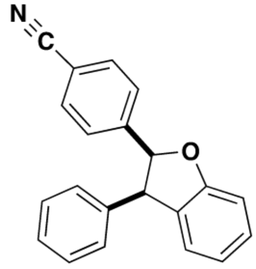

4-((2*S*,3*R*)-3-phenyl-2,3-dihydrobenzofuran-2-yl)benzonitrile (**3c**) was synthesized by general procedure C using the requisite hydrazone (20 mg, 0.061mmol), MnO_2 (42.5 mg, 0.49 mmol), and dirhodium catalyst (1 mol%) in CH_3CN. Proton NMR data matched previously reported literature values.[5] ^{1}H NMR (400 MHz, $CDCl_3$) δ 7.38 (d, *J* = 8.4 Hz, 2H), 7.31 (t, *J* = 8.2 Hz, 1H), 7.17 (d, *J* = 8.5 Hz, 2H), 7.14 – 7.01 (m, 5H), 6.99 (t, *J* = 7.4 Hz, 1H), 6.71 – 6.65 (m, 2H), 6.07 (d, *J* = 9.1 Hz, 2H), 4.97 (d, *J* = 9.1 Hz, 2H).

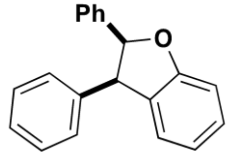

(2*S*,3*R*)-2,3-diphenyl-2,3-dihydrobenzofuran (**3d**) was synthesized by general procedure C using the requisite hydrazone (25 mg, 0.066 mmol), MnO_2 (46 mg, 0.57 mmol), and dirhodium catalyst (1 mol%) in CH_3CN. Proton NMR data matched previously reported literature values.[5] ^{1}H NMR (400 MHz, $CDCl_3$) δ 7.35 – 7.26 (m, 1H), 7.18 – 7.00 (m, 10H), 7.00 – 6.93 (m, 1H), 6.79 – 6.66 (m, 2H), 6.06 (d, *J* = 8.9 Hz, 1H), 4.91 (d, *J* = 8.9 Hz, 1H).

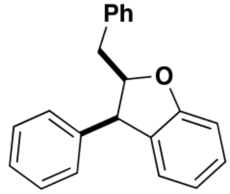

(2*R*,3*R*)-2-benzyl-3-phenyl-2,3-dihydrobenzofuran (**3e**) was synthesized by following a modified literature procedure,[8] by preparing a solution of the requisite hydrazone (0.050 g, 0.156 mmol, 1 equiv) in DCM (0.015 M) and adding manganese dioxide (0.112 g, 1.264 mmol, 8 equiv). This heterogenous mixture was allowed to stir at room temperature for 4 hours before cooling to 0 ºC. Rh_2(*S*-BTPCP)$_4$ (**C12**) (0.002 g, 1.6 µmol, 1 mol %) was then added and the mixture was allowed to stir to room temperature overnight. The reaction was then filtered through celite, concentrated *in vacuo*, and purified by flash column chromatography (95:5 to 90:10, Hexanes:EtOAc) to afford **3e** (0.039 g, 86%) as a crystalline white solid. ^{1}H NMR (400 MHz, $CDCl_3$) δ 7.29 – 7.22 (m, 5H), 7.21 – 7.15 (m, 2H), 7.11 – 7.04 (m, 3H), 7.04 – 6.99 (m, 2H), 6.91 – 6.84 (m, 2H), 5.15 (td, *J* = 9.1, 4.9 Hz, 1H), 4.57 (d, *J* = 8.3 Hz, 1H), 2.71 (dd, *J* = 14.6, 9.1 Hz, 1H), 2.52 (dd, *J* = 14.6, 4.7 Hz, 1H); ^{13}C NMR (100 MHz, $CDCl_3$) δ 159.8, 139.6, 138.4, 131.5,

129.4, 129.2, 128.8, 128.5, 128.4, 127.3, 126.5, 125.8, 121.1, 110.1, 87.6, 51.8, 38.0; IR (neat) 3029, 2922, 1478, 1453, 1231, 728 cm^{-1}.

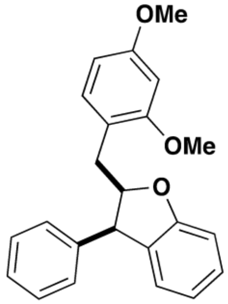

(2*R*,3*R*)-2-(3,4-dimethoxybenzyl)-3-phenyl-2,3-dihydrobenzofuran (**3f**) was synthesized by following a modified literature procedure,[8] by preparing a solution of the requite hydrazone (0.050 g, 0.133 mmol, 1 equiv) in DCM (0.015 M) and adding manganese dioxide (0.095 g, 1.063 mmol, 8 equiv). This heterogenous mixture was allowed to stir at room temperature for 4 hours before cooling to 0 ºC. Rh_2(*S*-BTPCP)$_4$ (0.002 g, 1.33 µmol, 1 mol %) was then added and the mixture was allowed to stir to room temperature overnight. The reaction was then filtered through celite, concentrated *in vacuo*, and purified by flash column chromatography (95:5 to 90:10, Hexanes:EtOAc) to afford **3f** (0.039 g, 86%) as a crystalline white solid. ^{1}H NMR (300 MHz, $CDCl_3$) δ 7.30 – 7.23 (m, 3H), 7.18 (t, *J* = 7.7 Hz, 1H), 7.08 – 6.98 (m, 3H), 6.93 – 6.84 (m, 2H), 6.77 (d, *J* = 8.3 Hz, 1H), 6.66 (dd, *J* = 8.2, 2.1 Hz, 1H), 6.55 (d, *J* = 2.1 Hz, 1H), 5.13 (td, *J* = 8.7, 5.2 Hz, 1H), 4.55 (d, *J* = 8.3 Hz, 1H), 3.85 (s, 3H), 3.81 (s, 3H), 2.69 (dd, *J* = 14.5, 8.8 Hz, 1H), 2.49 (dd, *J* = 14.5, 5.2 Hz, 1H).; ^{13}C NMR (76 MHz, $CDCl_3$) δ 159.7, 148.7, 147.6, 139.6, 131.6, 130.8, 129.4, 128.7, 128.4, 127.2, 125.8, 121.1, 121.0, 112.6, 111.2, 110.0, 87.7, 56.0, 55.8, 51.6, 37.4; IR (neat) 2932, 2834, 1666, 1515, 1479, 1461, 1261, 1231, 1141, 1028 cm^{-1}; AMM (ESI) *m/z* calcd $C_{23}H_{23}O_3^+$ $[M+H]^+$ 347.1642, found 347.1643.

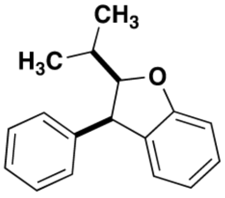

(2*R*,3*R*)-2-isopropyl-3-phenyl-2,3-dihydrobenzofuran (**3g**) was synthesized by general procedure C using the requsite hydrazone (20 mg, 0.075 mmol), MnO_2 (52 mg, 0.6 mmol), and dirhodium catalyst (1 mol%) in CH_3CN. Proton NMR data matched previously reported literature values.[5] ^{1}H NMR (400 MHz, $CDCl_3$) δ 7.25 (t, *J* = 7.5 Hz, 2H), 7.24 – 7.16 (m, 2H), 7.09 – 7.02 (m, 3H), 6.94 (d, *J* = 8.0 Hz, 1H), 6.87 – 6.82 (m, 1H), 4.42 – 4.32 (m, 2H), 1.86 – 1.68 (m, 1H), 1.11 (d, *J* = 6.5 Hz, 3H), 0.78 (d, *J* = 6.5 Hz, 3H).

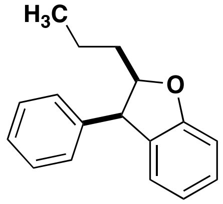

(2*R*,3*R*)-2-propyl-3-phenyl-2,3-dihydrobenzofuran (**3h**) *The diastereomers of compound **3h** proved difficult to separate. As such we did not include NMR data however it has been isolated in our previous work as a mixture of diastereomers.[5,6]

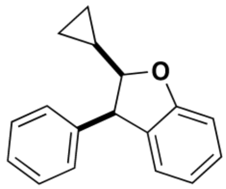

(2*R*,3*R*)-2-cyclopropyl-3-phenyl-2,3-dihydrobenzofuran (**3i**) was synthesized by general procedure C using the requsite hydrazone (25 mg, 0.075 mmol), MnO_2 (52 mg, 0.60 mmol), and and Rh_2(*R*-PTAD)$_4$ (1 mol%) in CH_3CN. The crude product was purified by flash column chromatography (50:50, CH_2Cl_2:Hexanes) affording **3i** as a clear oil (16 mg, 97%, >95:5 dr). ^{1}H NMR (599 MHz, cdcl$_3$) δ 7.30 – 7.24 (m, 2H), 7.24 – 7.17 (m, 2H), 7.08 (d, *J* = 7.4 Hz, 1H), 7.04 (d, *J* = 7.3 Hz, 2H), 6.96 – 6.84 (m, 2H), 4.58 (d, *J* = 8.5 Hz, 1H), 4.11 (t, *J* = 8.6 Hz, 1H), 0.61 – 0.51 (m,21H), 0.51 – 0.46 (m, 1H), 0.36 – 0.24 (m, 1H), 0.24 – 0.14 (m, 1H). ^{13}C NMR (151 MHz, cdcl$_3$) δ 159.88, 140.27, 131.11, 129.16, 128.68, 128.14, 126.81, 125.80, 120.94, 109.65, 92.80, 51.51, 11.68, 4.19, 2.39; AMM (ESI) *m/z* calcd for $C_{17}H_{17}O^+$ 237.1274, found 237.1283.

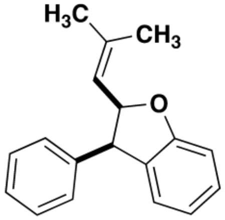

(2*R*,3*R*)-2-(2-methylprop-1-en-1-yl)-3-phenyl-2,3-dihydrobenzofuran (**3j**) was synthesized by general procedure C using the requisite hydrazone (15 mg, 0.053 mmol), MnO_2 (37 mg, 0.42 mmol), and dirhodium catalyst (1 mol%) in CH_3CN. Proton NMR data matched previously reported literature values.[5] ^{1}H NMR (400 MHz, $CDCl_3$) δ 7.29 – 7.16 (m, 4H), 7.08 (d, *J* = 7.3 Hz, 1H), 6.98 – 6.81 (m, 4H), 5.61 (t, *J* = 8.9 Hz, 1H), 4.89 (d, *J* = 9.3 Hz, 1H), 4.54 (d, *J* = 8.5 Hz, 1H), 1.70 (s, 3H), 1.56 (s, 3H).

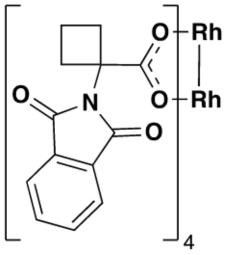

C14 was synthesized according to general procedure D 500 mg of the requsite ligand (2.00 mmol, 8 eq), 110 mg (0.25 mmol, 1 eq) of $Rh_2(OAc)_4$ (**C0**) and 20 ml chlorobenzene (0.1M). The product (200 mg 67%) was isolated by flash chromatography (3:97 MeOH:CH_2Cl_2) as a green solid. ^{1}H NMR (400 MHz, $CDCl_3$) δ 7.75 (dd, *J* = 5.5, 3.0 Hz, 2H), 7.65 (dd, *J* = 5.5, 3.1 Hz), 1.78 – 1.64 (m, 5.78H). AMM (ESI) *m/z* for $C_{52}H_{40}N_4O_{16}Rh_2^+$, calcd, 1183.0628., found, 1183.0536. * *This compound has a population of bound and unbound ethyl acetate.*

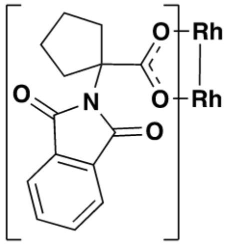

C15 was synthesized according to general procedure D 470 mg of the requisite ligand (1.81 mmol 8 eq), 110 mg (0.25 mmol, 1 eq) of $Rh_2(OAc)_4$ (**C0**) and 20 ml chlorobenzene (0 .1M). The product (250 mg 80%) was isolated by flash chromatography (3:97 MeOH CH_2Cl_2) as a green solid. ^{1}H NMR (400 MHz, $CDCl_3$) δ 7.76 – 7.69 (m, 2H), 7.68 – 7.63 (m, 2H), 2.72- 2.64 (m, 4H), 2.21-2.07 (m, 1H), 1.89 – 1.77 (m, 5H)* *This signal is obscured by a singlet we believe is due to a population of water bound to the metal center accounting for inaccurate integration. This observation is consistent with our crystallographic data.* AMM (ESI) *m/z* for $C_{54}H_{44}N_4O_{16}Rh_2^+$, calcd, 1239.1254., found 1239.1182.

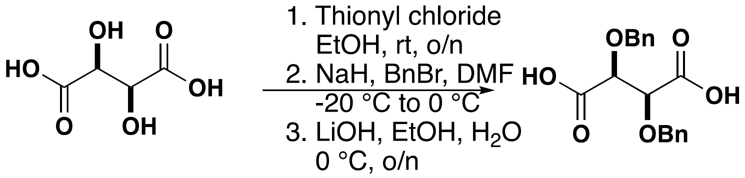

(2*S*,3*S*)-2,3-bis(benzyloxy)succinic acid was synthesized by the literature procedures.[32]

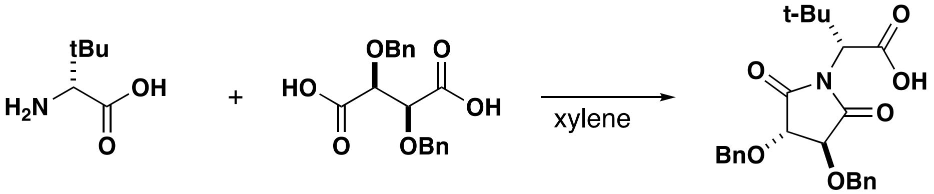

***tert*-butyl (*R*)-2-((3*S*,4*S*)-3,4-bis(benzyloxy)-2,5-dioxopyrrolidin-1-yl)-3,3-dimethylbutanoate** (**L10**) was synthesized by the modified literature procedure (## J.-L. Zheng et al. / Tetrahedron: Asymmetry 22 (2011) 257–263).[33] To a non-dried round bottom flask containing dicarboxylic acid ## (1.7000g, 5.1754 mmol, 1 equiv.), amino acid ## (1.1627 g, 6.2082 mmol, 1.2 equiv.) and *o*-xylene (125 mL) were added. A dean-stark trap and a reflux condenser were then connected to the reaction flask. The reaction heated it up to 165~175 °C until dripping. The dean-stark trap was repeatedly drained until the solvent in the reaction flask was around 10~20 mL. The reaction was cooled down to the rt and excess ethyl acetate was added into the reaction flask, following by extraction with 3M aqueous HCl (3x) and brine (1x). The organic layer was combined, dried over anhydrous Na_2SO_4, filtered, and solvent was removed by rotary evaporation. The crude product was purified by flash column chromatography (20:80 EtOAc:Hexanes) affording a yellow oil (1.812 g, 4.2587 mmol, 82 %). ^{1}H NMR (400 MHz, $CDCl_3$) δ 7.40 – 7.33 (m, 10H), 4.99 (d, *J* = 11.6 Hz, 2H), 4.77 (d, *J* = 11.6 Hz, 2H), 4.52 (s, 1H), 4.46 (s, 2H), 1.12 (s, 9H). ^{13}C NMR (76 MHz, $CDCl_3$) δ 172.5, 172.2, 136.6, 128.7, 128.4, 128.4, 78.6, 73.6, 60.1, 35.6, 28.0. AMM (ESI) *m/z* calcd for C24H28NO6+ $[M+H]^+$ 426.1911, found 426.1910. $[\alpha]D^{26.1}$ = -0.80 (CH_2Cl_2)

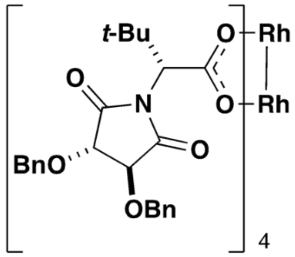

(**C11**) was synthesized by general procedure D using $Rh_2(OAc)_4$ (75.0 mg, 0.170 mmol), ligand L10 (577.6 mg, 1.357 mmol, 8 equiv.), in chlorobenzene (20.0 mL, 0.00845 M). The crude product was purified by flash column chromatography (15:85 EtOAc:Hexanes) affording a green solid (260 mg, 80%). ^{1}H NMR (400 MHz, $CDCl_3$) δ 7.40 – 7.33 (m, 10H), 4.99 (d, *J* = 11.6 Hz, 2H), 4.77 (d, *J* = 11.6 Hz, 2H), 4.52 (s, 1H), 4.46 (s, 2H), 1.12 (s, 9H^{13}C NMR (76 MHz, $CDCl_3$) δ 172.51, 172.23, 136.55, 128.68, 128.41, 128.37, 78.62, 73.56, 60.08, 35.57, 28.00. AMM (ESI) *m/z* calcd for C96H105N4O24Rh2+ 1903.5223, found 1903.5219. [α]D$^{25.4}$ = -1.4 (CH_2Cl_2)

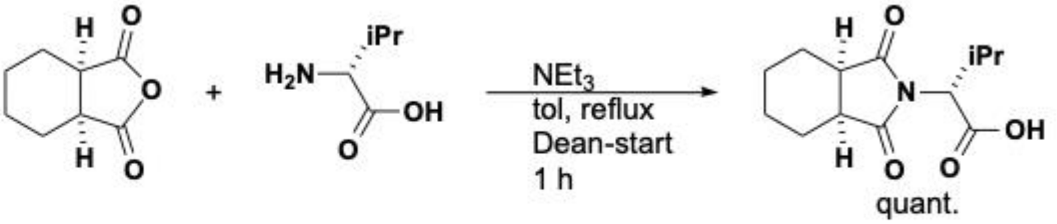

(*R*)-2-((3a*R*,7a*S*)-1,3-dioxooctahydro-2*H*-isoindol-2-yl)-3-methylbutanoic acid (L11)

In a 50 mL flask with a Dean stark trap and reflux condenser were placed D-Valine (1.1751g, 10.000 mmol), anhydride (1.5416g, 10.000 mmol), toluene (30 mL, 0.318M), and triethylamine (370.5 mg, 1.000 mmol). The flask was heated to 140 °C until dripping. The reaction was allowed to stir at 140 °C for 1 hour. After cooling down to room temperature, excess ethyl acetate was added to the reaction mixture, and then wash with 3M HCl (three times), and brine (1 time). The organic layer was collected, dried over Na2SO4, filtered, and concentrated in vacuo to afford a white solid (quant.). The obtained white solids were used in the next step without further purification. H NMR(400 MHz, $CDCl_3$) δ 9.51 (s, 1H), 4.42 (d, *J* = 8.6 Hz, 1H), 2.99 – 2.86 (m, 2H), 2.73 – 2.56 (m, 1H), 1.96 – 1.70 (m, 4H), 1.55 – 1.36 (m, 4H), 1.13 (d, *J* = 6.7 Hz, 3H), 0.86 (d, *J* = 6.8 Hz, 3H); C NMR (101 MHz, $CDCl_3$) δ 179.2, 179.2, 173.8, 57.5, 39.8, 39.6, 27.8, 23.9, 23.6, 21.8(2C), 21.1, 19.4. AMM (ESI) *m/z* calcd for C13H20NO4+ 254.1387, found 254.1389. [α]D$^{25.5}$ = 0.41 (CH_2Cl_2)

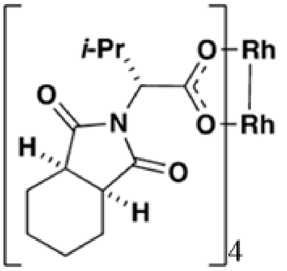

(C10) was synthesized by general procedure D using Rh2(OAc)4 (100.0 mg, 0.2262 mmol), Ligand L10 (458.5 mg, 1.810 mmol, 8 equiv.), in chlorobenzene (20.0 mL, 0.0113 M). The crude product was purified by flash column chromatography affording a green solid (250 mg, 91%). 1H NMR (300 MHz, $CDCl_3$) δ 4.40 (d, *J* = 8.3 Hz, 4H), 2.98 – 2.70 (m, 8H), 2.61 – 2.43 (m, 4H), 1.94 – 1.65 (m, 16H), 1.50 – 1.23 (m, 16H), 1.04 (d, *J* = 6.6 Hz, 12H), 0.72 (d, *J* = 6.7 Hz, 12H); 13C NMR (76 MHz, $CDCl_3$) δ 187.7, 178.7, 58.9, 39.9, 39.2, 27.6, 23.9, 23.1, 21.6, 21.5, 21.2, 19.0.; AMM (ESI) *m/z* calcd for C52H73N4O16Rh2+ 1215.3126, found 1215.3160. [α]D$^{25.9}$ = -0.23 (CH_2Cl_2)

III. Computational Methods and Details

a. Dirhodium(II) Complexes

Generating Conformational Ensembles

Initial structures for each dirhodium catalyst were generated using reported X-ray crystal structures deposited in the Cambridge Structural Database (CSD). Catalysts for which no crystal structure was found were constructed from X-ray structures of the closest analogous crystal structure. Conformational searches were performed in the gas phase using the molecular mechanics force field OPLS3e[8] as employed in MacroModel.[9] Rh and carboxylate ligand atoms were frozen during the search procedure as described in a previous protocol.[10] Conformations found within 41.9 kJ/mol (10.01 kcal/mol) were saved for each search. To reduce the number of conformations calculated at higher levels of theory, the clustering tools implemented in MacroModel were used to remove redundant conformers by the root-mean-square deviation (RMSD) for all catalyst atomic positions. For catalysts that yielded more than 20 conformations, hierarchical clustering was performed using the average-linkage method and the Kelley Penalty score[11] was used to identify the optimum number of clusters. The conformer at the centroid of each cluster was considered as a representative of the configurations in the cluster. Conformers of catalysts yielding less than 20 conformations were selected by hand based on the degree of structural differences to represent the conformational ensemble.

Calculating Representative Conformers by DFT

Representative conformers were geometrically optimized in the gas phase at the B3LYP level of theory using Grimme's dispersion correction with Becke-Johnson damping (BJD3) and pure gaussian functions for *d*-orbitals (6D). The LANL2DZ basis set with corresponding Hay-Wadt effective core potential was applied to Rh, while the 6-31G** basis set was applied to all other atoms. Frequency calculations were performed to ensure that optimized geometries had converged to a ground state. Single point energies were calculated at the M06 level of theory using Grimme's dispersion correction with Becke-Johnson damping (BJD3). The SDD basis set with corresponding Hay-Wadt effective core potential was applied to Rh, while the Def2TZVP basis set was applied to all other atoms. These calculations were performed using the solvation model-based density (SMD) method in dichloromethane to represent the experimental environment more accurately (See references [12–17] for computational methods). Relative free energies utilize the solvated electronic energies to correct the gas phase free energies. Only conformers within 3 kcal/mol of the relative minimum free energy for each catalyst ensemble were considered further. Natural bond orbital (NBO) analysis[18] was conducted to obtain catalyst electronic descriptors, including Rh *d*-orbital energies.

Assessing Rh-Carbene Conformer Stability

While C4 "chiral crowns" are regarded to induce the highest selectivity,[19] the catalytic competency of lower symmetry conformations has been shown to be influential to the success of select asymmetric applications.[20,21] To assess the relevance of multiple conformations to the cyclization of 2-alkoxybenzophenones, two catalyst conformational ensembles were computed with a docked carbene intermediate structure derived from **2a** at the DFT level described previously (Figure S1). The Boltzmann populations for the lowest energy conformations indicate that for catalyst **C16** the

most populated states are of lower symmetry. As these conformations are significantly low in energy compared to the C4 analogs, the catalytic involvment of multiple conformations cannot be ruled out.

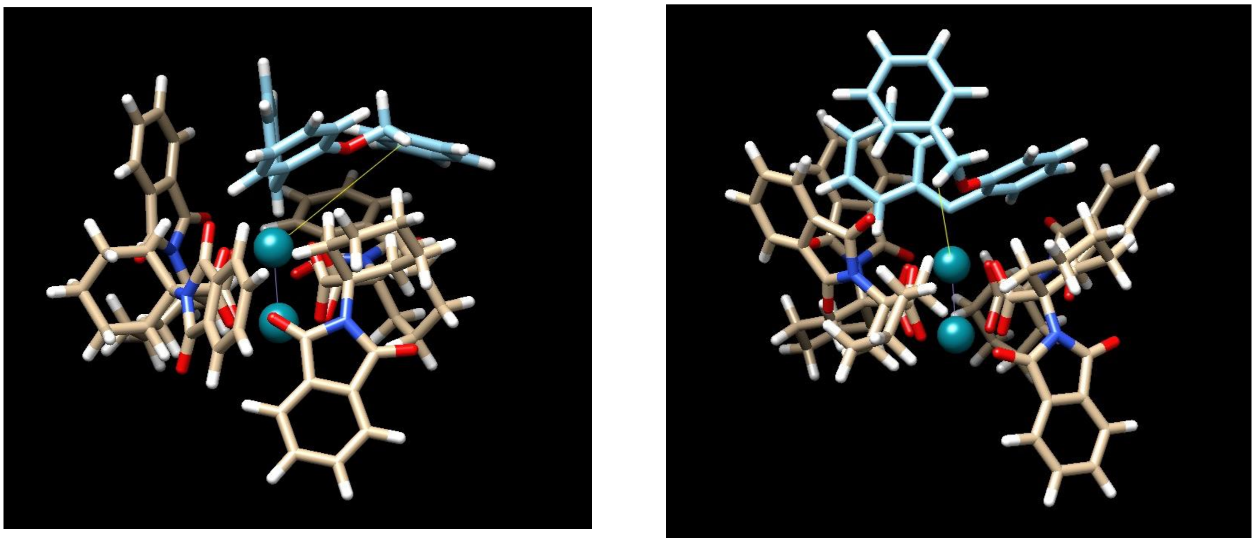

C16 with carbene (**2a**)
~D2 (34% relative population)

C16 with carbene (**2a**)
~3-1 (53% relative population)

Figure S1. Lowest energy Rh-carbene conformers for **C16** and **2a**. The yellow line indicates the distance of the insertion center to the binding Rh.

Automated Catalyst Face Classifiers

Design of dirhodium paddlewheel complexes traditionally operates under the mechanistic assumption that high symmetry states afford privileged reactivity.[22] High symmetry states restrict the degrees of freedom possessed by a bound intermediate, decreasing the number of orientations accessible to the structure. Particularly, the C4 "chiral crown" conformation achieved by noncovalent interactions (NCI) between phthalimide groups is often assumed to be responsible for the highest observed selectivity.[20] Additionally, ligand-substrate NCIs have been observed. These interactions may contribute to the restriction of conformational freedom by electrostatically preferencing certain conformations accessible to the bound intermediate.

In the evaluation of the experimental matrix, catalysts for which no chiral crown conformation exists were still observed to yield highly *syn*-selective reactions, indicating need for a more generalized mechanistic understanding of the influence of catalyst effects on diastereoselectivity. Thus, a broader assumption was constructed by the inference that *high diastereoselectivity is promoted by high steric hindrance at the reactive catalyst pocket*. Through this enhanced hypothesis, a two-fold approach to catalyst face classification was employed to determine which face of each conformer would be parametrized for modeling *syn*-selectivity.

First Classifier (Ligand NCI Steric Constraint)

The first classifier was applied to catalysts with π-conjugated ligands by identifying the face most capable of forming ligand-substrate and ligand-ligand NCIs. A Python script was developed to identify the catalyst face encompassed by the most atoms capable of forming NCIs. To do this, the script identifies ligand atoms of interest by type (i.e. oxygen, nitrogen, aromatic carbon, etc.) and by element and number of covalent bonds. Atoms of interest for forming NCIs were identified by element and hybridization (sp^2 or sp). Each atom is then assigned “Rh1-axial”, “Rh2-axial”, or “equatorial” based on the azimuth angle of the atom relative to the zenith vector defined by the Rh2-Rh1 bond (Figure S2). Atoms with an azimuth angle >= 120° are defined as “Rh2-axial”, <= 60° as “Rh1-axial”, and between 60-120° as “equatorial”. The percentage of atoms in each category was calculated for all conformations.

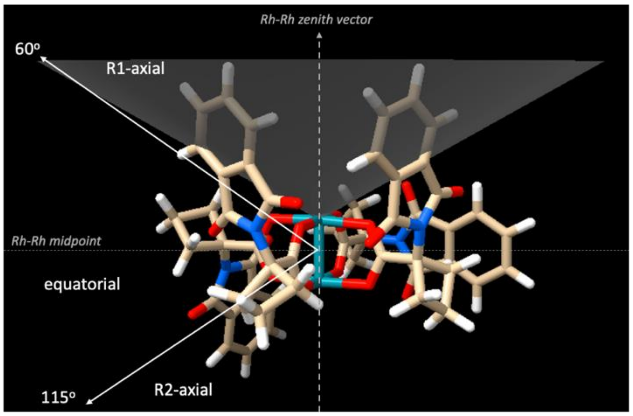

Figure S2. Visualization of the first classifier on a conformer of **C1**, Rh_2(*R*-PTAD)$_4$.

The classification threshold for differentiating Rh faces was a >=10% difference in the number of axial atoms. This threshold was determined based on analysis of the **C14** ensemble (Figure S3). Conformers with distinct C2 symmetry were determined to have up to 10% difference in axial atoms. Conformations with less than 10% difference are considered sterically equivalent and parameters from each face are averaged. Classification of conformers with a difference close to the threshold were checked by hand to ensure proper classification.

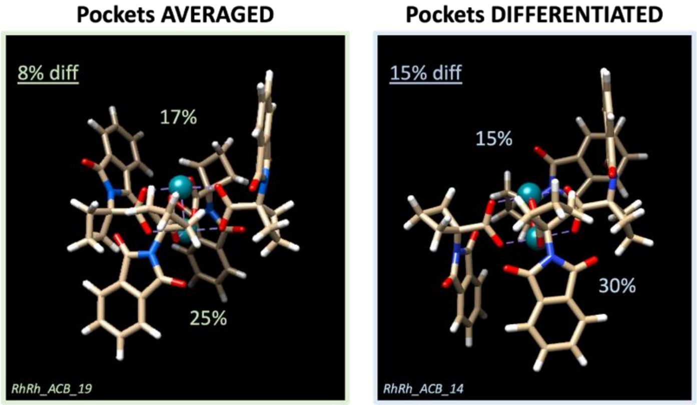

Figure S3. Differentiation between **C14** conformers with averaged pockets (<10% difference) and sterically differentiable pockets (>10% difference).

Second Classifier (General Steric Constraint)

The second classifier was implemented to analyze the difference in steric properties of each dirhodium pocket for ligands both with and without π-conjugated systems. To determine which face would be parameterized for modeling, a steric surrogate was constructed from the reported *syn*-transition state **TS-1a**.[6] Using a Python script, the surrogate was docked 2.0 Å from each Rh and the number of overlaps between ligand and surrogate atoms using Van der Waals radii was counted (Figure S4). To account for multiple bonding alignments within the pocket, the surrogate was rotated about the C-Rh bond by 5°. The number of orientations of the surrogate resulting in no steric overlaps between ligands and surrogate was summed for each face and analyzed as a percentage of orientations.

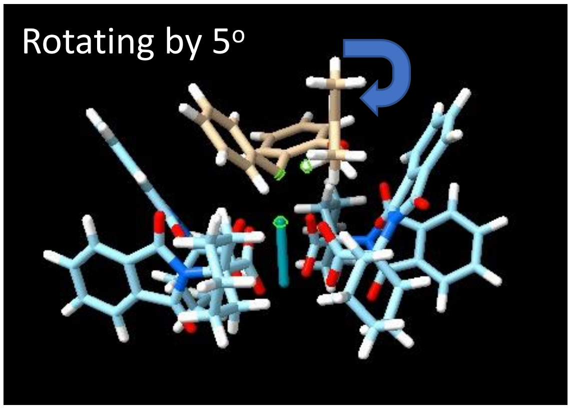

Figure S4. Depiction of the second classifier on **C16**.

Results of the second classifier were also used to check the classifications of catalysts with π-conjugated ligands, ensuring that the more hindered face (identified by the first classifier) can encompass the steric demands of cyclization (identified by the second classifier). If the catalyst faces could contain the same number of overlap-free orientations within a 10% difference, the 1st classification holds. However, if 1% of orientations was not achieved by the identified face, the face was regarded as too hindered to be relevant to the stereoselective step, and the opposite face was used instead. (Figure S5).

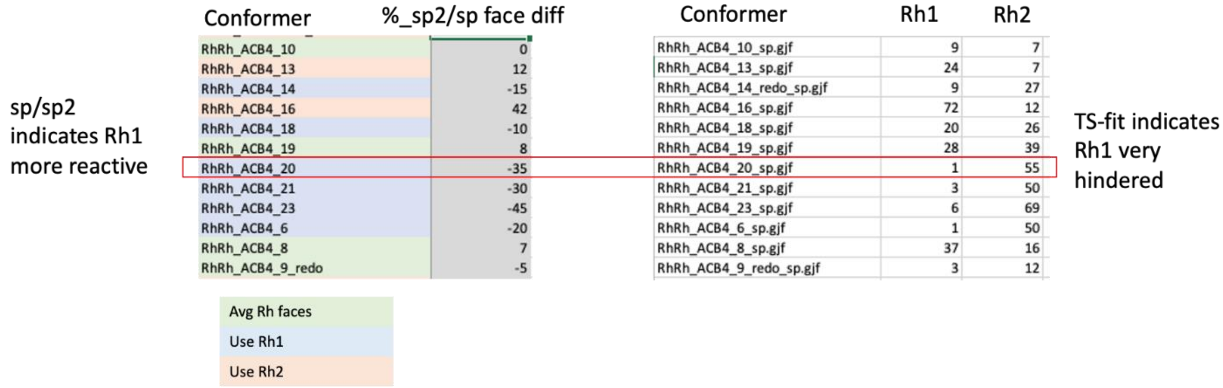

Conformer	%_sp2/sp face diff
RhRh_ACB4_10	0
RhRh_ACB4_13	12
RhRh_ACB4_14	-15
RhRh_ACB4_16	42
RhRh_ACB4_18	-10
RhRh_ACB4_19	8
RhRh_ACB4_20	-35
RhRh_ACB4_21	-30
RhRh_ACB4_23	-45
RhRh_ACB4_6	-20
RhRh_ACB4_8	7
RhRh_ACB4_9_redo	-5

Conformer	Rh1	Rh2
RhRh_ACB4_10_sp.gjf	9	7
RhRh_ACB4_13_sp.gjf	24	7
RhRh_ACB4_14_redo_sp.gjf	9	27
RhRh_ACB4_16_sp.gjf	72	12
RhRh_ACB4_18_sp.gjf	20	26
RhRh_ACB4_19_sp.gjf	28	39
RhRh_ACB4_20_sp.gjf	1	55
RhRh_ACB4_21_sp.gjf	3	50
RhRh_ACB4_23_sp.gjf	6	69
RhRh_ACB4_6_sp.gjf	1	50
RhRh_ACB4_8_sp.gjf	37	16
RhRh_ACB4_9_redo_sp.gjf	3	12

Avg Rh faces
Use Rh1
Use Rh2

Figure S5. Combined analysis with both classifiers.

b. Calculation of Catalyst Molecular Descriptors

Ensemble Parameters

Molecular descriptors for multiple conformations are condensed into single values representative of the entire ensemble. The default values are a Boltzmann-weighted average, which averages the conformer descriptors relative to the population weight of each conformation. Other descriptor suffixes are defined in Table S1.

Table S1: Ensemble Descriptor Suffixes

**Suffix**	**Definition**
N/A	Boltzmann-weighted average: $$\underline{x}* = \frac{\Sigma_{i=1}^{N} w_i\, x_i}{\Sigma_{i=1}^{N} w_i}$$ Where, w_i = Boltzmann weight for sample i; x_i = descriptor for sample i; N = number of samples
σ	Weighted standard deviation: $$\sigma = \sqrt{\frac{\Sigma_{i=1}^{N} w_i (x_i - \underline{x}*)^2}{\frac{(M-1)}{M} \Sigma_{i=1}^{N} w_i}}$$ Where, w_i = Boltzmann weight for sample i; x_i = descriptor for sample i; $\underline{x}*$ = Boltzmann-weighted descriptor average; M = number of nonzero weights; N = number of samples
MIN	Ensemble minimum descriptor value
MAX	Ensemble maximum descriptor value

Full-Pocket SMART Descriptors

Using the general workflow for SMART parameter generation[10], steric pocket parameters for both Rh faces were gathered for all catalyst conformations using a $S(SiF_2)_{10}$ molecular probe tethered at the sulfur atom 2.0 Å from Rh. Using MacroModel, probe conformers within an energy window of 41.9 kJ/mol (10.01 kcal/mol) were collected. Molecular surfaces were generated using USCF Chimera (v1.15) using the *molmap* command at an angstrom resolution of 2.8 and volume levels set at 0.07 and 4 for the catalyst and probe ensemble respectively. The parameters collected are summarized in Table S2.

Table S2. Full-Pocket SMART Steric Parameters

Abbreviation	Representative Quantity	Program
V_{CAVITY}	Pocket volume	Chimera
A_{CAVITY}	Pocket surface area	Chimera
CSA	Contact surface area between the catalyst and pocket surfaces	Chimera
ESA	Entry surface area, or unhindered pocket surface area: ESA = A_{CAVITY} - CSA	N/A
ψ	Sphericity Index: $\psi = \frac{\sqrt[3]{36\,V^2}}{A}$	N/A

Proximal SMART Descriptors

A series of intriguing experimental results highlighted the need for a more refined analysis of catalyst steric effects. When screened against the same substrate (**2c**), three catalysts with highly diverse core ligand scaffolds all yielded similar diastereoselectivity (Table S3). High diastereoselectivity observed in **C1** has been previously attributed to the hindrance of the chiral crown conformation, but for catalysts **C12** and **C17** a similar crown conformation is not feasible. Due to the relative stability of donor/donor Rh-carbene intermediates, variation in the steric environment exerted by ligand features *proximal* to Rh was of great interest in rectifying the performance of these diverse structures.

Table S3. Select diastereoselectivity results for 2c.

Substrate	Catalyst	Ligand	dr (*syn-anti*)
**2c**	**C1**	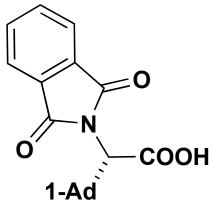	> 99:1
	**C12**	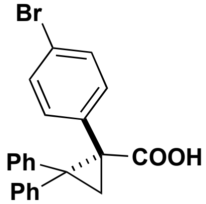	> 99:1
	**C17**	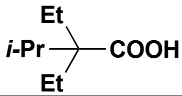	95:5

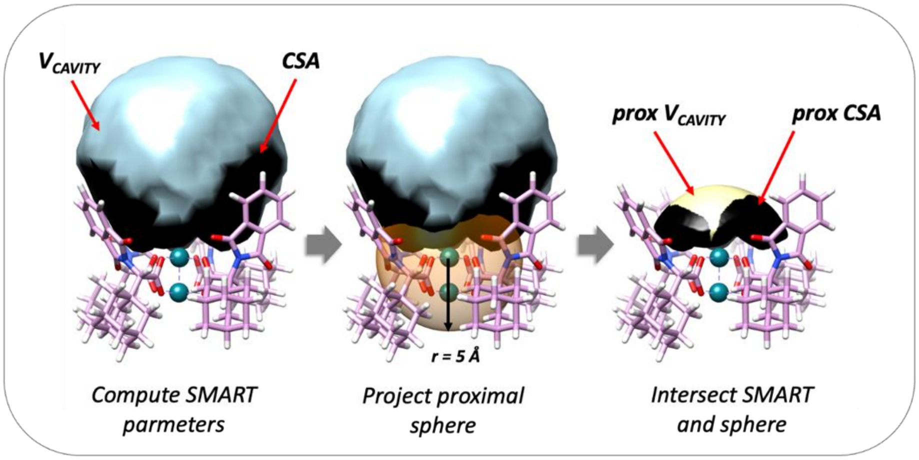

Figure S6. Generation of proximal SMART parameters depicted using a conformer of **C1**.

A modification to the above SMART parameter workflow yielded a novel set of *proximal* and *distal* pocket steric parameters. The proximal SMART parameters collected are summarized in Table S4. The pocket surface generated in Chimera was overlaid with a sphere projected from the Rh and the overlapping portion of the pocket within the sphere (named proximal) was separated from the remainder (Figure S6). A series of sphere radii were assessed (r = 5, 6, 7 Å) and the parameters gathered using the r = 5 Å sphere were used further in modeling. This *proximal* portion of the pocket was used to gather steric parameters as described above with the addition of the parameter denoted sphericity (ψ). This mathematical value quantifies how irregular (ψ = 1) or spherical (ψ = 0) the pocket is based on the ratio of volume to surface area defined by:

$$\psi = \frac{\sqrt[3]{36\,\pi\, V_{CAVITY}^2}}{A_{CAVITY}}$$

Table S4. Novel Proximal SMART Steric Parameters

**Abbreviation**	**Representative Quantity**	**Program**
proxV_{CAVITY}	Proximal pocket volume	Chimera
prox$V\%_{CAVITY}$	Percentage of full pocket volume proximal to Rh	
proxA_{CAVITY}	Proximal pocket surface area	Chimera
prox$A\%_{CAVITY}$	Percentage of full pocket surface area proximal to Rh	
proxCSA	Contact surface area between the catalyst and proximal pocket surfaces	Chimera
proxCSA%	Percentage of full pocket contact	

	surface area proximal to Rh	
proxESA	Entry surface area, or unhindered surface area proximal to Rh proxESA = proxSA - proxCSA	N/A
proxESA%	Percentage of full pocket entry surface area proximal to Rh	
proxψ	Proximal pocket sphericity	N/A

Full-Pocket G-Parameter (G%)

G-Parameter[23], or G%, for each Rh pocket was gathered using the WolframAlpha implemented program SolidAngle. In calculating this parameter for each face, the opposite Rh atom was changed by name to a dummy Xe atom to sterically block to opposite catalyst pocket, preventing the opposite face from interfering in the separate quantification of steric features at each pocket. One challenge in implementing the G% arises when catalysts with vastly different sizes are compared. The G% values in SolidAngle are calculated from structures normalized to fit within a sphere of a 1 Å radius. This can result in catalysts with the same G% descriptor presenting vastly different amounts of accessible surface area dependent upon their true size.

G% Surface Descriptor

G% surface was designed and implemented to capture the relative catalyst accessible surface area allowing for direct comparison between different structures. G% surface is calculated by casting the G% value computed from the normalized structure back onto the original sphere encompassing all atoms of the catalyst. The radius of this sphere is highly dependent on the size of the ligands and the conformation of the catalyst (Figure S7). The resultant values are calculated using the formula:

$$G\%\, surface = A_{SPHERE} * G\%$$

Where,
A_{SPHERE} = the area of the computed encompassing sphere, and
$G\%$ = the normalized G% calculated from SolidAngle values

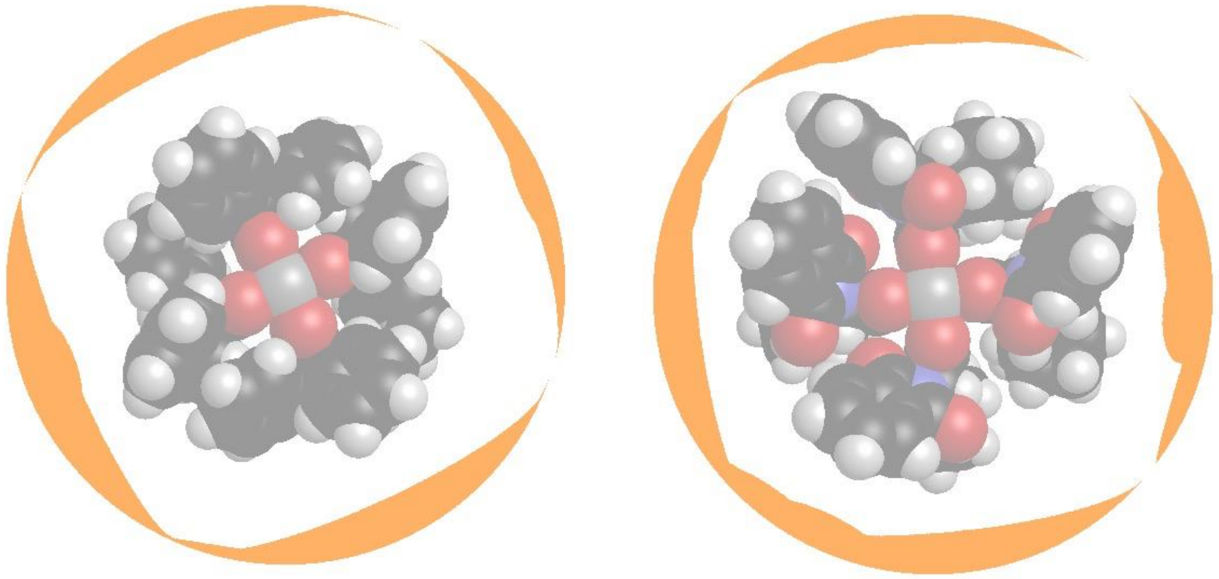

C23
Rh_2(triphenyl)$_4$
G% = 81.4%
G surface = 664 Å^2

C1
Rh_2(*S*-PTAD)$_4$
G% = 81.7%
G surface = 750Å^2

Figure S7. *G% surface* calculated from *G%*. Images generated using Solid-G.

Visible Volume (visVol) Descriptors

Visible volume was computed using the Python suite Morfeus (https://digital-chemistry-laboratory.github.io/morfeus/index.html). The Rh of each cavity was used at the central atom. Total volume visible (visVol) and visible volume percentage (visVol%) were used as computed.

Electronic Descriptors

Rh electronic descriptors were computed based on *d*-orbital overlap with a bound carbene substrate. NBO charges for each Rh were computed using the pop=nbo command implemented in Gaussian16. The energy and occupancy of the backbonding *dxz* and *dyz* orbitals were averaged (Rh π) and the energy and occupancy of the dz^2 orbital (Rh σ) was gathered. The descriptor Rh ΔE was implemented as described previously[10] by combining standardized values of both Rh π and Rh σ.

c. Substrates and *syn*-Products

Selecting Surrogates for Transition State Properties

Molecular descriptors used in modeling are selected based on the properties of the diastereo-determining step of the reaction mechanism. In the cyclization of benzodihyrofurans by Rh-catalyzed C-H insertion, the physical orientation between *syn* and *anti*-transition states differs significantly. The orientation of the substrate entering the diastereodetermining step is highly influential to the outcome. Abstraction of the hydride **TS-1** (S_E2) is hypothesized to be the

diastereodetermining step, as cyclization at insertion **TS-2** is likely rapid. Thus, stereochemistry in the transition state is largely determined by the identity of the initial C-H cite targeted.

As the steric environment of the *syn*-transition state is more structurally analogous to the cyclized *syn*-product than a bound linear carbene (Figure S8), steric properties were computed from the *syn*-product conformers. Electronic effects of substitution on the bound intermediate carbene would instead be more influenced by contributions of the donor groups to the carbene center, as well as the influence of the substituent on the insertion carbon. Free carbene conformers were computed as surrogates to bound Rh-carbenes and electronic molecular descriptors were gathered.

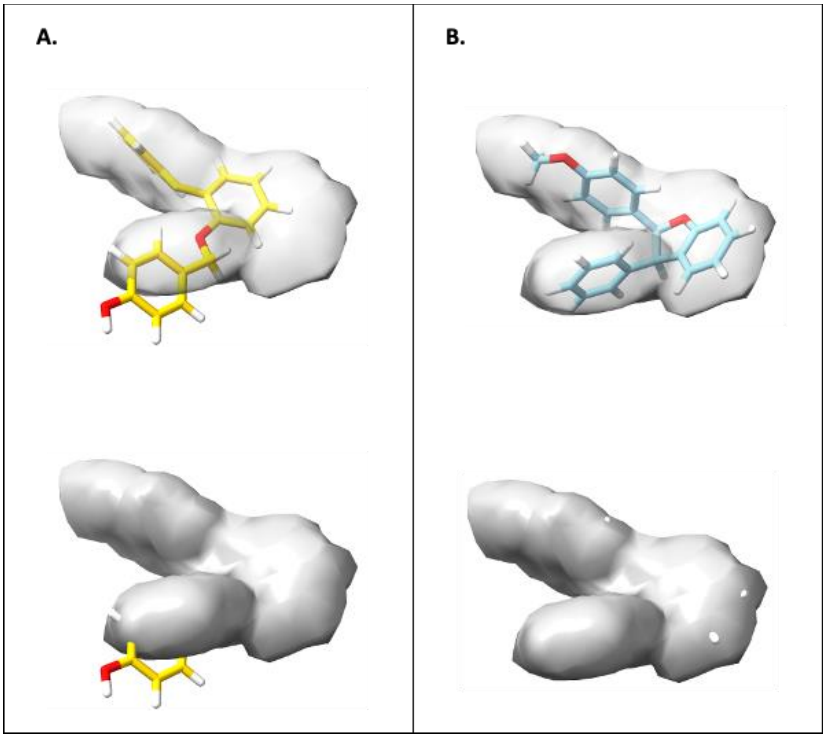

Figure S8. Comparison of free carbene and *syn*-product similarity to *syn*-transition state. Molecular density map in gray was generated in ChimeraX (molmap 2.8 level 0.07) from the published **TS-2a**. a) The lowest energy free carbene conformer does not fit into the *syn*-TS molecular density map, indicating that the steric environment of the TS is not well modeled by a free carbene. b) The lowest energy *syn*-product conformer fits into the map. It was hypothesized that the *syn*-product would be a suitable surrogate for steric properties describing the TS.

Generation and Calculation of Conformational Ensembles

Initial structures of benzophenone starting materials and *syn*-products were constructed in GaussView. Methoxy substituents were truncated to hydroxyl groups to minimize the number of rotamers. Conformational searches were performed in the gas phase using the molecular mechanics force field OPLS3e as employed in MacroModel with an energy window of 20.9 kJ/mol (5.00 kcal/mol). Searches were performed with no solvent using mixed torsional/low-mode sampling. The ketone oxygen atom of the benzophenone conformers was then removed to produce the analogous free carbene structure for each conformer.

Free carbenes were calculated in the singlet state[24] as the empty *p*-orbitals of the singlet carbene are stabilized through conjugation from the two donating substituents and π-backbonding from the dirhodium catalyst. Representative conformers were geometrically optimized in the gas phase at the B3LYP/6-31G* level of theory using Grimme's dispersion correction with Becke-Johnson damping (BJD3). Frequency calculations were performed to ensure that optimized geometries were not converged to an imaginary frequency. Single point energies were calculated at the M06-2X/6-311** level of theory using the SMD method in dichloromethane. Relative free energies utilize the solvated electronic energies to correct the gas phase free energies. NBO and NMR analysis was conducted to obtain electronic descriptors.

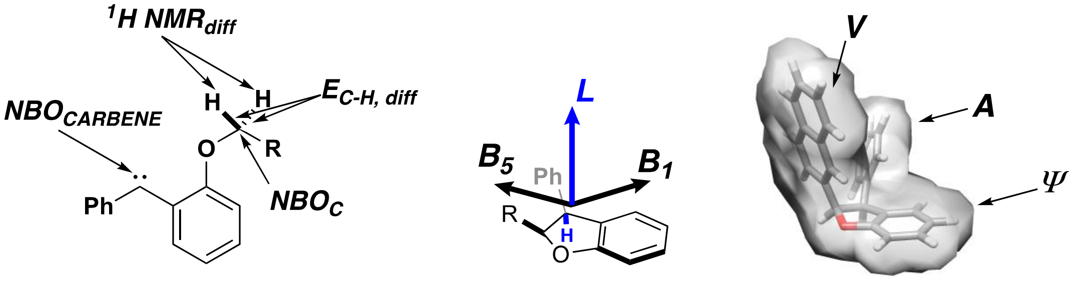

3b

Figure S9. Summary of Substrate and Product Molecular Descriptors

Calculation of Substrate and Product Molecular Descriptors

Steric molecular descriptors computed from products included shape descriptors such as volume (V) and surface area (A) computed in UCSF Chimera. Sphericity was computed as described above. Sterimol[25,26] descriptors describing the height, minimum width, and maximum width (L, B_1, and B_5 respectively) were computed using the C-H bond depicted in Figure S9 as the L-axis.

Electronic descriptors were computed from the free carbene surrogates describing the carbene C and cite of insertion. NBO charges were computed for the carbene C ($NBO_{CARBENE}$) and the insertion C-H_2 (NBO_C). The difference between syn- and anti-hydrogen was considered by parametrizing the difference between their C-H bond energies ($E_{CH,diff}$) and computed ^{1}H NMR signals (^{1}H NMR_{diff}). Additionally, the number of carbene conformers (N_{confs}) was included to describe flexibility. A summary of product and substrate descriptors is given in Figure S9.

d. Selecting Diverse Structures by PCA

Catalyst Parametrization and Selection of Representatives

Initial structures for each dirhodium catalyst were reported X-ray crystal structures deposited in the CSD. Catalysts for which no crystal structure was found were constructed from X-ray structures of the closest corresponding crystal structure. The single crystal structure conformers were geometrically optimized and computed at a single point as described previously.

High-level molecular descriptors were gathered describing the the steric ligand environment of the catalyst pocket (SMART parameters and G%). Additionally, a binary symmetry classifier was applied to denote catalysts with asymmetric ligands about the carboxylate axis (Figure S10) that could produce asymmetric pockets (0 assigned to symmetric, 1 assigned to asymmetric) and the rotatable bond count (RBC) of the free ligand calculated in MacroModel.

These parameters (23 total) were reduced to 23 dimensions using principal component analysis (PCA). The 2 PCs with the highest percentage of explained variance were used to construct a 2D chemical space map (Figure S11).

Analysis of the resultant chemical space found that ligands with similar core scaffolds were relegated to distinct regions. The previously tested catalysts were identified in chemical space and were found to be concentrated in the phthalimide ligand scaffold region with singleton selections located in the unhindered and chiral crown regions. Representative structures were proposed to capture the breadth of substituent effects within the scaffold classes (local), as well as explore unsampled regions of chemical space (global).

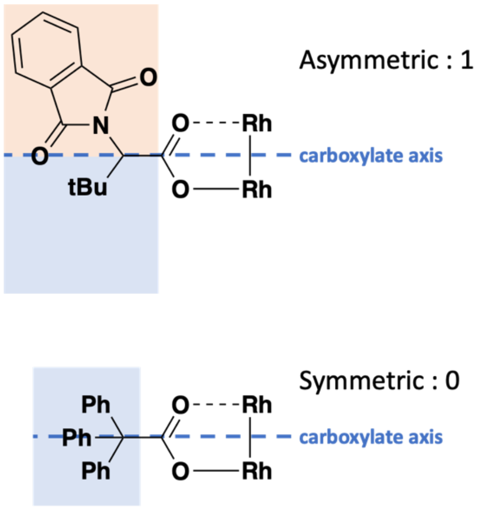

Figure S10. Depiction of symmetry classifier for catalyst ligands.

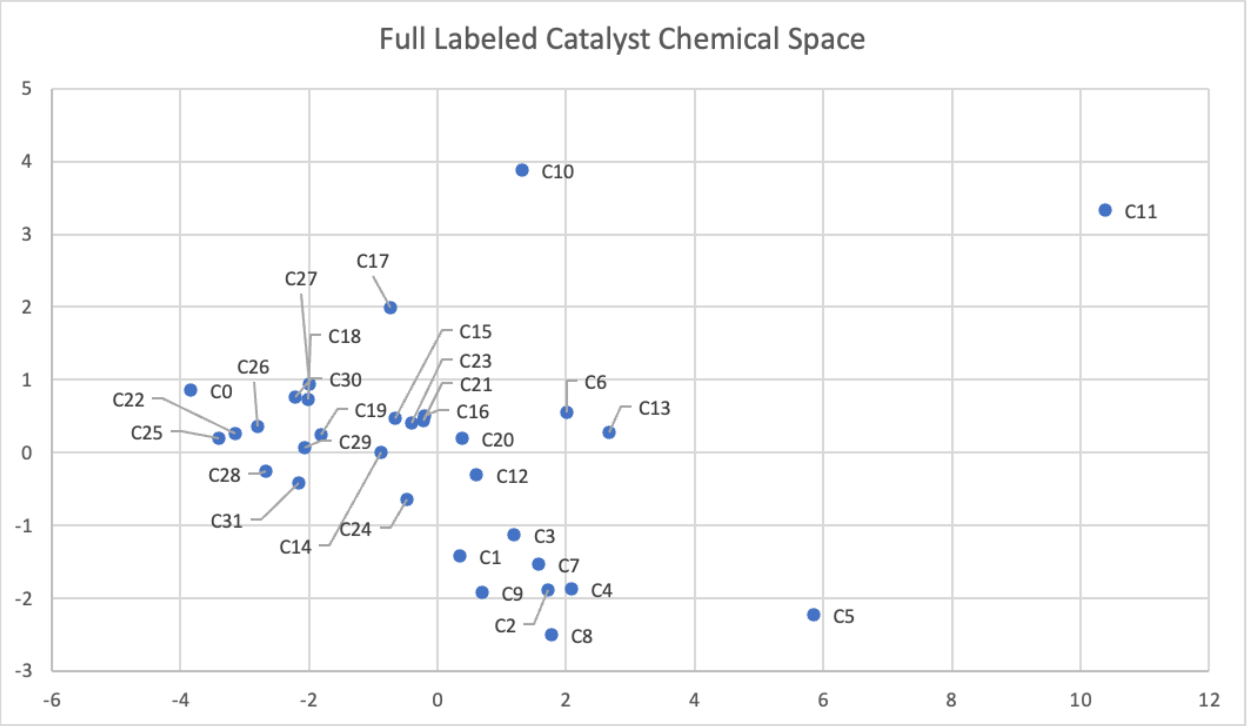

Figure S11. PC catalyst space.

Substrate Parametrization and Selection of Representatives

To assess the breadth of diversity for substrate structures, all commercial alkyl bromide synthons amenable to the synthesis of the target substituted benzophenone core were gathered as SMILES strings from REAXYS and parameterized with the 2D Mordred descriptor set as implemented in the RDKit Python package. These parameters were reduced to 10 dimensions (PCs) using PCA. The 2 PCs with the highest percentage of explained variance were used to construct a 2D chemical space map (Figure S12).

Analysis of relative trends in the spread of structural properties across chemical space found that substrate size and flexibility generally increase with PC 2 and deactivating nature generally increases with PC 1. Substrates were selected from a subregion where flexibility was not expected to detrimentally affect the reaction and substituents would not be so deactivating as to inhibit cyclization (Figure S13).

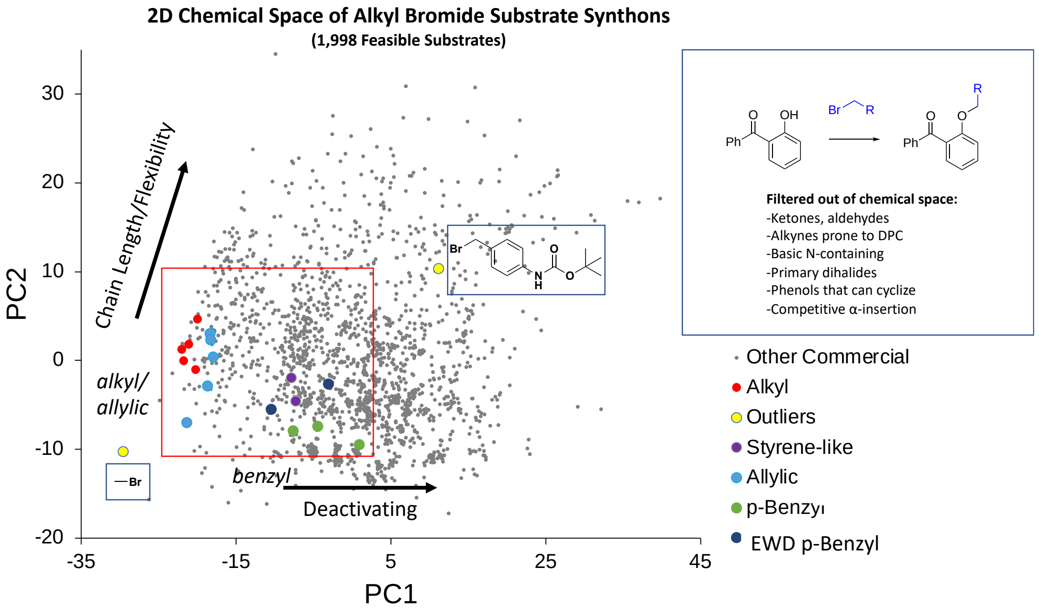

Figure S12. Full alkyl bromide synthon chemical space using 2D Mordred descriptors.

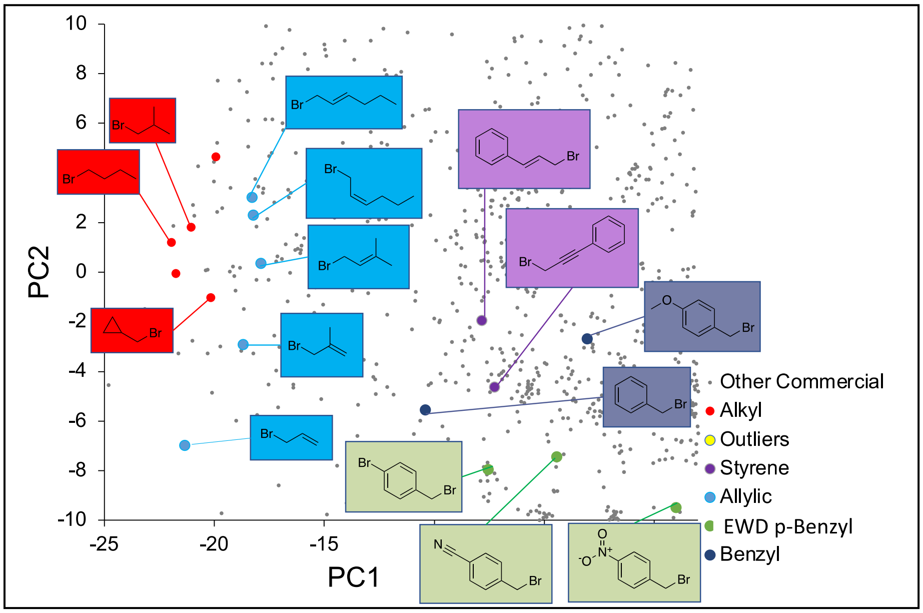

Figure S13. Subspace of alkyl bromide synthons for substrate selection.

e. Linear Modeling

Discussion of Linear Modeling Predictions

All data points determined to be 100:0 dr were excluded from modeling due to measurement sensitivity. Initial attempts to model linear molecular descriptors using MLR were unsuccessful, and the resultant models performed disparately in the training set. The five data points with catalyst Rh_2(*S*-PTAD)$_4$ (**C1**) was predicted robustly (R^2 = 0.71, MAE = 0.28) (Figure S14). This indicates that the linear model could discern the relationship between chiral crown catalysts and diastereoselectivity. However, ten data points of catalyst $Rh_2(OAc)_4$ (**C0**) were poorly predicted (R^2 = 0.67, MAE = 0.30) with a notable tendency for under prediction of *syn*-selectivity (Figure S14). Novel achiral catalysts **C14** and **C15** were generally well predicted (R^2 = 0.65, MAE = 0.31), likely due to their structural proximity to chiral crown catalysts. This not only indicates that the model is unable to accommodate unhindered catalyst such as **C0**, but also that these catalysts may follow different structure-function relationships. Similar disparate predictions for the external test set were observed.

Substrates effects were not adequately captured in training, evident by the vastly different predictions between three aryl substituents (Figure S15). While the predictions for **2d** were moderately uniform, **2a** and **2c** were unable to be predicted. Additionally, external substrate **2b**, selected by structural diversity from training substrates, was significantly overpredicted indicated by the quantity of data points under the prediction line. This led us to conclude that substrate effects were incorrectly modeled.

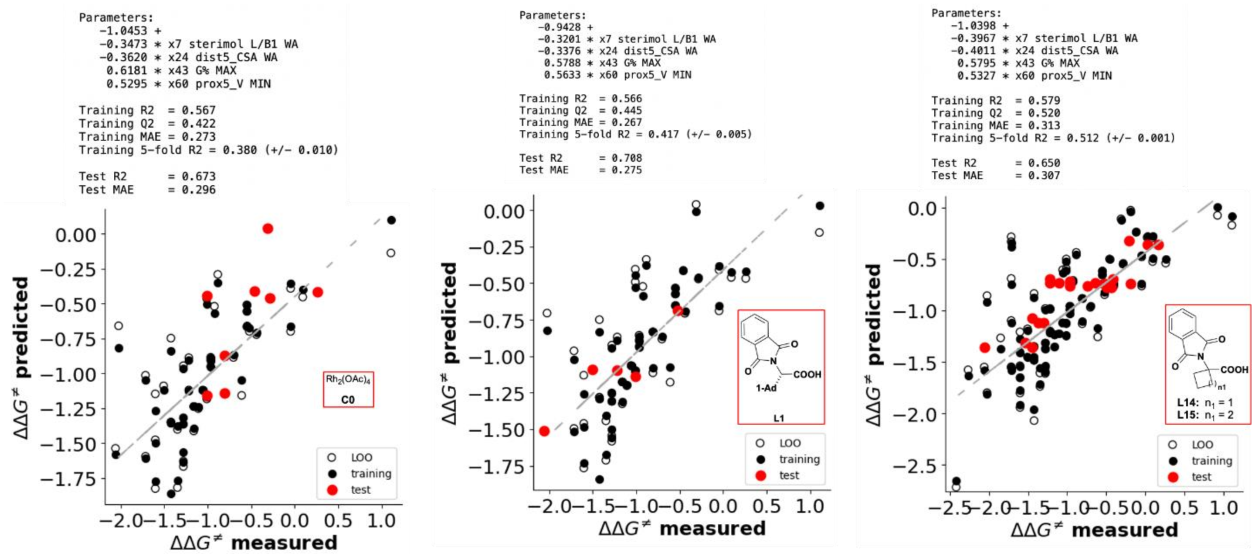

Figure S14. Training predictions for catalysts $Rh_2(OAc)_4$ **(C0)** showing general underprediction, and b) catalyst Rh_2(*S*-PTAD)$_4$ (**C1**). External predictions for **C14** and **C15**.

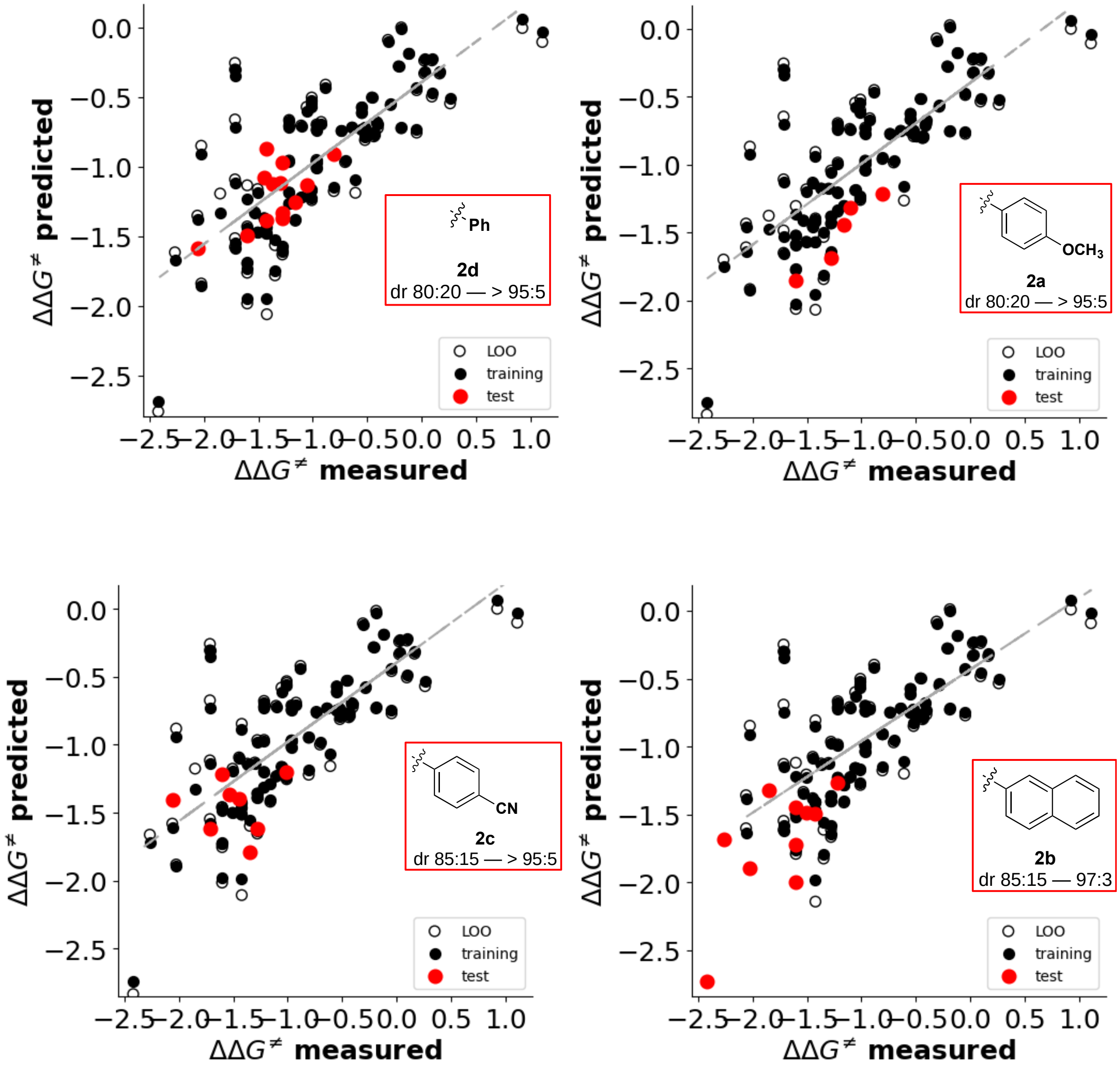

Figure S15. Predictions of substrates showing significant deviations in prediction ability for both training and external test structures with aryl substituents.

Table S5. Modeling Data

		dr		
**Substrate**	**Catalyst**	***syn***	***anti***	***ΔΔG‡ (kcal/mol) 298.15 K***
**2a**	**C14**	100 (99.5)	0 (0.5)	-3.135881082
	**C16**	100 (99.5)	0 (0.5)	-3.135881082
	**C15**	100 (99.5)	0 (0.5)	-3.135881082
	**C10**	87	13	-1.126173688
	**C29**	88	12	-1.180363548
	**C0**	80	20	-0.82127412
	**C20**	90	10	-1.301688683

	**C7**	94	6	-1.630075694
	**C1**	100 (99.5)	0 (0.5)	-3.135881082
	**C4**	100 (99.5)	0 (0.5)	-3.135881082
**2d**	**C14**	91	9	-1.370652975
	**C16**	92	8	-1.446905122
	**C15**	90	10	-1.301688683
	**C10**	90	10	-1.301688683
	**C31**	92	8	-1.446905122
	**C17**	96	4	-1.882755521
	**C18**	93	7	-1.532416977
	**C29**	86	14	-1.075421434
	**C0**	80	20	-0.82127412
	**C20**	90	10	-1.301688683
	**C27**	58	42	-0.19121872
	**C26**	68	32	-0.446552544
	**C28**	53	47	-0.07117638
	**C19**	92	8	-1.446905122
	**C7**	94	6	-1.630075694
	**C1**	97	3	-2.059324464
	**C4**	100 (99.5)	0 (0.5)	-3.135881082
	**C4**	72	28	-0.559521771
	**C6**	92	8	-1.446905122
	**C25**	82	18	-0.898320721
	**C22**	80	20	-0.82127412
**2h**	**C14**	70	30	-0.50195963
	**C16**	75	25	-0.650844341
	**C15**	68	32	-0.446552544
	**C10**	72	28	-0.559521771
	**C29**	68	32	-0.446552544
	**C0**	62	38	-0.290020142
	**C20**	84	16	-0.982374193
	**C9**	77	23	-0.715832618
	**C1**	85	15	-1.027619382
	**C4**	46	54	0.094990842
**2g**	**C14**	71	29	-0.530447043
	**C16**	67	33	-0.41954586
	**C15**	58	42	-0.19121872
	**C10**	72	28	-0.559521771
	**C29**	52	48	-0.047419225

	**C0**	39	61	0.264998516
	**C20**	84	16	-0.982374193
	**C7**	74	26	-0.619656928
	**C1**	89	11	-1.238605308
	**C4**	52	48	-0.047419225
**2i**	**C14**	89	11	-1.238605308
	**C16**	89	11	-1.238605308
	**C15**	87	13	-1.126173688
	**C10**	85	15	-1.027619382
	**C29**	72	28	-0.559521771
	**C0**	68	32	-0.446552544
	**C20**	89	11	-1.238605308
	**C9**	97	3	-2.059324464
	**C1**	93	7	-1.532416977
	**C4**	82	18	-0.898320721
**2j**	**C14**	78	22	-0.749811199
	**C16**	84	16	-0.982374193
	**C15**	84	16	-0.982374193
	**C17**	84	16	-0.982374193
	**C0**	85	15	-1.027619382
	**C9**	100 (99.5)	0 (0.5)	-3.135881082
	**C1**	100 (99.5)	0 (0.5)	-3.135881082
	**C11**	100 (99.5)	0 (0.5)	-3.135881082
	**C12**	92	8	-1.446905122
	**C23**	95	5	-1.744356465
	**C22**	68	32	-0.446552544
**2c**	**C14**	97	3	-2.059324464
	**C16**	93	7	-1.532416977
	**C15**	92	8	-1.446905122
	**C17**	95	5	-1.744356465
	**C0**	85	15	-1.027619382
	**C9**	90	10	-1.301688683
	**C11**	94	6	-1.630075694
	**C1**	100 (99.5)	0 (0.5)	-3.135881082
	**C12**	100 (99.5)	0 (0.5)	-3.135881082
	**C23**	91	9	-1.370652975
	**C22**	88	12	-1.180363548
**2e**	**C14**	49	51	0.023700122
	**C16**	55	45	-0.118882146

	**C15**	43	57	0.166975401
	**C0**	58	42	-0.19121872
	**C1**	66	34	-0.392951447
	**C4**	16	84	0.982374193
	**C12**	92	8	-1.446905122
	**C23**	86	14	-1.075421434
**2b**	**C14**	92	8	-1.446905122
	**C16**	94	6	-1.630075694
	**C15**	93	7	-1.532416977
	**C17**	94	6	-1.630075694
	**C0**	85	15	-1.027619382
	**C9**	89	11	-1.238605308
	**C1**	98	2	-2.305607943
	**C1**	94	6	-1.630075694
	**C11**	96	4	-1.882755521
	**C23**	97	3	-2.059324464
	**C22**	87	13	-1.126173688
**2f**	**C14**	49	51	0.023700122
	**C16**	59	41	-0.215621843
	**C15**	43	57	0.166975401
	**C0**	63	37	-0.31529804
	**C1**	71	29	-0.530447043
	**C4**	13	87	1.126173688
	**C12**	91	9	-1.370652975
	**C23**	83	17	-0.939363725
**2k**	**C14**	95	5	-1.744356465
	**C16**	95	5	-1.744356465
	**C12**	95	5	-1.744356465
	**C23**	95	5	-1.744356465

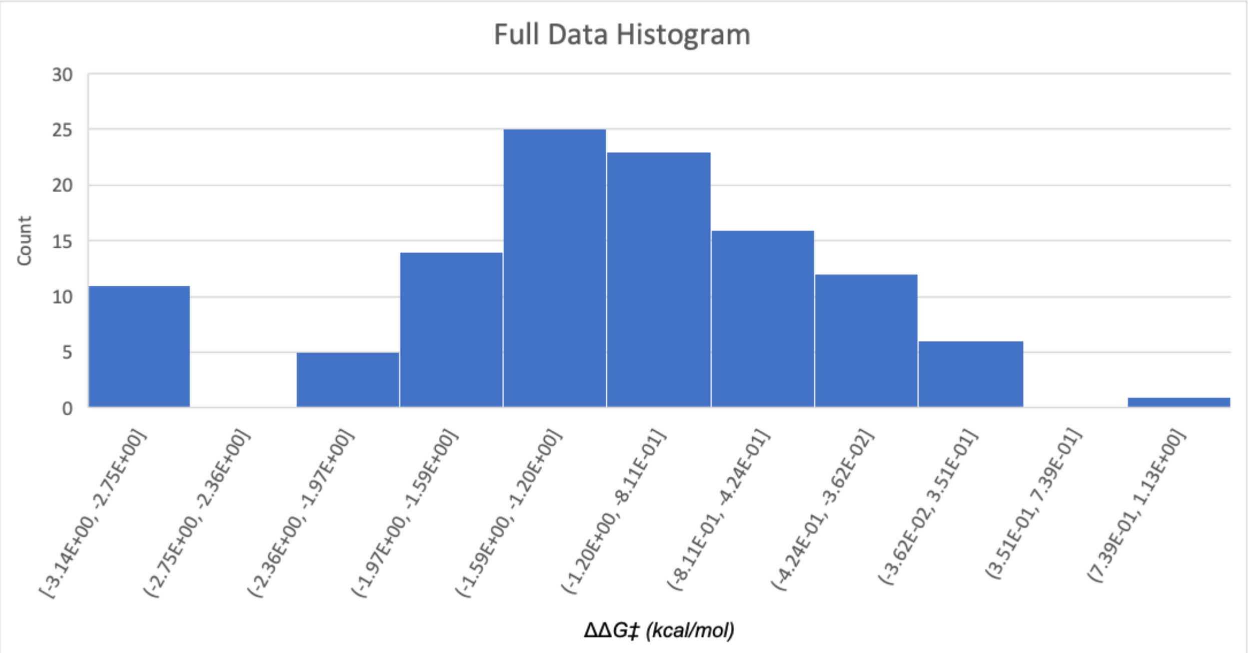

Figure S16. Histogram of Modeling Data Spread

f. Nonlinear Parameter Generation and Modeling

Implementation of a Modified SISSO Algorithm

The SISSO algorithm proposed by Ghiringhelli and coworkers[27] has recently been applied to contemporary machine learning efforts on complex data sets in materials science.[28,29] This algorithm was designed to identify nonlinear relationships between descriptors and a measured experimental outcome. The resultant nonlinear parameters generated by this algorithm have great potential for application to modeling in other areas of organic synthesis and catalysis. The insight that can be gained from these parameters can be viewed from the perspective of integrated rate laws. While concentration of a reaction with order higher than zero cannot be linearly plotted against time, transforming the concentration through the natural logarithm function accounts for the exponential relationship between the parameters, allowing a linear plot to be constructed. The SISSO algorithm can be fundamentally understood in a similar way, where the application of a variety of algebraic functions transforms parameters by accounting for nonlinear relationships that can then be linearly regressed against an experimental output.

The nonlinear descriptor set used in modeling was generated from a subset of gathered linear descriptors. As seen in the performance of the most viable linear models, a small subset of catalyst descriptors was repeatedly identified in best performing models, while the substrate parameters varied widely with no observed trend of significance. The catalyst parameters identified by the best performing linear models were selected and combined with all substrate parameters for generation of the nonlinear descriptor set.

A Python code was written implementing the SISSO algorithm as described in the 2019 publication, with the addition of statistical cut offs to reduce the number of redundant parameters in modeling. Univariate and bivariate transformations performed on the descriptor set in each recursion are listed in Table S6. A collinearity cut-off of $R^2 = 0.95$ between candidate descriptors and saved descriptors was used in each iteration. Additionally, each candidate descriptor p-value was compared against the minimum p-value calculated from the input linear descriptors, to ensure

that the complexity introduced by nonlinearity was not less statistically significant than the original linear set. Lastly, a domain cutoff was implemented to remove nonlinear descriptors generated for inputs that experienced algebraic domain violations. This code ran in two recursive iterations on 19 linear descriptors, resulting in 467 nonlinear descriptors to be used in modeling.

Table S6. Algebraic Functions Used in SISSO Descriptor Generation

**Univariate Operators**	**Bivariate Operators**	**Domain Restrictions**
a^2, a^3, e^a	$\lvert a - b \rvert, a * b$	None
a^{-1}	$\frac{b}{a}$	$\{a \mid a \neq 0\}$
N/A	$\frac{a}{b}$	$\{b \mid b \neq 0\}$
$\sqrt{a}$	N/A	$\{a \mid a \geq 0\}$
$log_{10}a, log_e a$	N/A	$\{a \mid a > 0\}$

Nonlinear Modeling Predictions

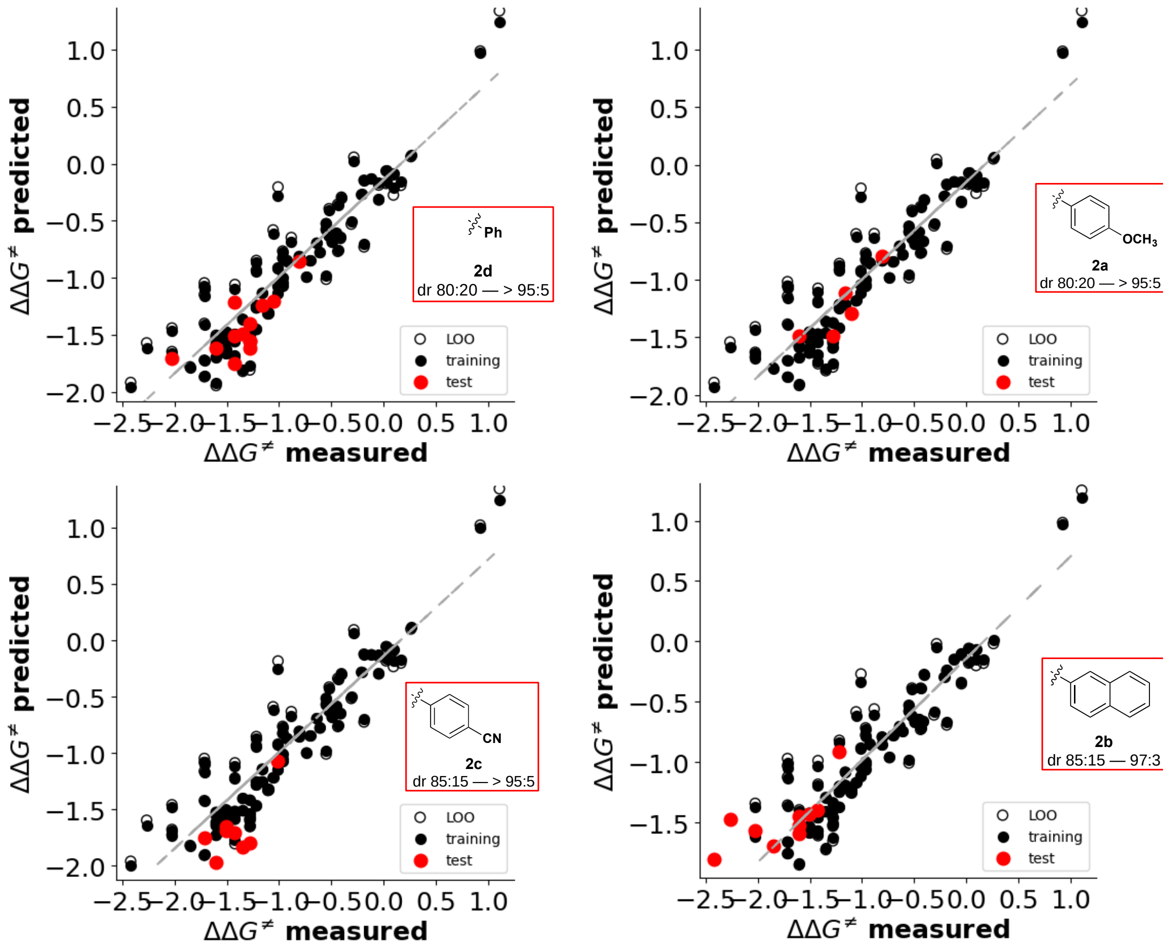

Figure S17. Predictions for different substrates with nonlinear descriptors.

Decision Tree for Optimizing *syn*-Selectivity

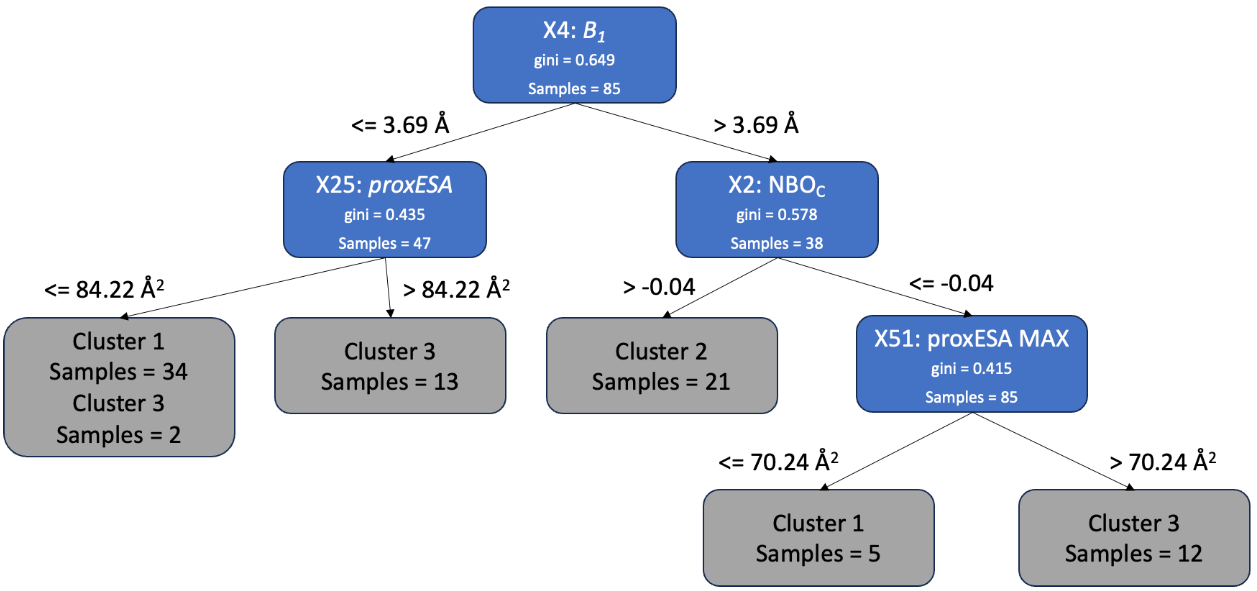

Figure S18. Decision tree for clusters 1-3 generated from Python package sklearn. Maximum tree depth = 3, seed = 5. One terminal node is impure (cluster 1; 34 samples, and cluster 3; 2 samples).

Additional PCA Trends for Nonlinear Model

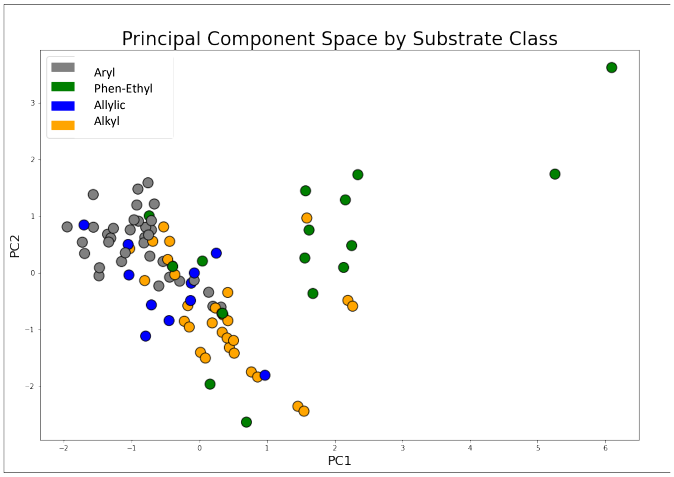

Figure S19. Chemical space of nonlinear model sorted by substrate substituent class. Aryl substrates generally perform the most syn-selectively while other classes are more disparate.

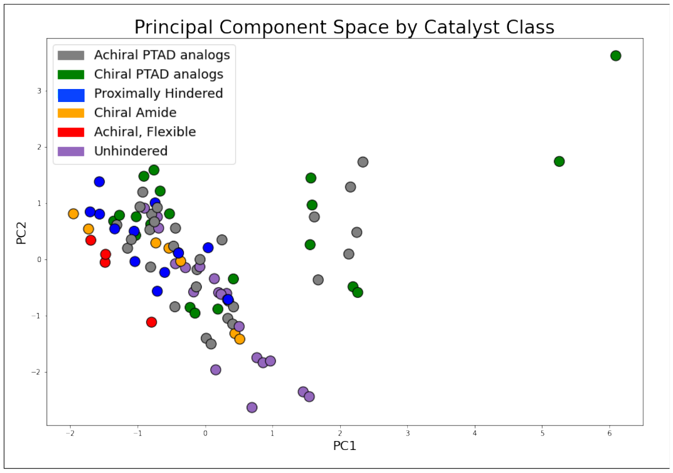

Figure S20. Chemical space of nonlinear model sorted by catalyst ligand class. No apparent trend in catalyst class is identified.

g. Hydride Abstraction Transition State Calculations

Transition states were geometrically optimized at the B3LYP/6-31+G(d) (LANL2DZ Rh) level per the protocol employed by Shaw and Fox.[6] The value of the imaginary frequency for **TS-1a** is in good agreement with their calculations. Overall, aryl substituents were found to have lower energy barriers to hydride abstraction than alkyl substituents. This is hypothesized to be due to π-stacking between aryl substituents stabilizing the conformation required for cyclization.

I1a

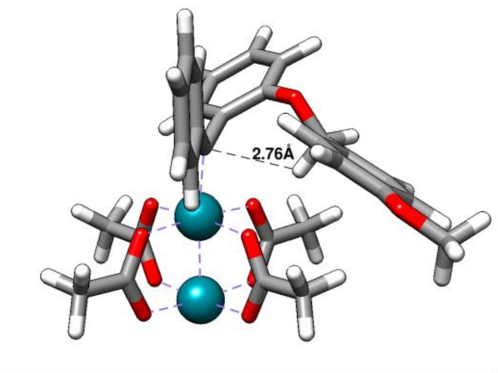

TS-1a

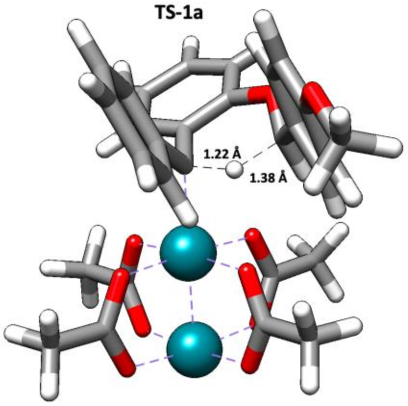

Imaginary frequency: 584.41*i* cm^{-1}

I1b

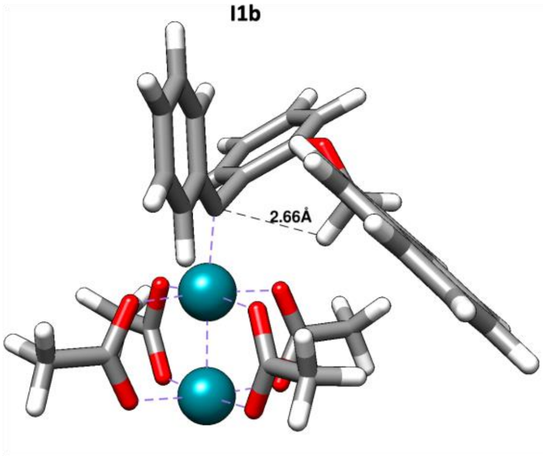

TS-1b

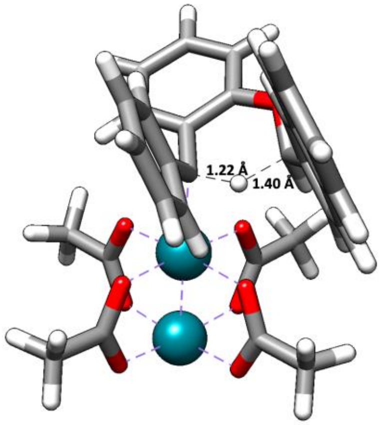

Imaginary frequency: 712.93*i* cm^{-1}

I1d

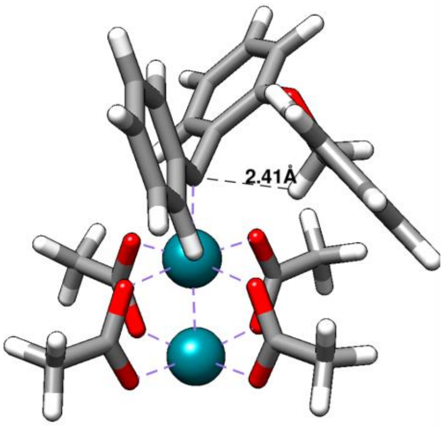

TS-1d

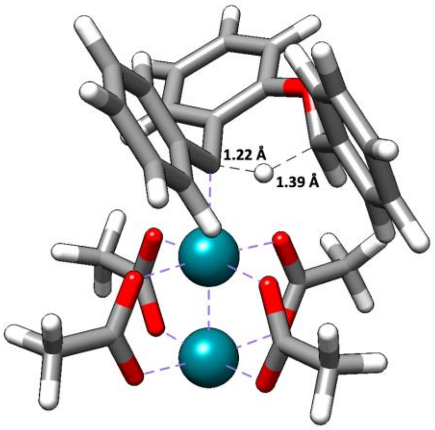

Imaginary frequency: 618.39*i* cm^{-1}

I1g

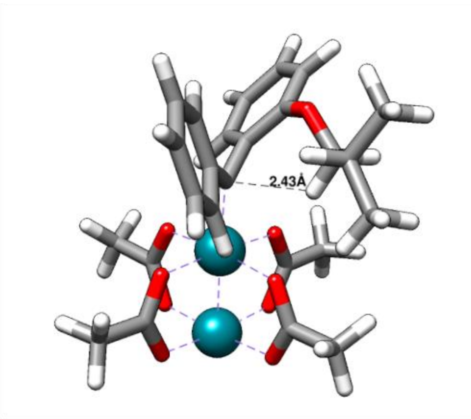

TS-1g

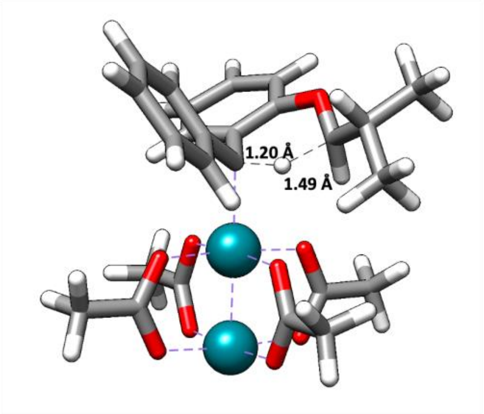

Imaginary frequency: 380.49*i* cm^{-1}

I1h

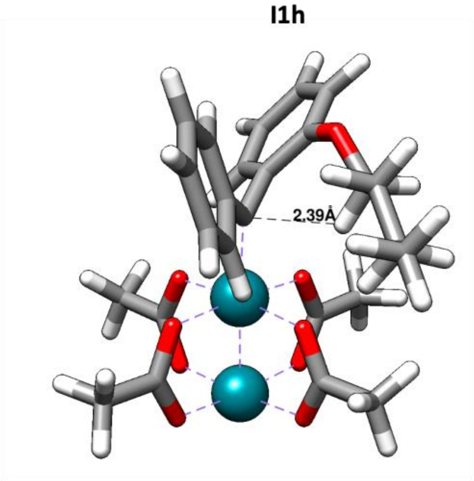

TS-1h

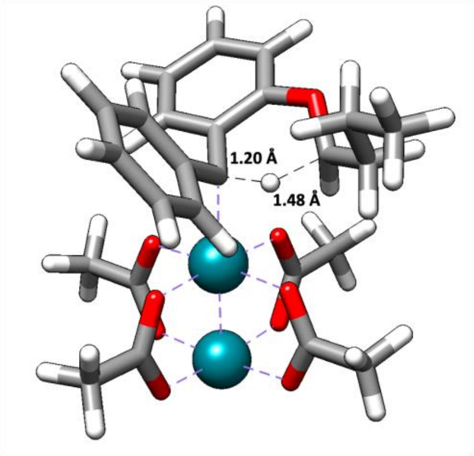

Imaginary frequency: 358.49*i* cm^{-1}

I1i

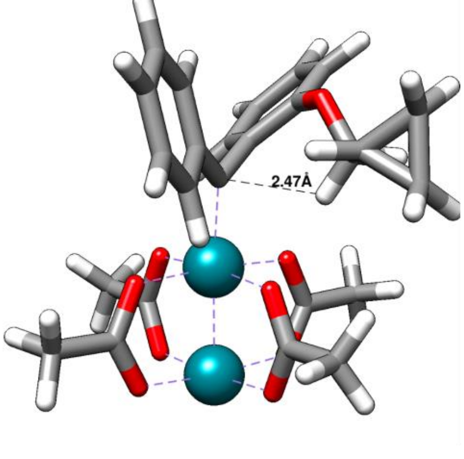

TS-1i

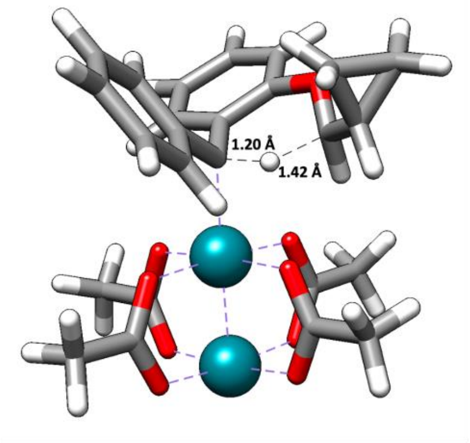

Imaginary frequency: 575.94*i* cm^{-1}

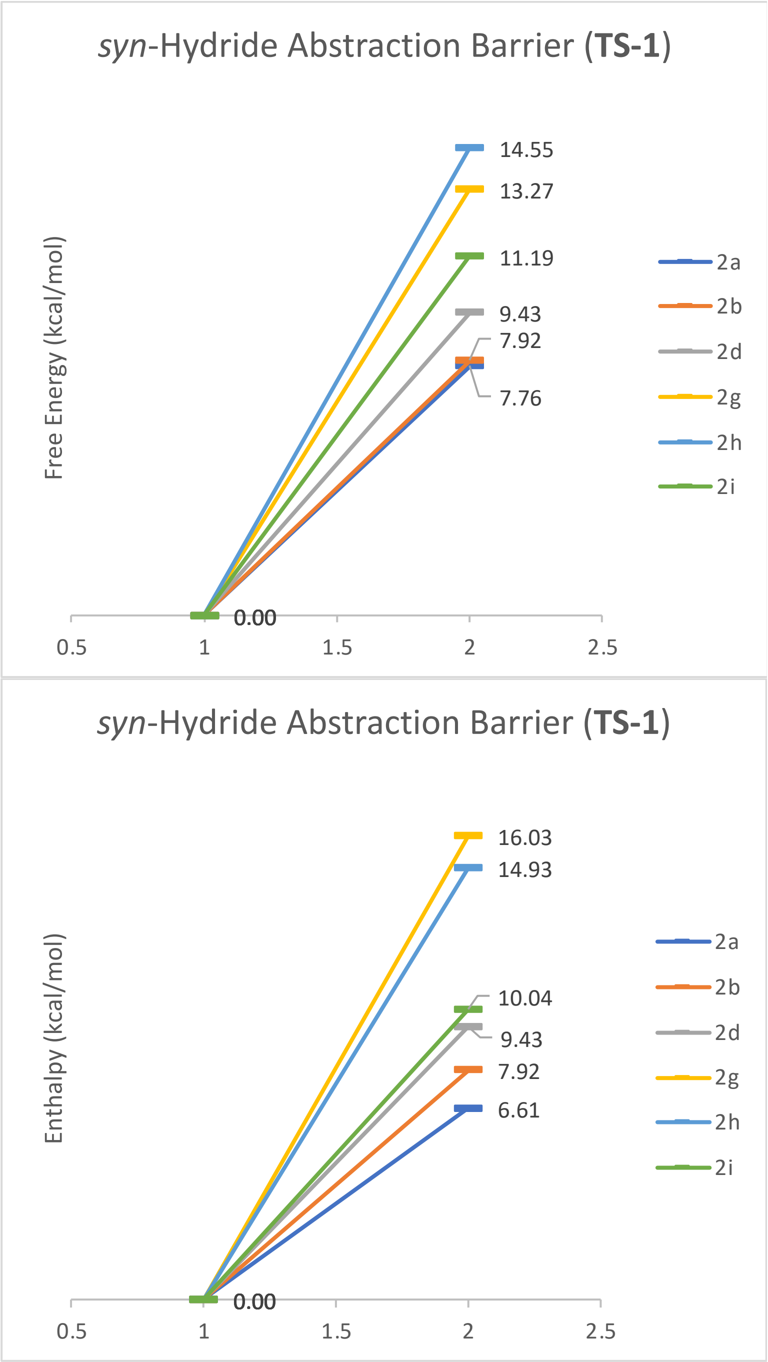

Figure S21. Pre-cyclization intermediate **I1** and **TS-1** computed for select substrates with catalyst **C0**. Substrates with aryl alkoxy substituents had lower energy barriers for insertion in free energy and enthalpy (kcal/mol).

IV. References

(1) Mattiza, J. T.; Fohrer, J. G. G.; Duddeck, H.; Gardiner, M. G.; Ghanem, A. Optimizing Dirhodium(II) Tetrakiscarboxylates as Chiral NMR Auxiliaries. *Org Biomol Chem* **2011**, *9* (19), 6542–6550. https://doi.org/10.1039/c1ob05665d.

(2) DeAngelis, A.; Panish, R.; Fox, J. M. Rh-Catalyzed Intermolecular Reactions of α-Alkyl-α-Diazo Carbonyl Compounds with Selectivity over β-Hydride Migration. *Acc Chem Res* **2016**, *49* (1), 115–127. https://doi.org/10.1021/acs.accounts.5b00425.

(3) Adly, F. G.; Bollard, H.; Gardiner, M. G.; Ghanem, A. Chiral Dirhodium(II) Carboxylates: New Insights into the Effect of Ligand Stereo-Purity on Catalyst Structure and Enantioselectivity. *Catalysts* **2018**, *8* (7), 1–13. https://doi.org/10.3390/catal8070268.

(4) Bergstrom, B. D.; Nickerson, L. A.; Shaw, J. T.; Souza, L. W. Transition Metal Catalyzed Insertion Reactions with Donor/Donor Carbenes. *Angewandte Chemie - International Edition* **2021**, *60* (13), 6864–6878. https://doi.org/10.1002/anie.202007001.

(5) Soldi, C.; Lamb, K. N.; Squitieri, R. A.; González-López, M.; Di Maso, M. J.; Shaw, J. T. Enantioselective Intramolecular C-H Insertion Reactions of Donor-Donor Metal Carbenoids. *J Am Chem Soc* **2014**, *136* (43), 15142–15145. https://doi.org/10.1021/ja508586t.

(6) Lamb, K. N.; Squitieri, R. A.; Chintala, S. R.; Kwong, A. J.; Balmond, E. I.; Soldi, C.; Dmitrenko, O.; Castiñeira Reis, M.; Chung, R.; Addison, J. B.; Fettinger, J. C.; Hein, J. E.; Tantillo, D. J.; Fox, J. M.; Shaw, J. T. Synthesis of Benzodihydrofurans by Asymmetric C−H Insertion Reactions of Donor/Donor Rhodium Carbenes. *Chemistry - A European Journal* **2017**, *23* (49), 11843–11855. https://doi.org/10.1002/chem.201701630.

(7) Souza, L. W.; Squitieri, R. A.; Dimirjian, C. A.; Hodur, B. M.; Nickerson, L. A.; Penrod, C. N.; Cordova, J.; Fettinger, J. C.; Shaw, J. T. Enantioselective Synthesis of Indolines, Benzodihydrothiophenes, and Indanes by C−H Insertion of Donor/Donor Carbenes. *Angewandte Chemie - International Edition* **2018**, *57* (46), 15213–15216. https://doi.org/10.1002/anie.201809344.

(8) Roos, K.; Wu, C.; Damm, W.; Reboul, M.; Stevenson, J. M.; Lu, C.; Dahlgren, M. K.; Mondal, S.; Chen, W.; Wang, L.; Abel, R.; Friesner, R. A.; Harder, E. D. OPLS3e: Extending Force Field Coverage for Drug-Like Small Molecules. *J Chem Theory Comput* **2019**, *15* (3), 1863–1874. https://doi.org/10.1021/acs.jctc.8b01026.

(9) MacroModel. Schrödinger LLC: New York, NY.

(10) Cammarota, R. C.; Liu, W.; Bacsa, J.; Davies, H. M. L.; Sigman, M. S. Mechanistically Guided Workflow for Relating Complex Reactive Site Topologies to Catalyst Performance in C − H Functionalization Reactions. **2021**, *2*. https://doi.org/10.1021/jacs.1c12198.

(11) Kelley, L. A.; Gardner, S. P.; Sutcliffe, M. J. An Automated Approach for Clustering an Ensemble of NMR-Derived Protein Structures into Conformationally Related Subfamilies. *Protein Eng* **1996**, *9* (11), 1063–1065. https://doi.org/10.1093/protein/9.11.1063.

(12) Becke, A. D. Density-Functional Thermochemistry. III. The Role of Exact Exchange. *J Chem Phys* **1993**, *98* (7), 5648–5652. https://doi.org/10.1063/1.464913.

(13) Schröder, H.; Creon, A.; Schwabe, T. Reformulation of the D3(Becke-Johnson) Dispersion Correction without Resorting to Higher than C6 Dispersion Coefficients. *J Chem Theory Comput* **2015**, *11* (7), 3163–3170. https://doi.org/10.1021/acs.jctc.5b00400.

(14) Hay, P. J.; Wadt, W. R. Ab Initio Effective Core Potentials for Molecular Calculations. Potentials for the Transition Metal Atoms Sc to Hg. *J Chem Phys* **1985**, *82* (1), 270–283. https://doi.org/10.1063/1.448799.
(15) Mantzaris, G. A. P. B. G. T. A. A.-L. A. S. J. A Complete Basis Set Model Chemistry. I. The Total Energies of Closed-Shell Atoms and Hydrides of the First-Row Atoms. *J. Chem. Phys* **1988**, *89*, 2193–2218.
(16) Andrae, D.; Häußermann, U.; Dolg, M.; Stoll, H.; Preuß, H. Energy-Adjusted Ab Initio Pseudopotentials for the Second and Third Row Transition Elements. *Theor Chim Acta* **1990**, *77* (2), 123–141. https://doi.org/10.1007/BF01114537.
(17) Frisch, M. J.; Trucks, G. W.; Schlegel, H. B.; Scuseria, G. E.; Robb, M. A.; Cheeseman, J. R.; Scalmani, G.; Barone, V.; Petersson, G. A.; Nakatsuji, H.; Li, X.; Caricato, M.; Marenich, A. V.; Bloino, J.; Janesko, B. G.; Gomperts, R.; Mennucci, B.; Hratch, D. J. Gaussian16. Gaussian, Inc.: Wallingford, CT 2016.
(18) Carpenter, J. E. Extension of Lewis Structure Concepts to Open-Shell and Excited-State Molecular Species, University of Wisconsin, Madison, WI, 1987.
(19) Lindsay, V. N. G.; Lin, W.; Charette, A. B. Experimental Evidence for the All-up Reactive Conformation of Chiral Rhodium(II) Carboxylate Catalysts: Enantioselective Synthesis of Cis-Cyclopropane α-Amino Acids. *J Am Chem Soc* **2009**, *131* (45), 16383–16385. https://doi.org/10.1021/ja9044955.
(20) De Angelis, A.; Boruta, D. T.; Lubin, J. B.; Plampin, J. N.; Yap, G. P. A.; Fox, J. M. The Chiral Crown Conformation in Paddlewheel Complexes. *Chemical Communications* **2010**, *46* (25), 4541–4543. https://doi.org/10.1039/c001557a.
(21) Adly, F. G. On the Structure of Chiral Dirhodium(II) Carboxylate Catalysts: Stereoselectivity Relevance and Insights for Improved Performance of Viable Whole-Cell Baeyer-Villiger Monoxygenase by Immobilization. *Catalysts* **2017**, *7* (11). https://doi.org/10.3390/catal7110347.
(22) Hansen, J.; Davies, H. M. L. High Symmetry Dirhodium(II) Paddlewheel Complexes as Chiral Catalysts. *Coord Chem Rev* **2008**, *252* (5–7), 545–555. https://doi.org/10.1016/j.ccr.2007.08.019.
(23) Guzei, I. A.; Wendt, M. An Improved Method for the Computation of Ligand Steric Effects Based on Solid Angles. *Journal of the Chemical Society. Dalton Transactions* **2006**, No. 33, 3991–3999. https://doi.org/10.1039/b605102b.
(24) Kirmse, W. Stable Singlet Carbenes - Plentiful and Versatile. *Angewandte Chemie - International Edition* **2004**, *43* (14), 1767–1769. https://doi.org/10.1002/anie.200301729.
(25) Verloop, A. *The Sterimol Approach: Further Development of the Method and New Applications*; International Union of Pure and Applied Chemistry, 1983. https://doi.org/10.1016/b978-0-08-029222-9.50051-2.
(26) Brethomé, A. V.; Fletcher, S. P.; Paton, R. S. Conformational Effects on Physical-Organic Descriptors: The Case of Sterimol Steric Parameters. *ACS Catal* **2019**, *9* (3), 2313–2323. https://doi.org/10.1021/acscatal.8b04043.
(27) Ouyang, R.; Curtarolo, S.; Ahmetcik, E.; Scheffler, M.; Ghiringhelli, L. M. SISSO: A Compressed-Sensing Method for Identifying the Best Low-Dimensional Descriptor in an Immensity of Offered Candidates. *Phys Rev Mater* **2018**, *2* (8), 1–11. https://doi.org/10.1103/PhysRevMaterials.2.083802.

(28) Wei, A.; Ye, H.; Guo, Z.; Xiong, J. SISSO-Assisted Prediction and Design of Mechanical Properties of Porous Graphene with a Uniform Nanopore Array. *Nanoscale Adv* **2022**, *4* (5), 1455–1463. https://doi.org/10.1039/d1na00457c.
(29) Guo, Z.; Hu, S.; Han, Z. K.; Ouyang, R. Improving Symbolic Regression for Predicting Materials Properties with Iterative Variable Selection. *J Chem Theory Comput* **2022**, *18* (8), 4945–4951. https://doi.org/10.1021/acs.jctc.2c00281.

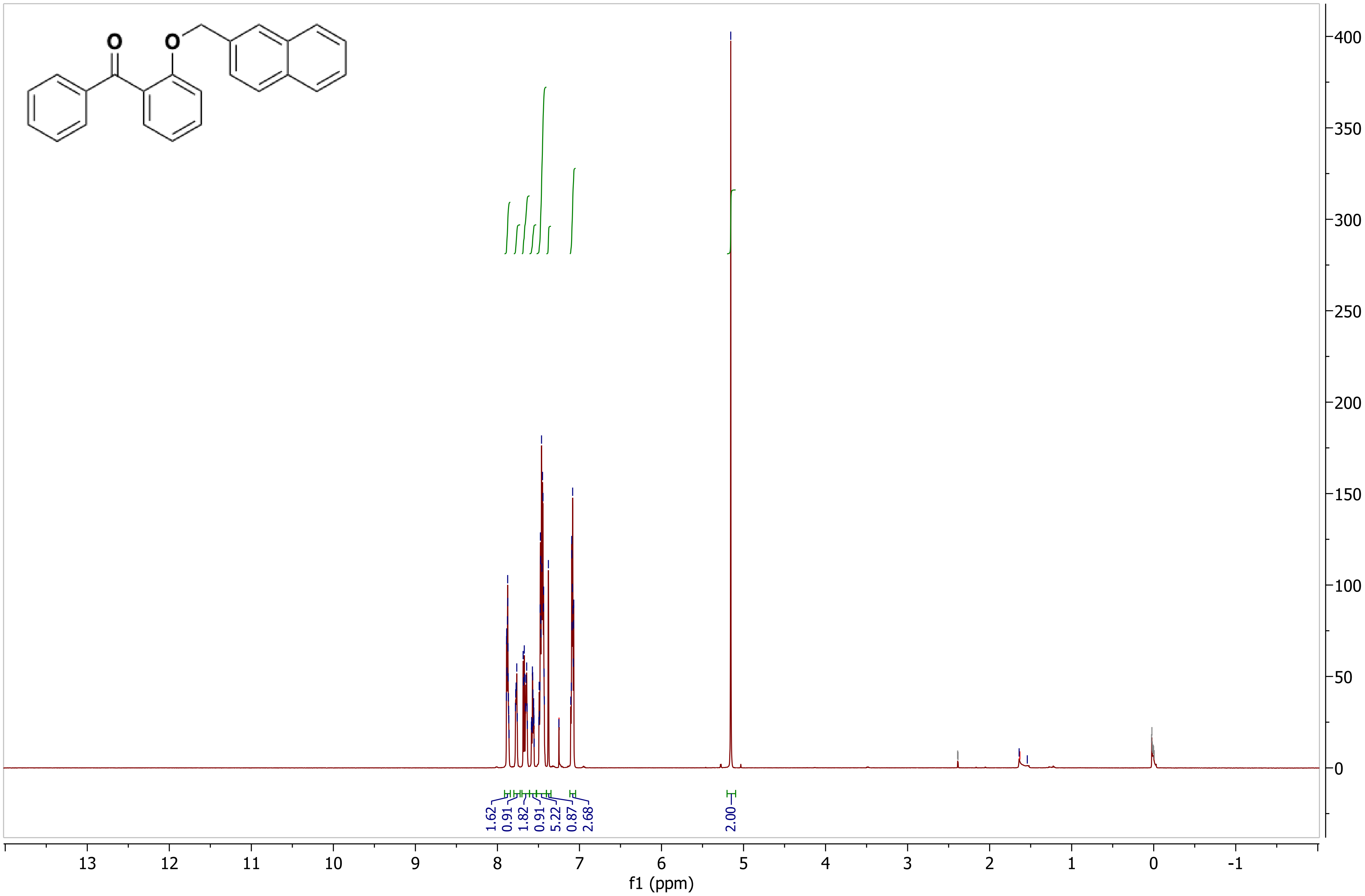

1.62
0.91
1.82
0.91
5.22
0.87
2.68
2.00
f1 (ppm)

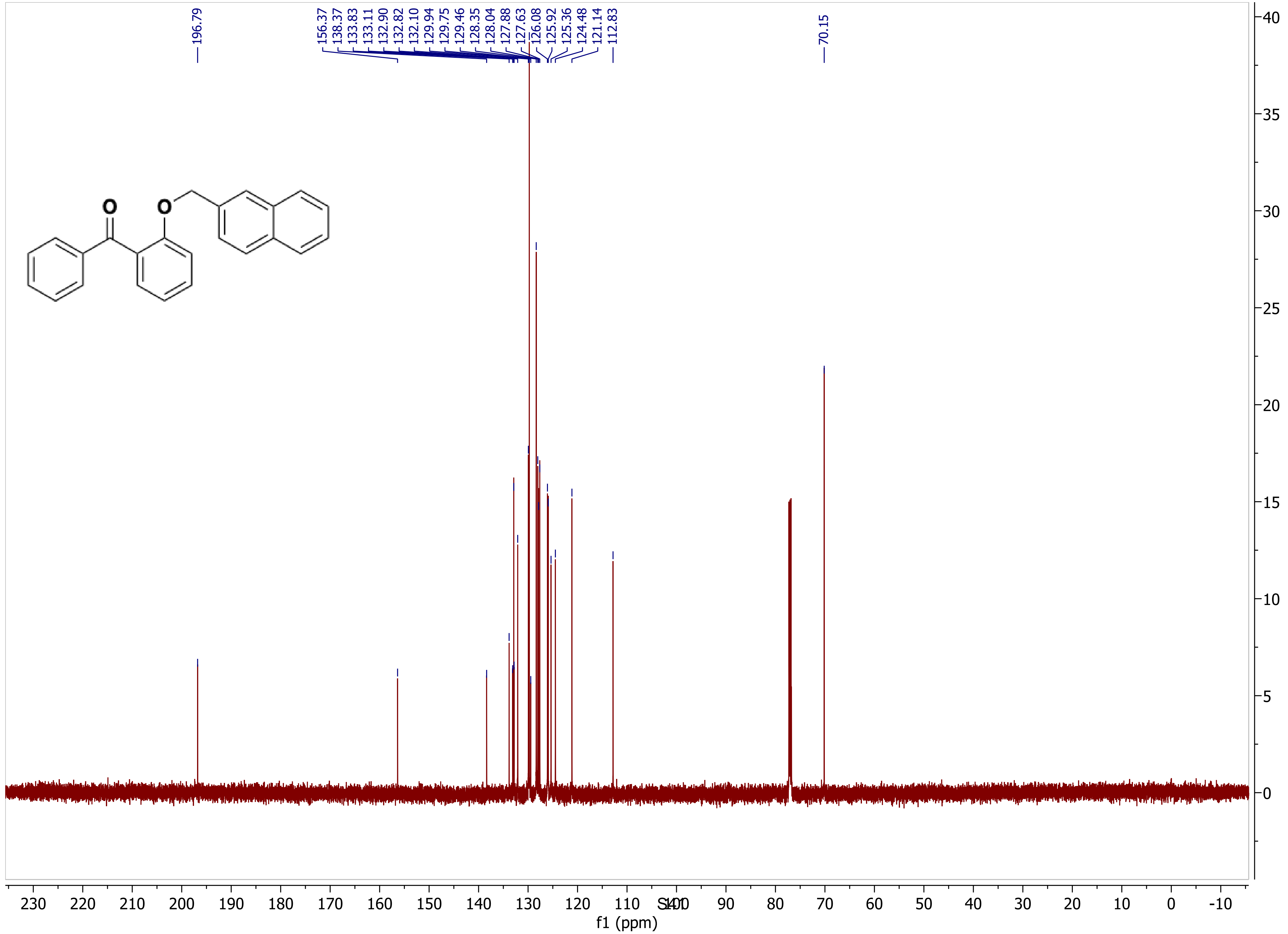
196.79
156.37
138.37
133.83
133.11
132.90
132.82
132.10
129.94
129.75
129.46
128.35
128.04
127.88
127.63
126.08
125.92
125.36
124.48
121.14
112.83
70.15
O
O
f1 (ppm)

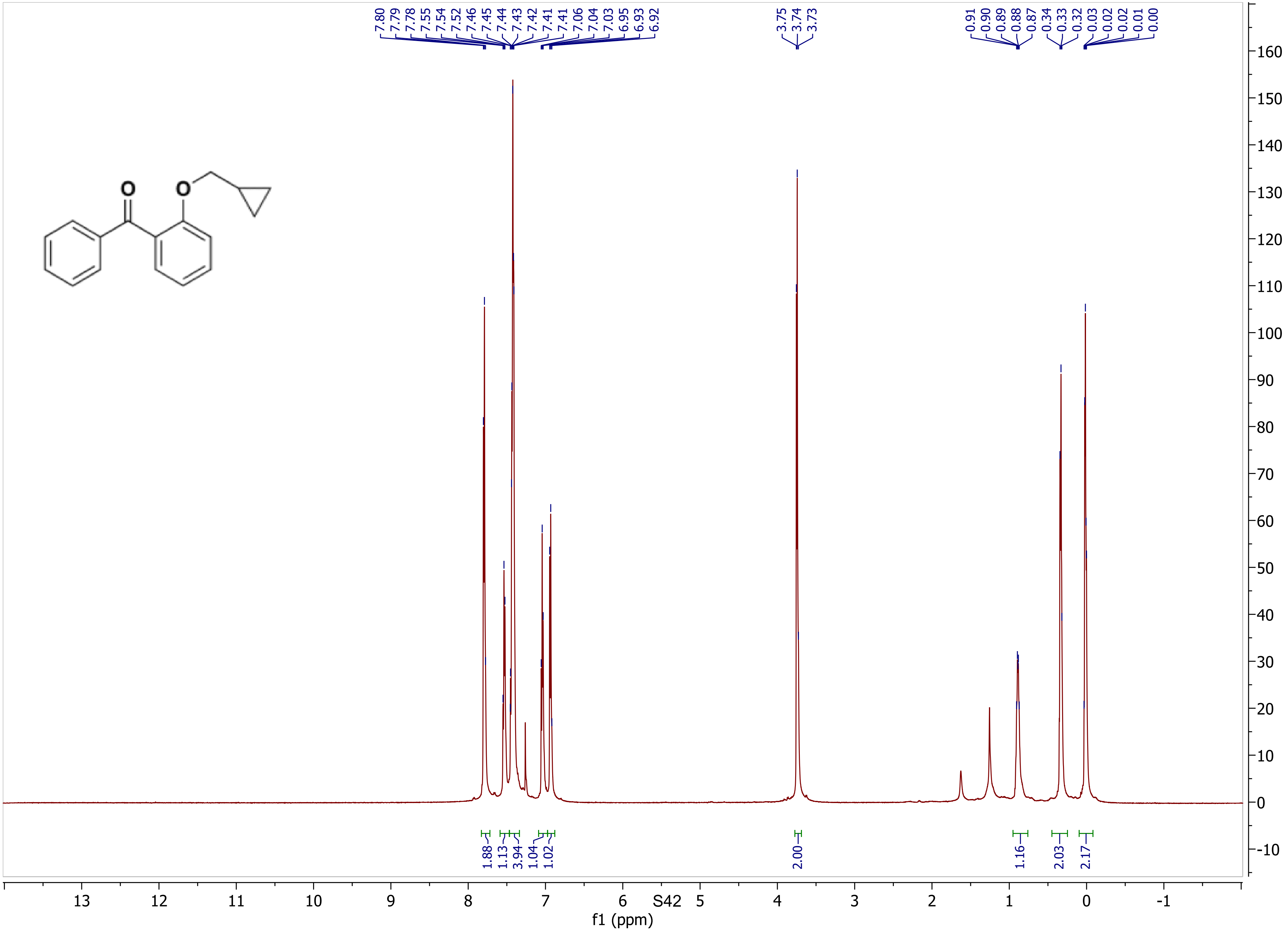
7.80
7.79
7.78
7.55
7.54
7.52
7.46
7.45
7.44
7.43
7.42
7.41
7.41
7.06
7.04
7.03
6.95
6.93
6.92
3.75
3.74
3.73
0.91
0.90
0.89
0.88
0.87
0.34
0.33
0.32
0.03
0.02
0.02
0.01
0.00
1.88
1.13
3.94
1.04
1.02
2.00
1.16
2.03
2.17
f1 (ppm)

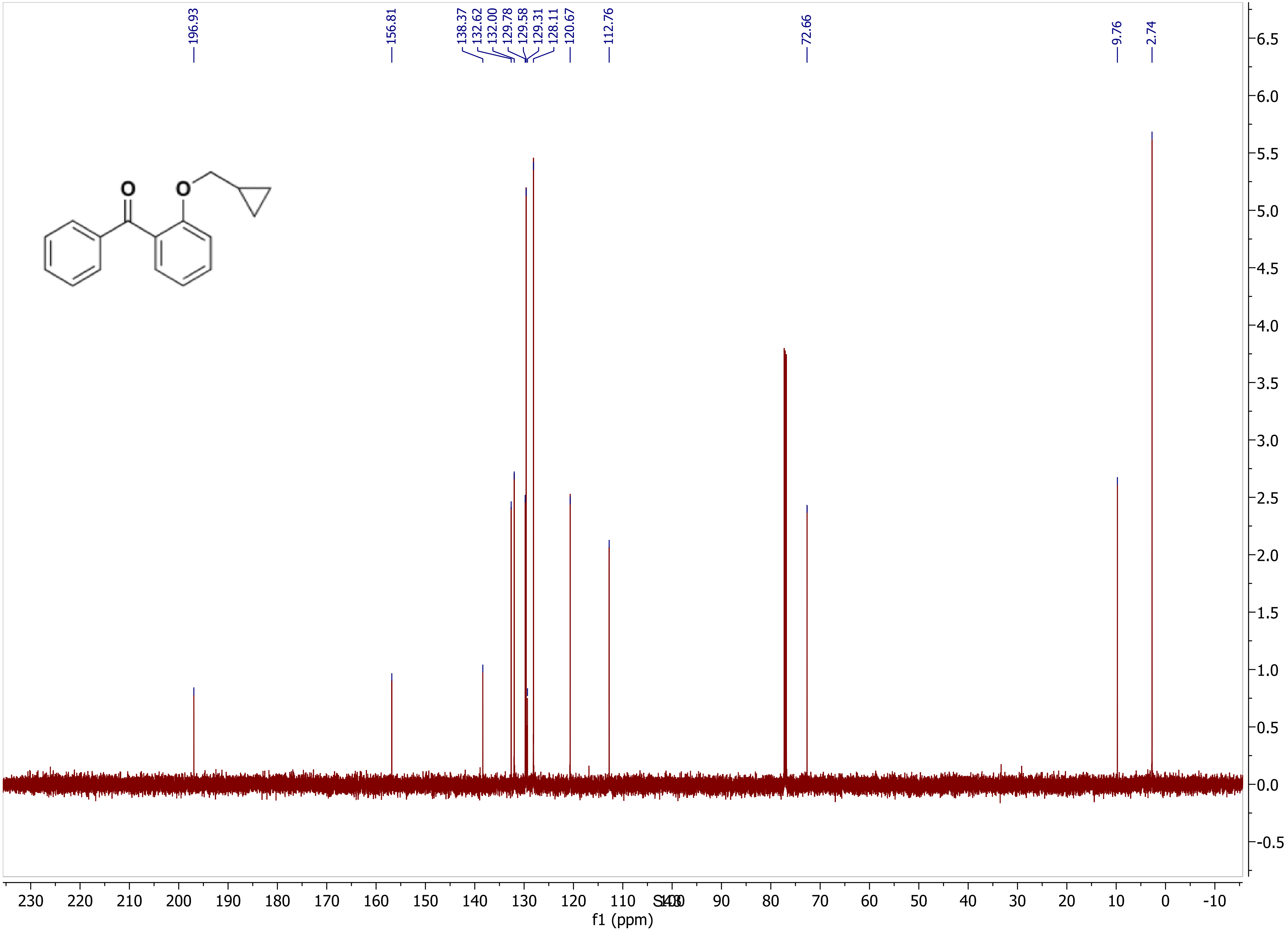
196.93
156.81
138.37
132.62
132.00
129.78
129.58
129.31
128.11
120.67
112.76
72.66
9.76
2.74
O
O
230 220 210 200 190 180 170 160 150 140 130 120 110 90 80 70 60 50 40 30 20 10 0 -10
f1 (ppm)
6.5 6.0 5.5 5.0 4.5 4.0 3.5 3.0 2.5 2.0 1.5 1.0 0.5 0.0 -0.5

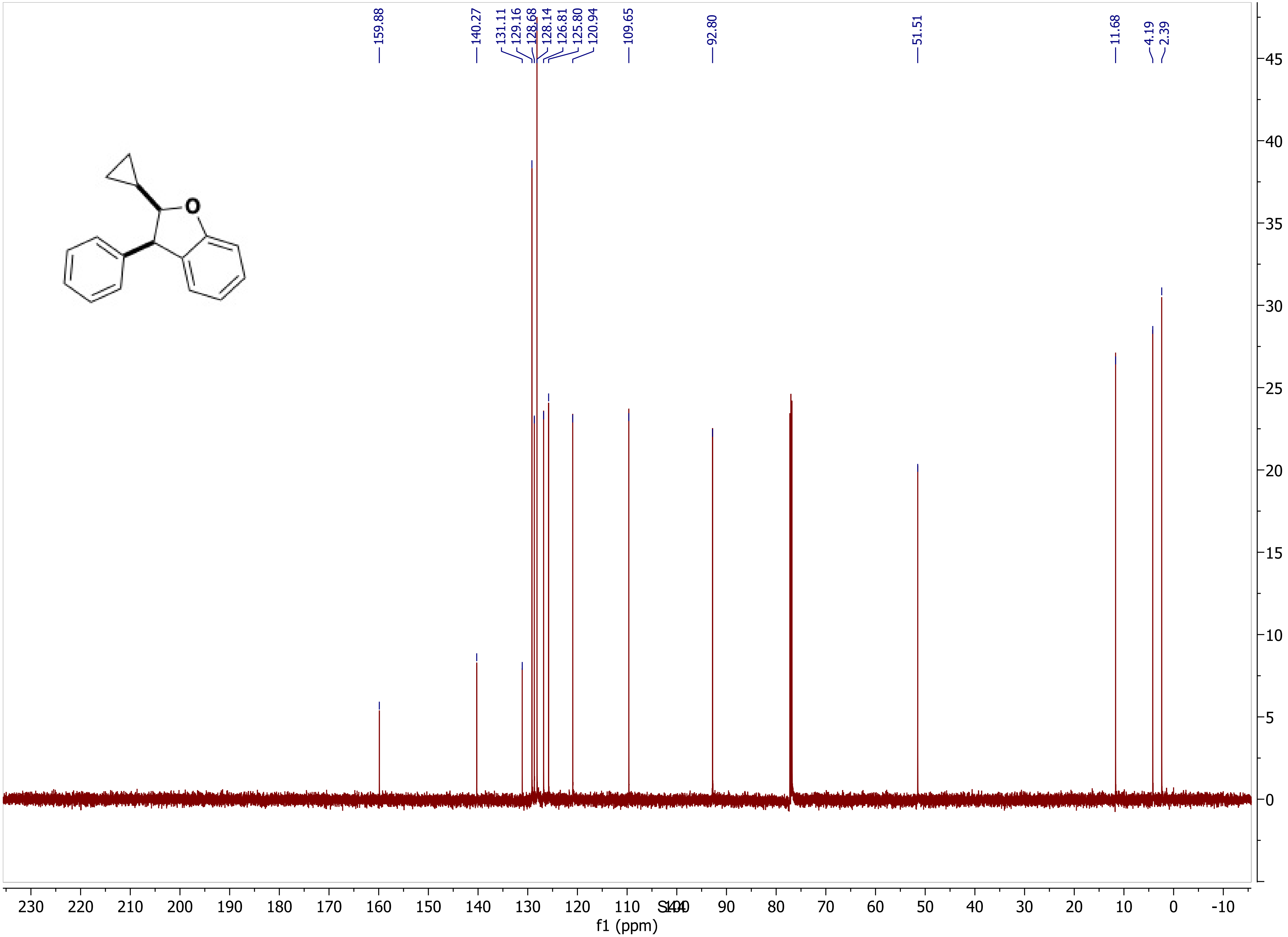
159.88
140.27
131.11
129.16
128.68
128.14
126.81
125.80
120.94
109.65
92.80
51.51
11.68
4.19
2.39
O
f1 (ppm)

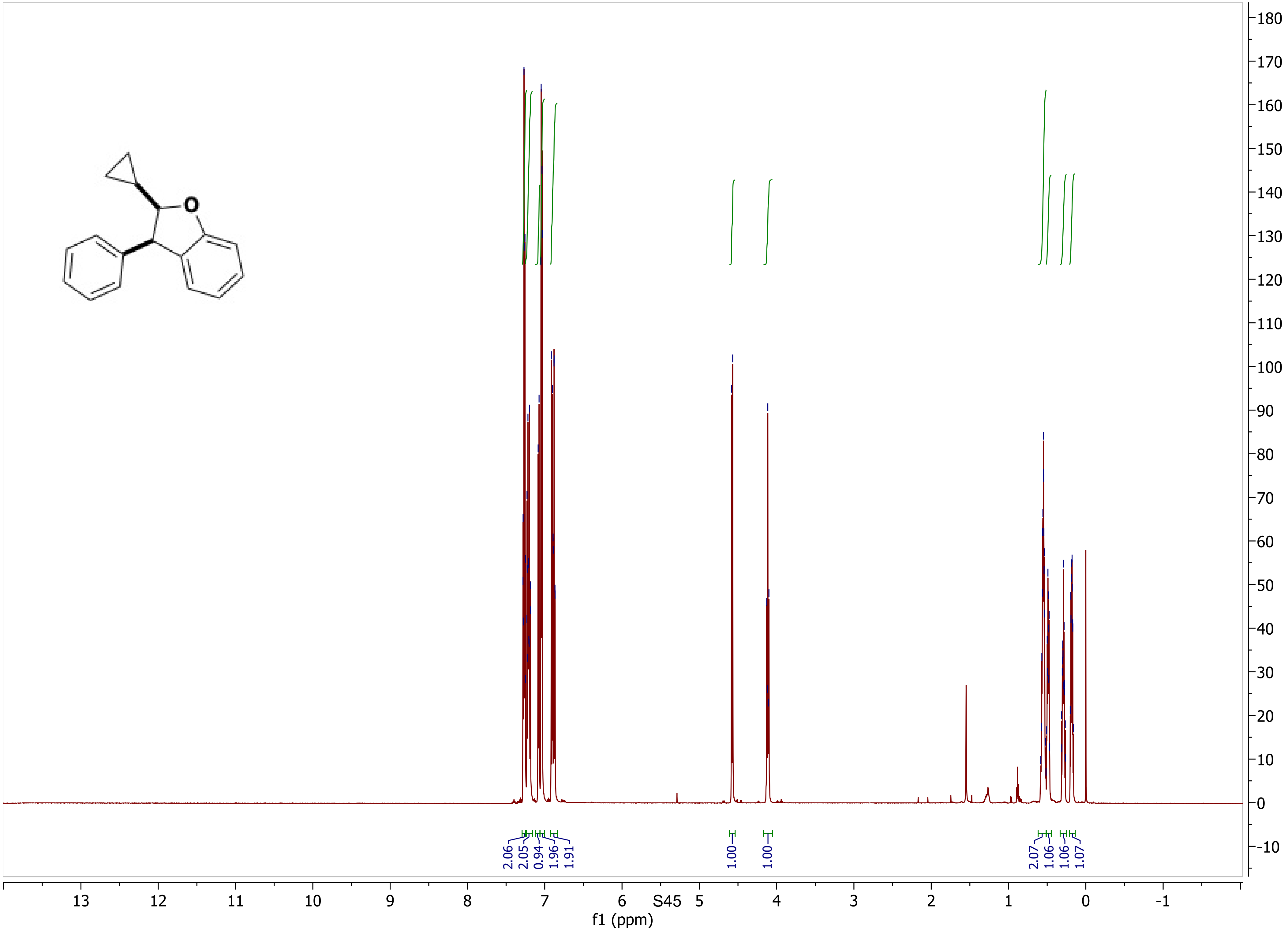
2.06
2.05
0.94
1.96
1.91
1.00
1.00
2.07
1.06
1.06
1.07
13 12 11 10 9 8 7 6 5 4 3 2 1 0 -1
f1 (ppm)
180 170 160 150 140 130 120 110 100 90 80 70 60 50 40 30 20 10 0 -10

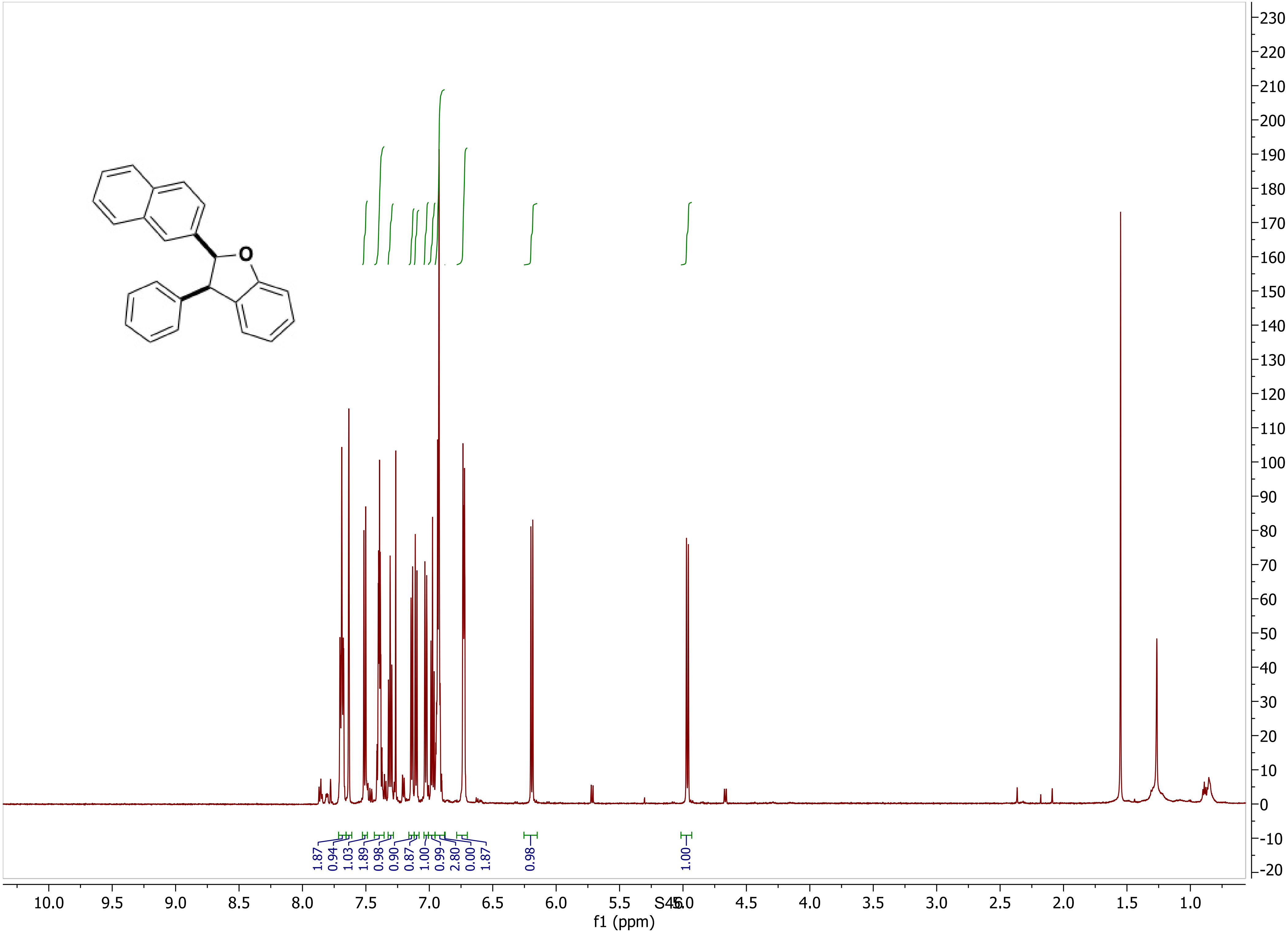

1.87
0.94
1.03
1.89
0.98
0.90
0.87
1.00
0.99
2.80
0.00
1.87
0.98
1.00
10.0 9.5 9.0 8.5 8.0 7.5 7.0 6.5 6.0 5.5 5.0 4.5 4.0 3.5 3.0 2.5 2.0 1.5 1.0
f1 (ppm)
230 220 210 200 190 180 170 160 150 140 130 120 110 100 90 80 70 60 50 40 30 20 10 0 -10 -20

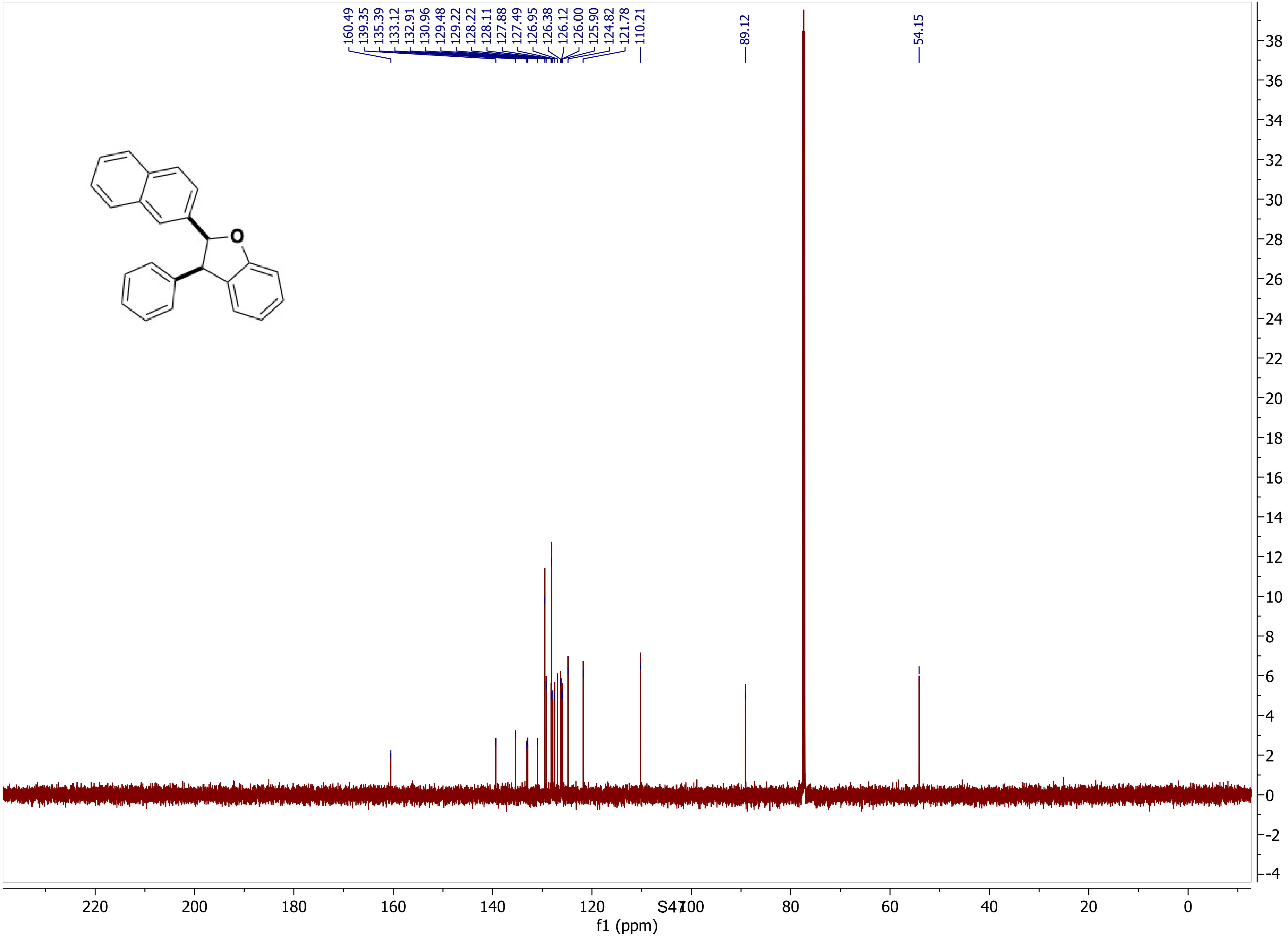
160.49
139.35
135.39
133.12
132.91
130.96
129.48
129.22
128.22
128.11
127.88
127.49
126.95
126.38
126.12
126.00
125.90
124.82
121.78
110.21
89.12
54.15
O
f1 (ppm)

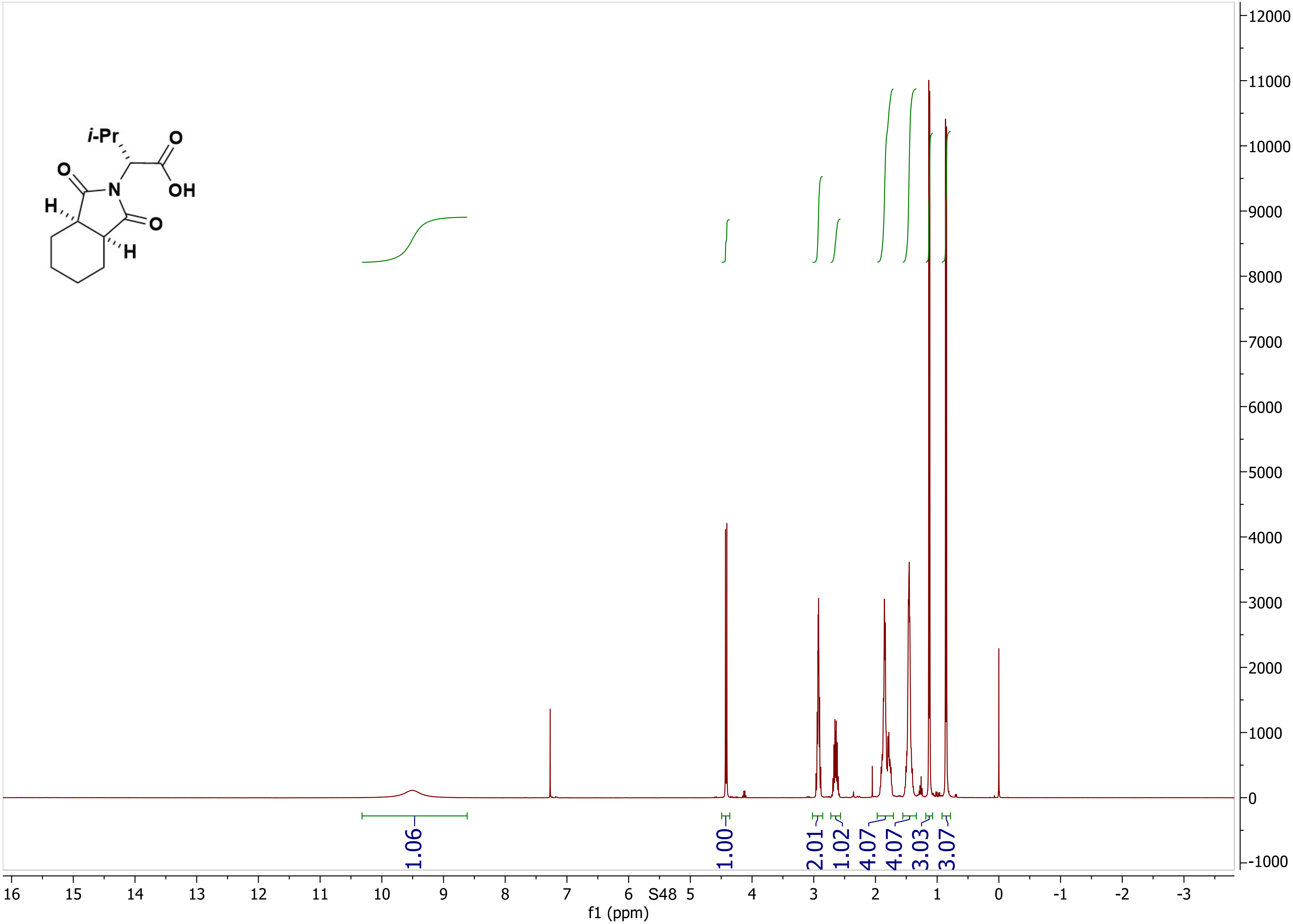
i-Pr
O
OH
N
O
H
O
H
1.06
1.00
2.01
1.02
4.07
4.07
3.03
3.07
16 15 14 13 12 11 10 9 8 7 6 5 4 3 2 1 0 -1 -2 -3
f1 (ppm)
12000 11000 10000 9000 8000 7000 6000 5000 4000 3000 2000 1000 0 -1000

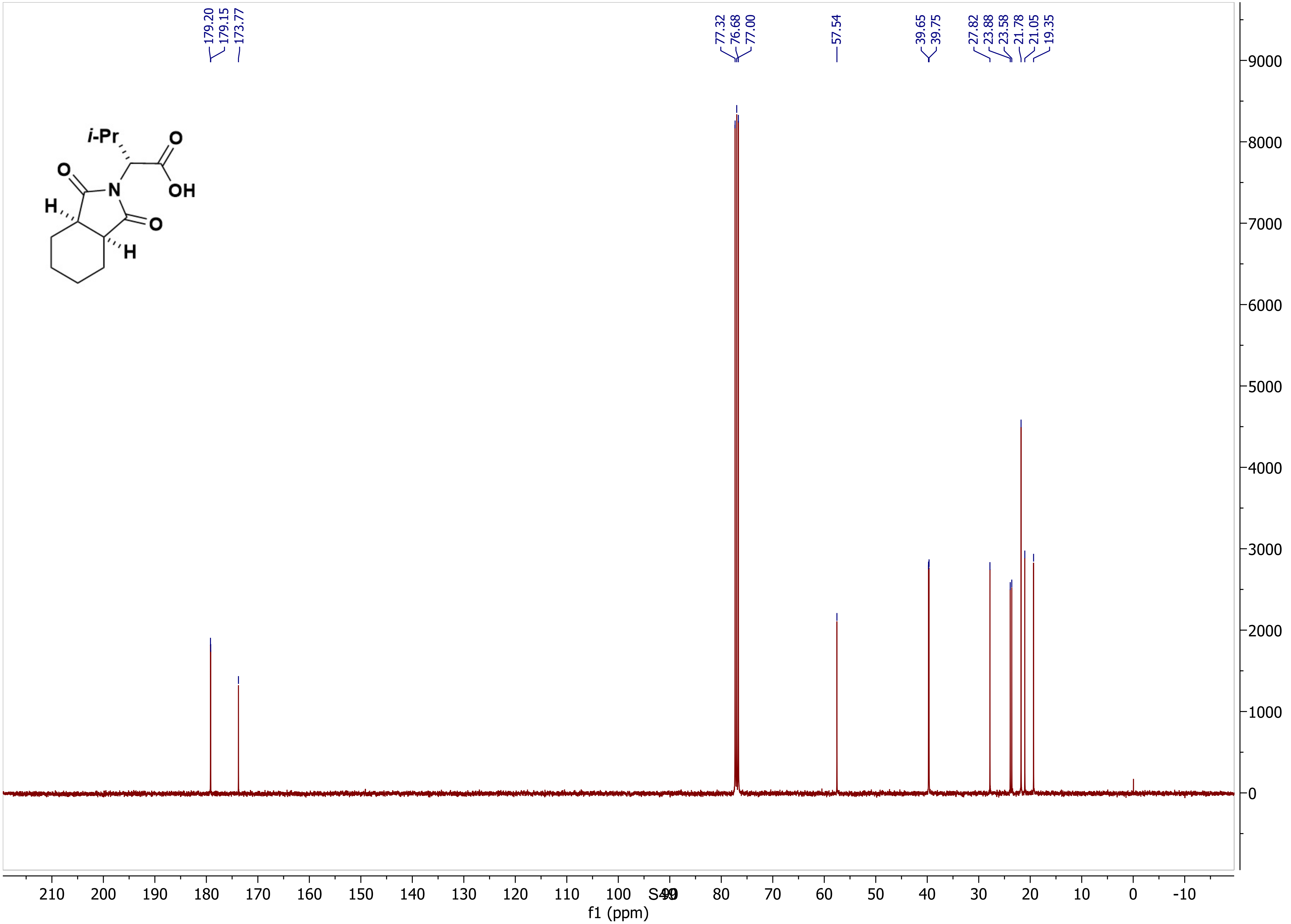
179.20
179.15
173.77
77.32
76.68
77.00
57.54
39.65
39.75
27.82
23.88
23.58
21.78
21.05
19.35
i-Pr
O
OH
N
H
O
O
H
9000
8000
7000
6000
5000
4000
3000
2000
1000
0
210 200 190 180 170 160 150 140 130 120 110 100 90 80 70 60 50 40 30 20 10 0 -10
f1 (ppm)

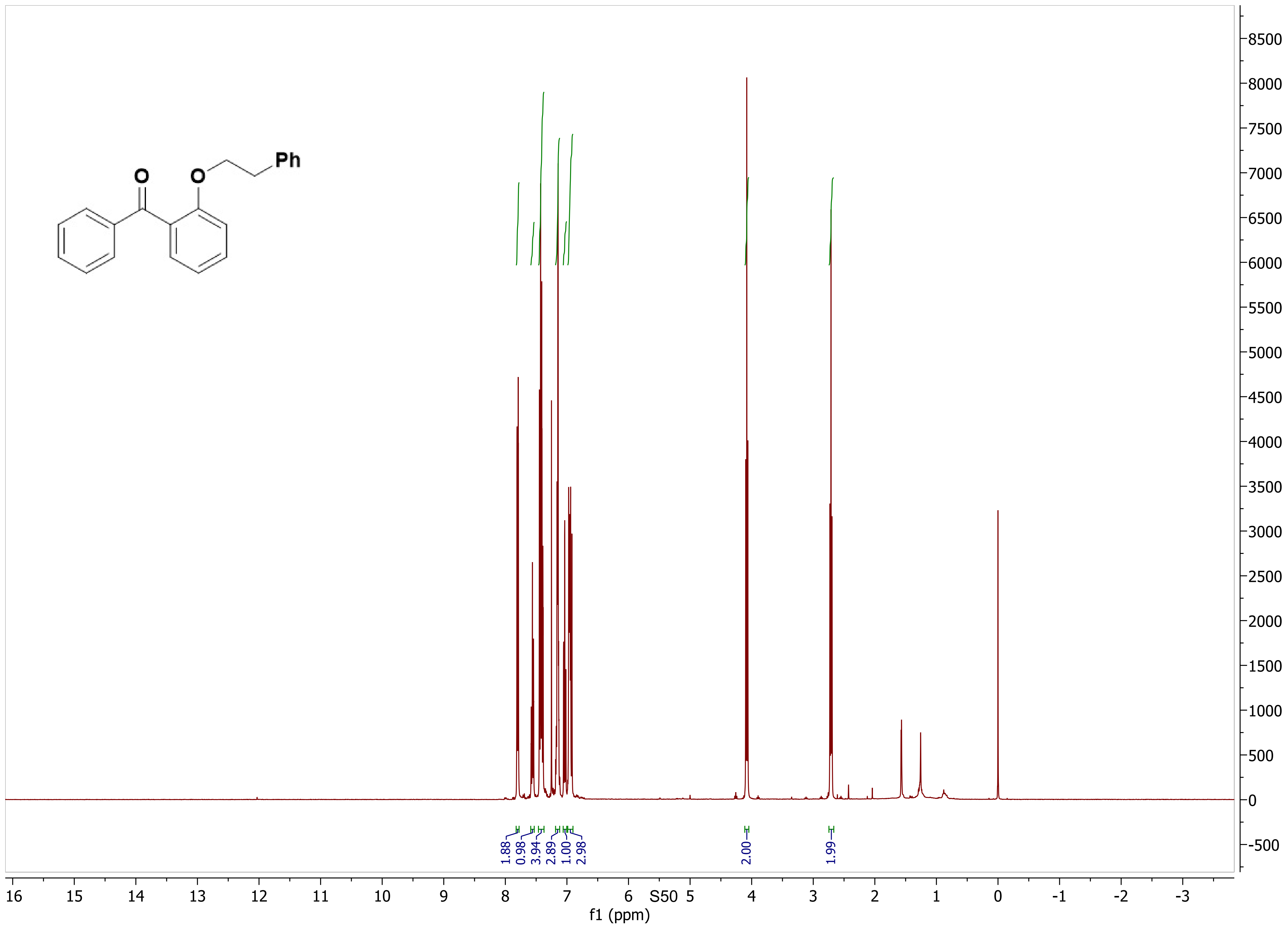
Ph
O
O
1.88
0.98
3.94
2.89
1.00
2.98
2.00
1.99
f1 (ppm)

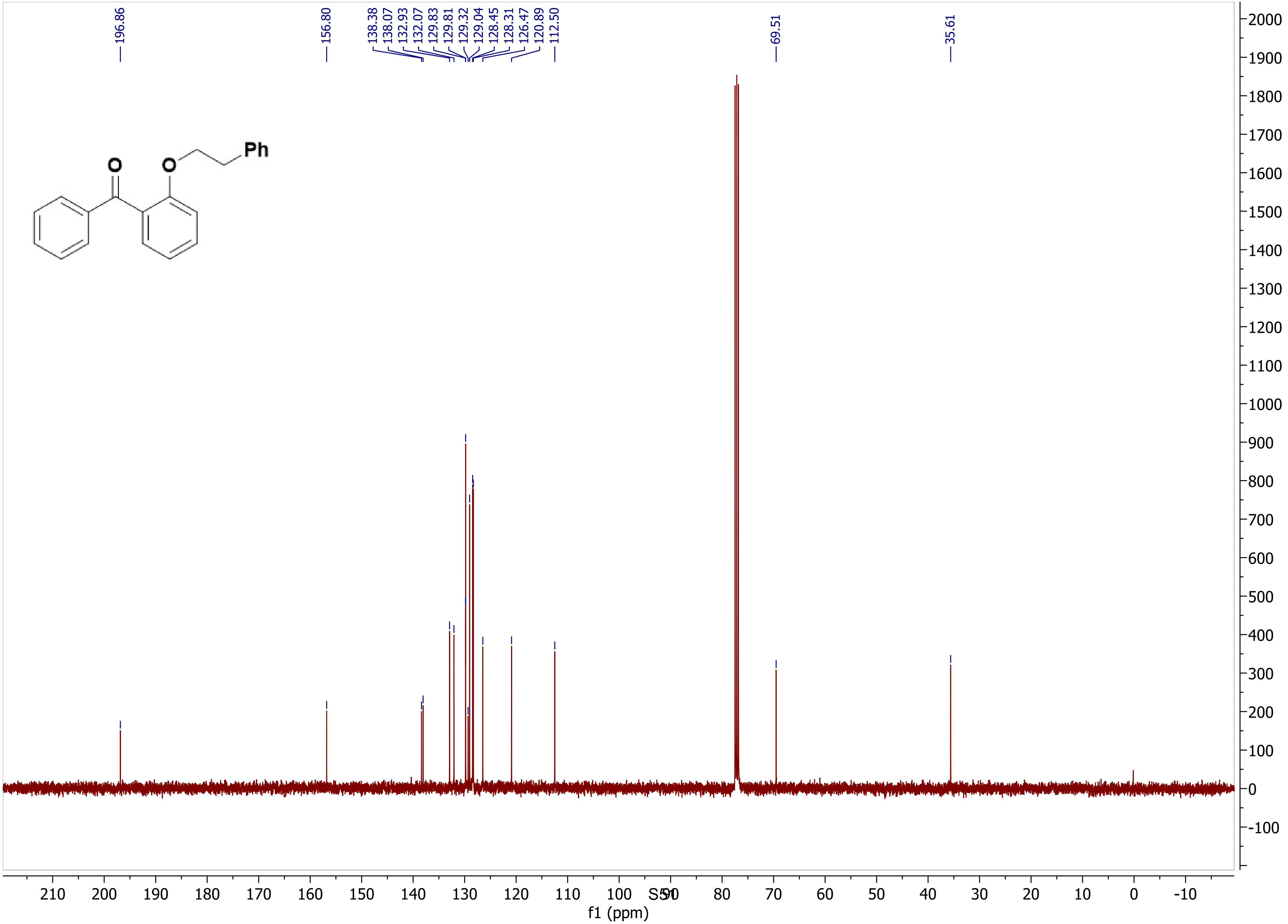
196.86
156.80
138.38
138.07
132.93
132.07
129.83
129.81
129.32
129.04
128.45
128.31
126.47
120.89
112.50
69.51
35.61
O
O
Ph
f1 (ppm)

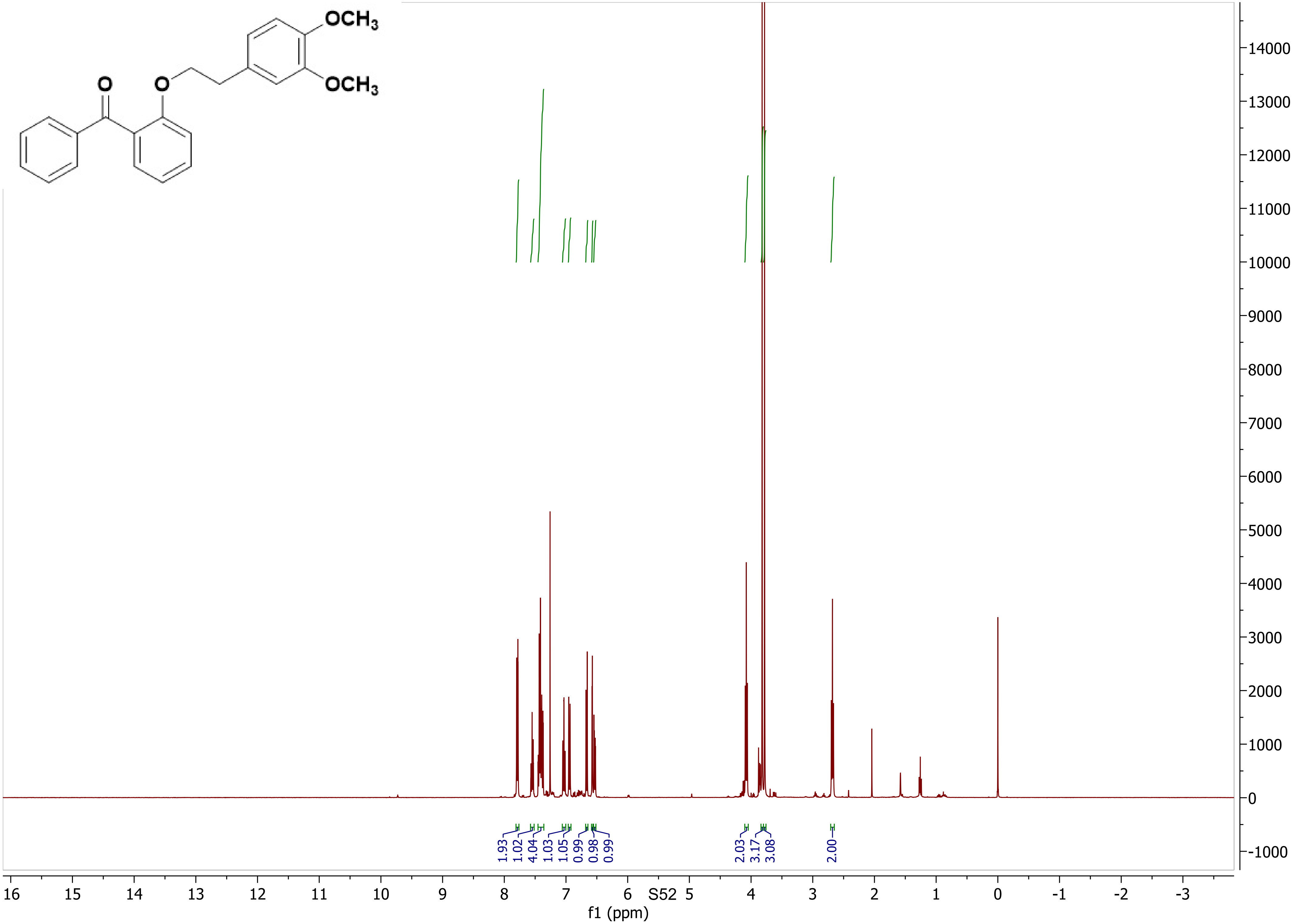
OCH3
OCH3
O
O
1.93
1.02
4.04
1.03
1.05
0.99
0.98
0.99
2.03
3.17
3.08
2.00
f1 (ppm)

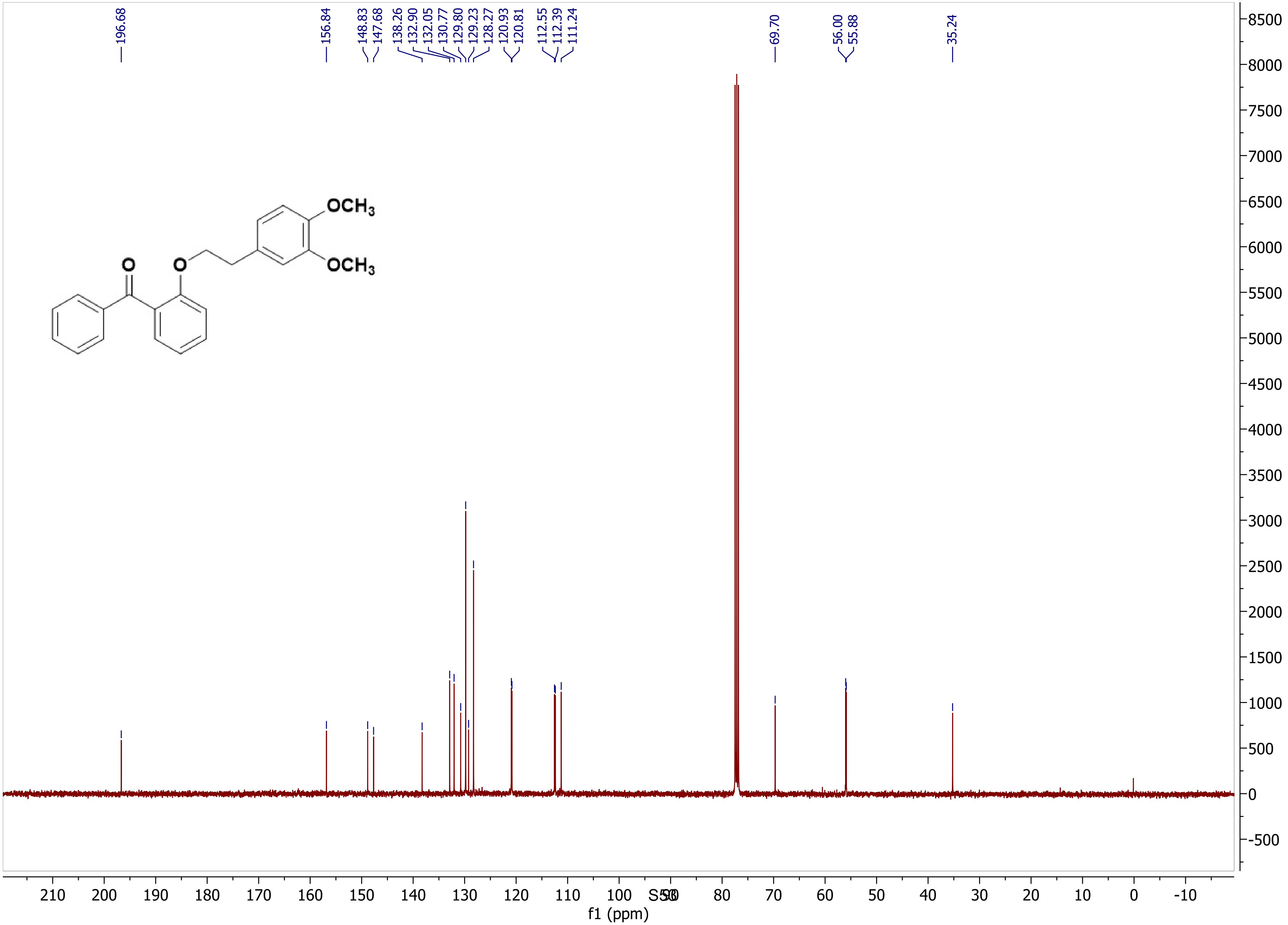
196.68
156.84
148.83
147.68
138.26
132.90
132.05
130.77
129.80
129.23
128.27
120.93
120.81
112.55
112.39
111.24
69.70
56.00
55.88
35.24
OCH3
OCH3
O
O
210 200 190 180 170 160 150 140 130 120 110 100 90 80 70 60 50 40 30 20 10 0 -10
f1 (ppm)
8500 8000 7500 7000 6500 6000 5500 5000 4500 4000 3500 3000 2500 2000 1500 1000 500 0 -500

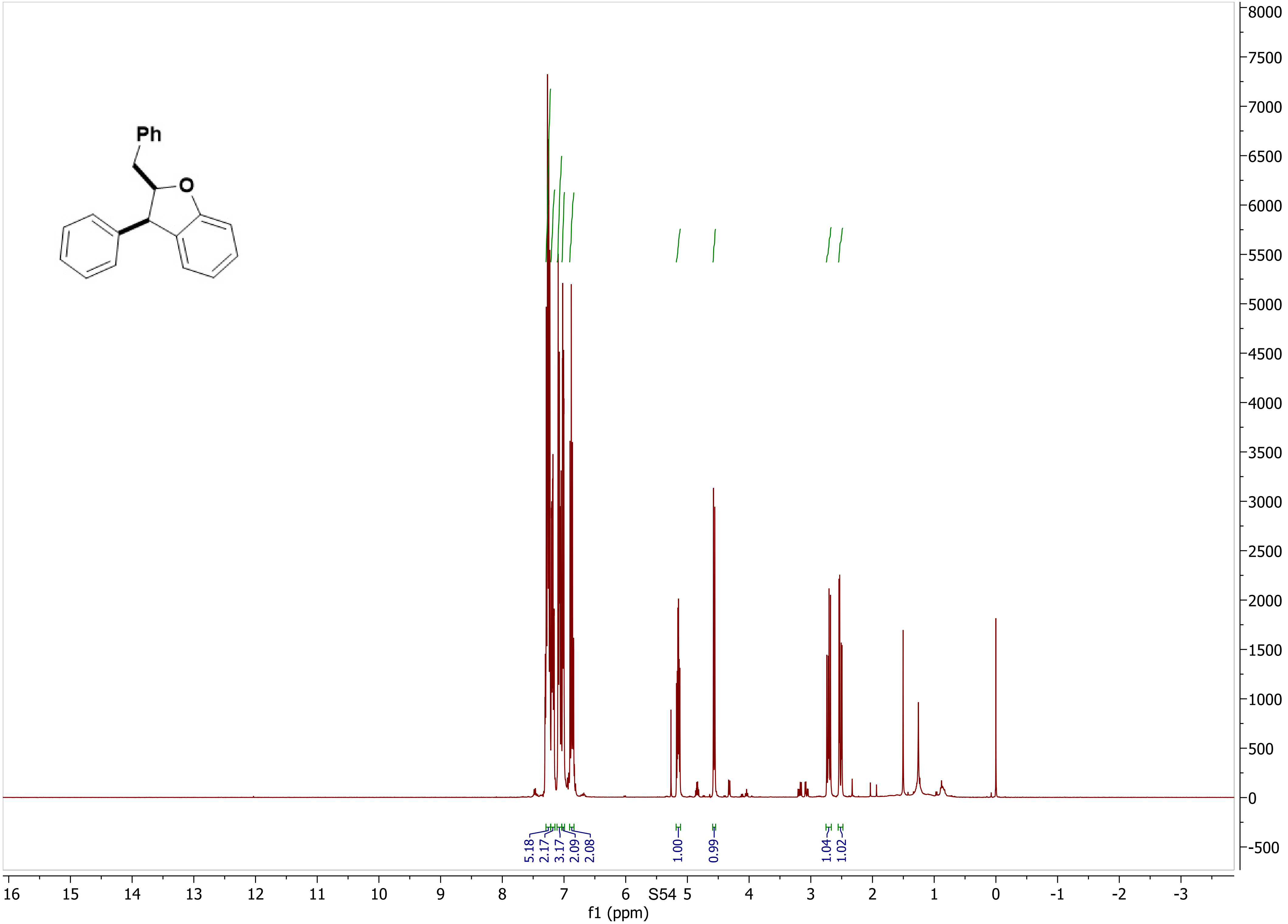
Ph
O
5.18
2.17
3.17
2.09
2.08
1.00
0.99
1.04
1.02
f1 (ppm)

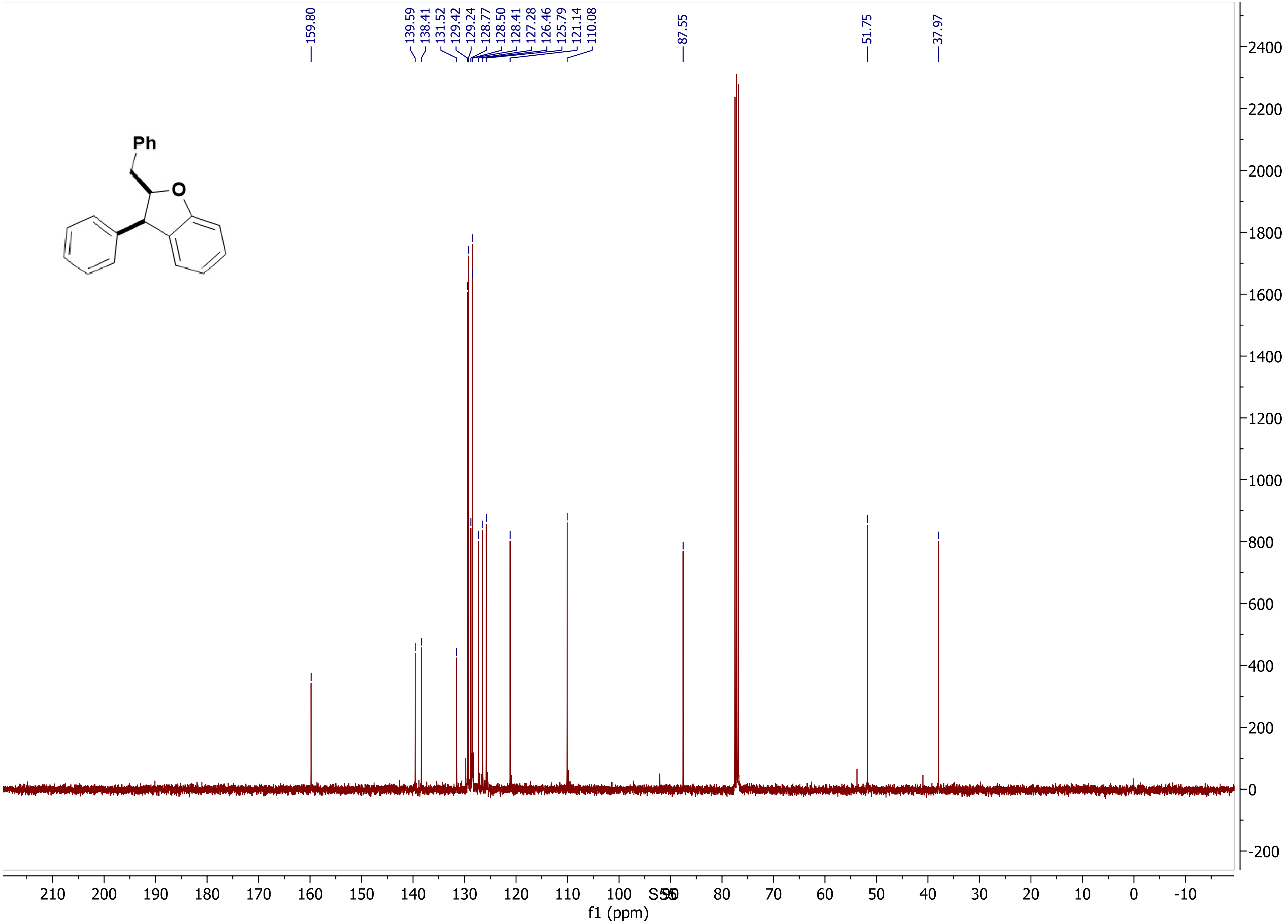
159.80
139.59
138.41
131.52
129.42
129.24
128.77
128.50
128.41
127.28
126.46
125.79
121.14
110.08
87.55
51.75
37.97
Ph
O
f1 (ppm)

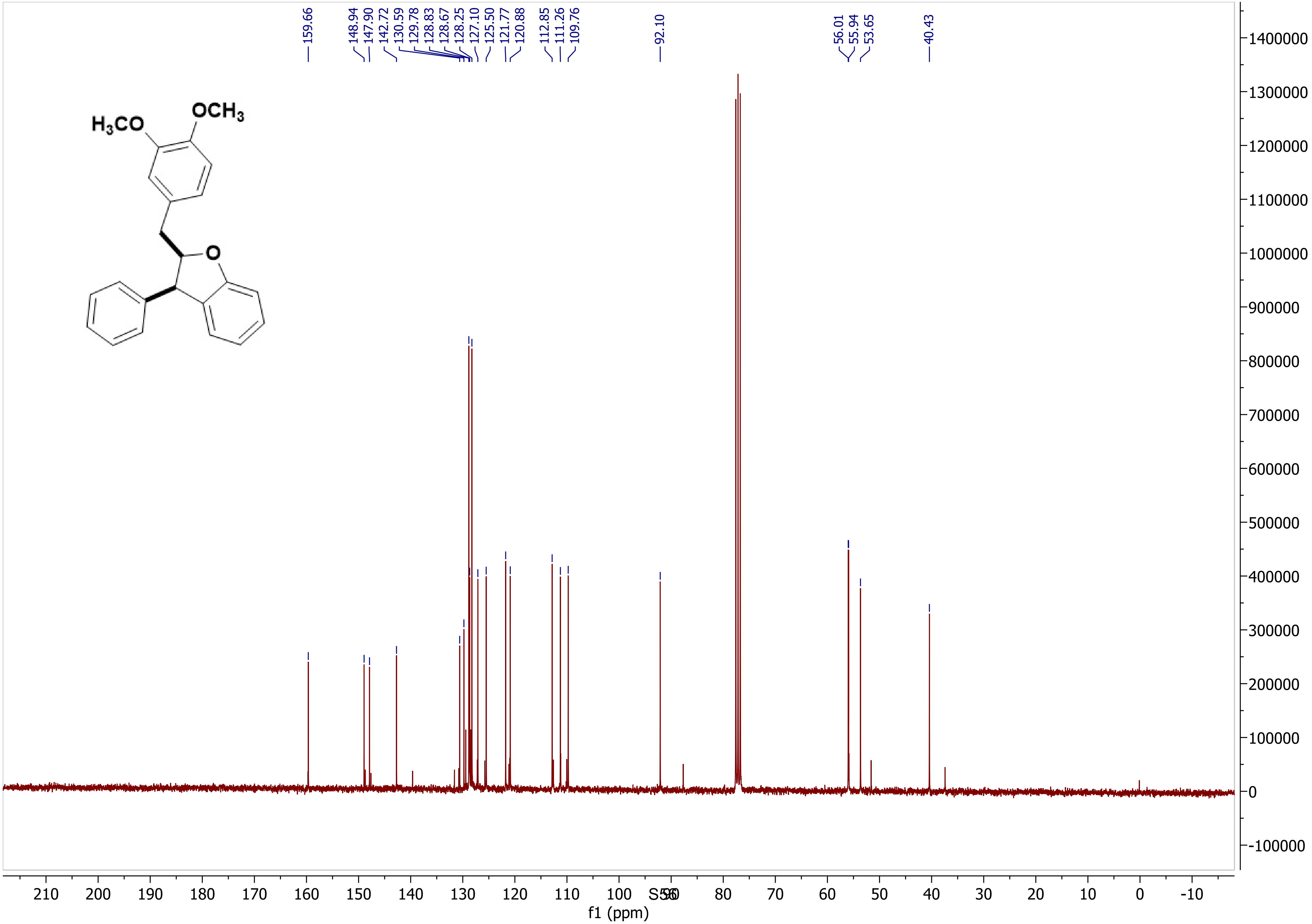
159.66
148.94
147.90
142.72
130.59
129.78
128.83
128.67
128.25
127.10
125.50
121.77
120.88
112.85
111.26
109.76
92.10
56.01
55.94
53.65
40.43
H_3CO
OCH_3
O
1400000
1300000
1200000
1100000
1000000
900000
800000
700000
600000
500000
400000
300000
200000
100000
0
-100000
210 200 190 180 170 160 150 140 130 120 110 100 90 80 70 60 50 40 30 20 10 0 -10
f1 (ppm)

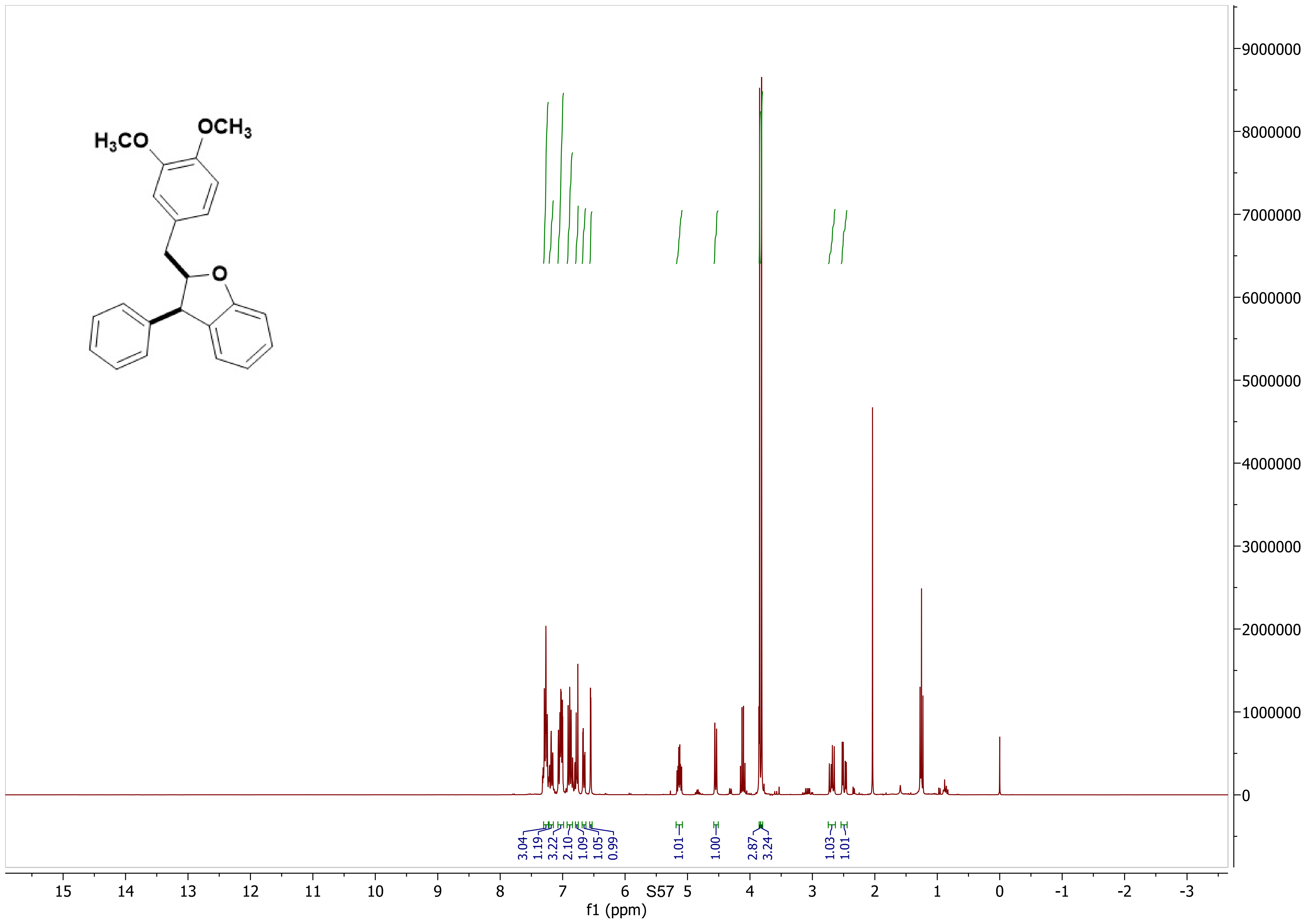

H3CO
OCH3
O
3.04
1.19
3.22
2.10
1.09
1.05
0.99
1.01
1.00
2.87
3.24
1.03
1.01
9000000
8000000
7000000
6000000
5000000
4000000
3000000
2000000
1000000
0
15
14
13
12
11
10
9
8
7
6
5
4
3
2
1
0
-1
-2
-3
f1 (ppm)

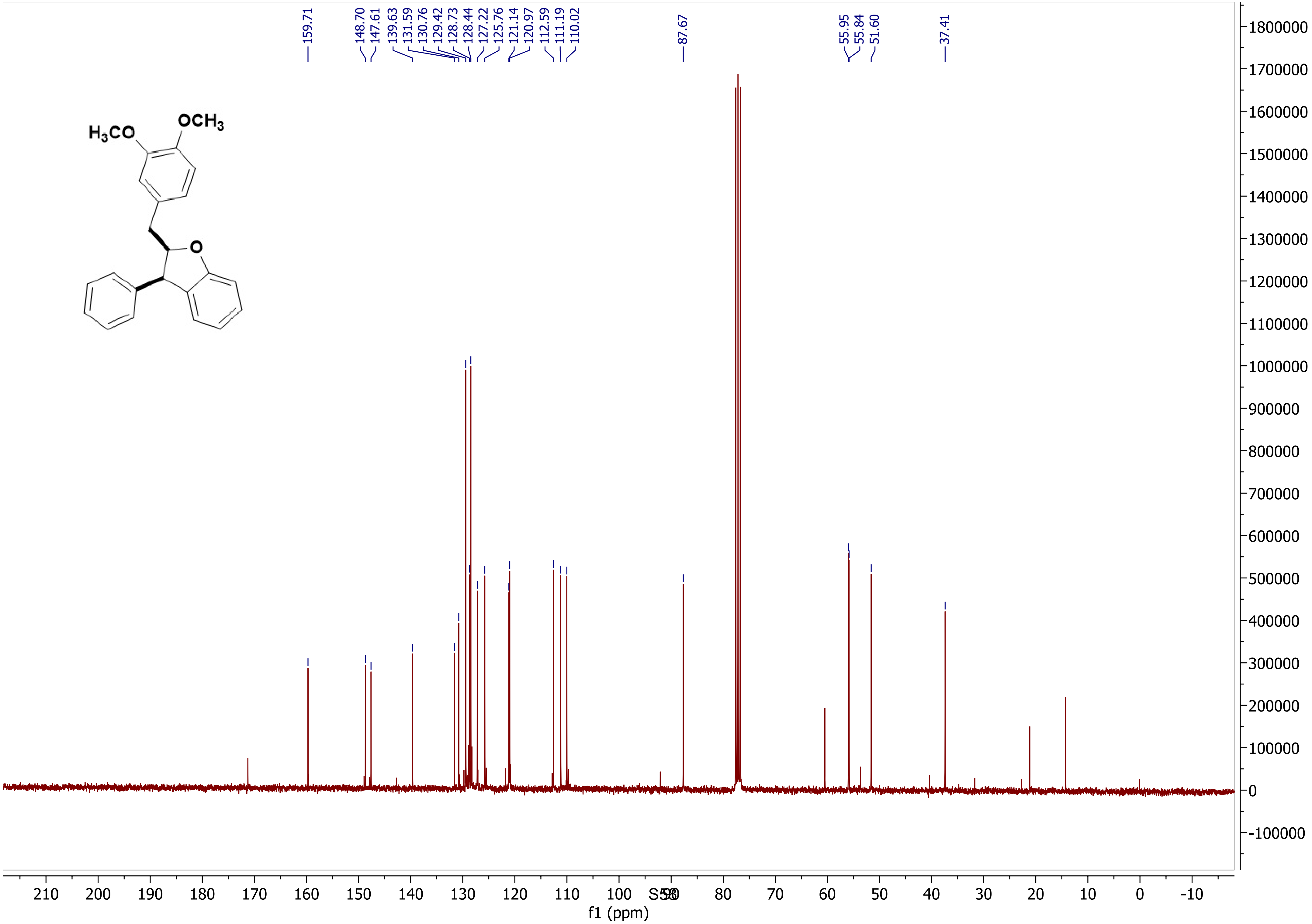

159.71
148.70
147.61
139.63
131.59
130.76
129.42
128.73
128.44
127.22
125.76
121.14
120.97
112.59
111.19
110.02
87.67
55.95
55.84
51.60
37.41
H_3CO
OCH_3
O
f1 (ppm)

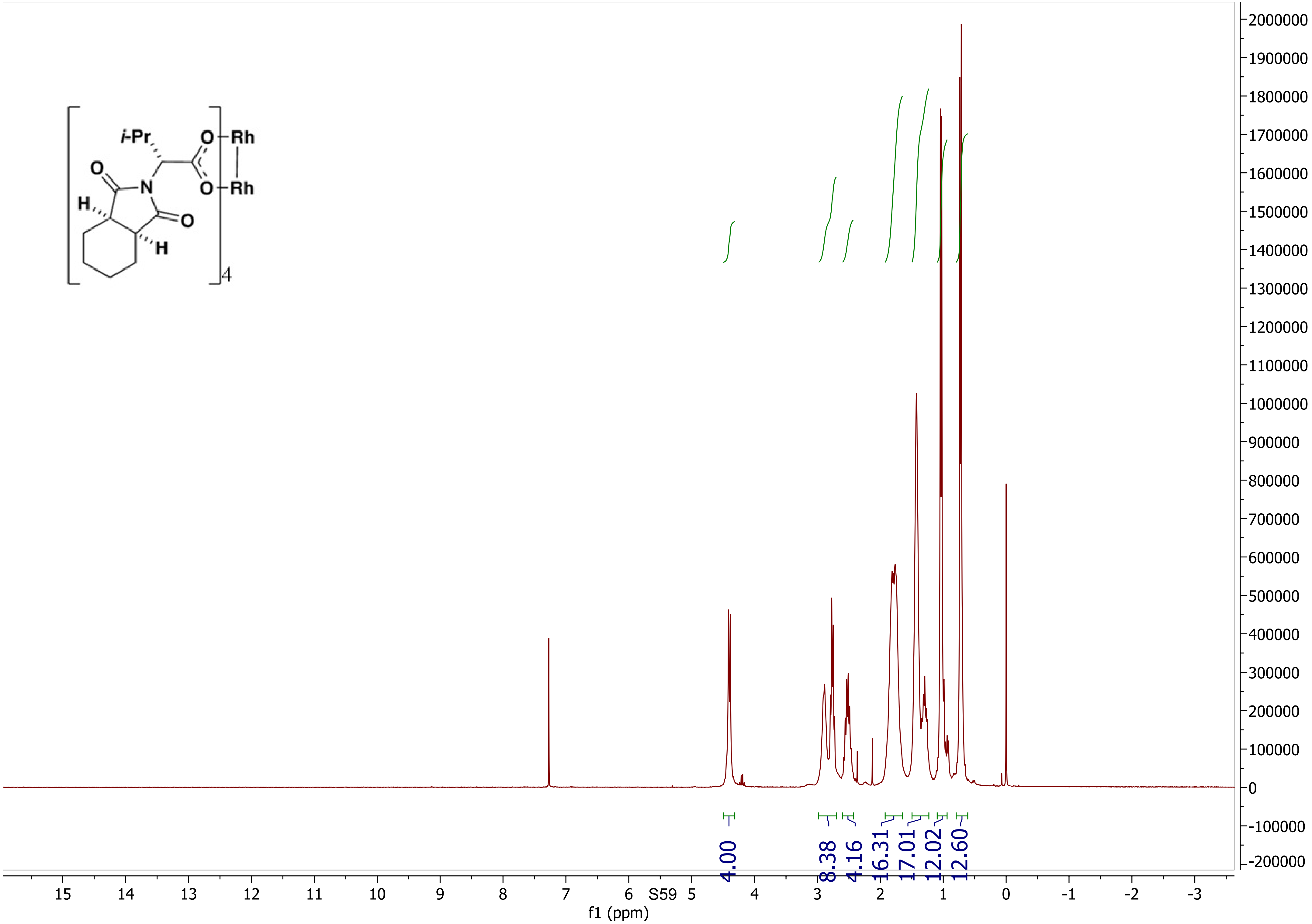
i-Pr
O
Rh
N
H
4
4.00
8.38
4.16
16.31
17.01
12.02
12.60
f1 (ppm)

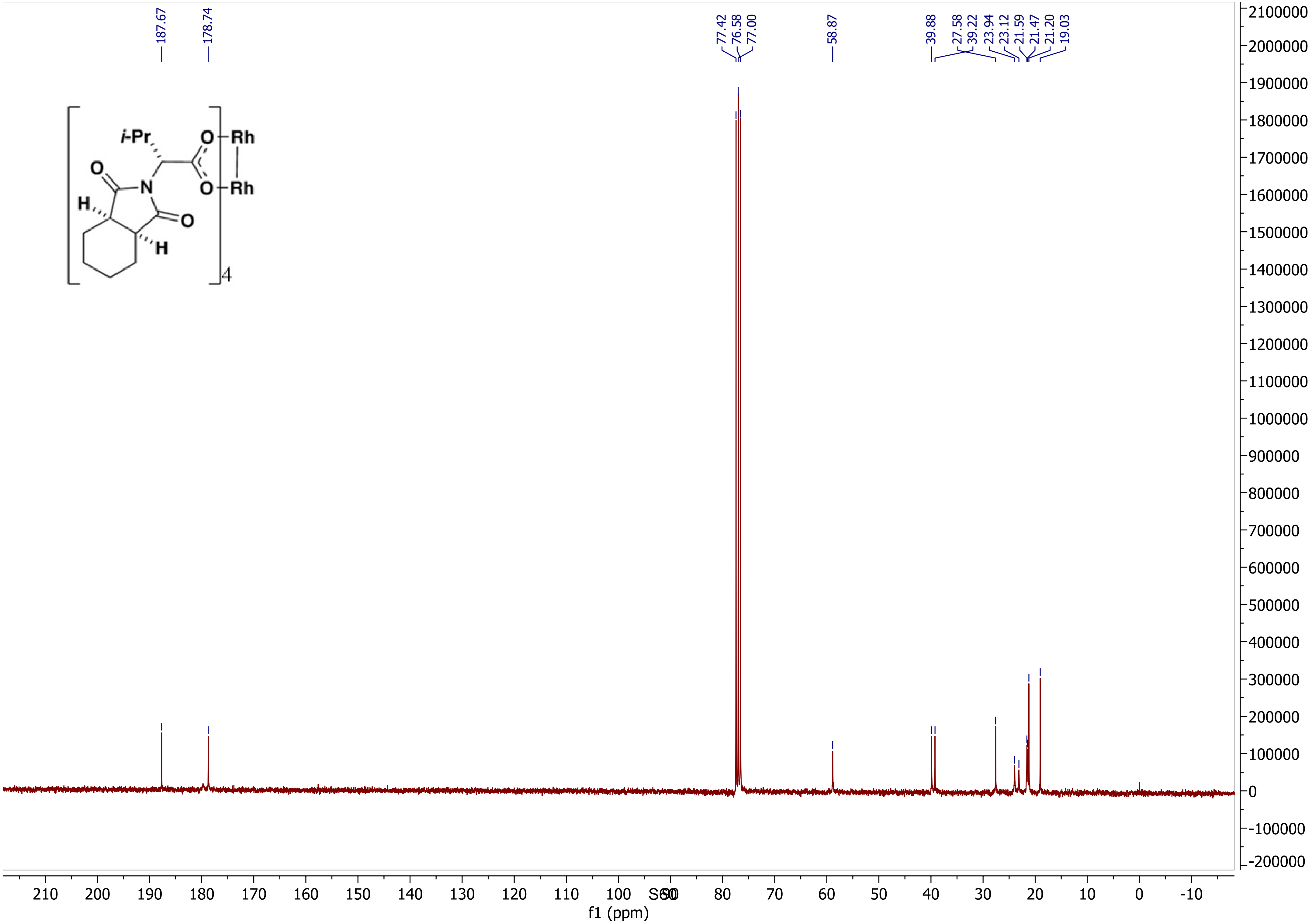
187.67
178.74
77.42
76.58
77.00
58.87
39.88
27.58
39.22
23.94
23.12
21.59
21.47
21.20
19.03
i-Pr
O
N
H
H
O
O
Rh
Rh
4
2100000
2000000
1900000
1800000
1700000
1600000
1500000
1400000
1300000
1200000
1100000
1000000
900000
800000
700000
600000
500000
400000
300000
200000
100000
0
-100000
-200000
210 200 190 180 170 160 150 140 130 120 110 100 90 80 70 60 50 40 30 20 10 0 -10
f1 (ppm)

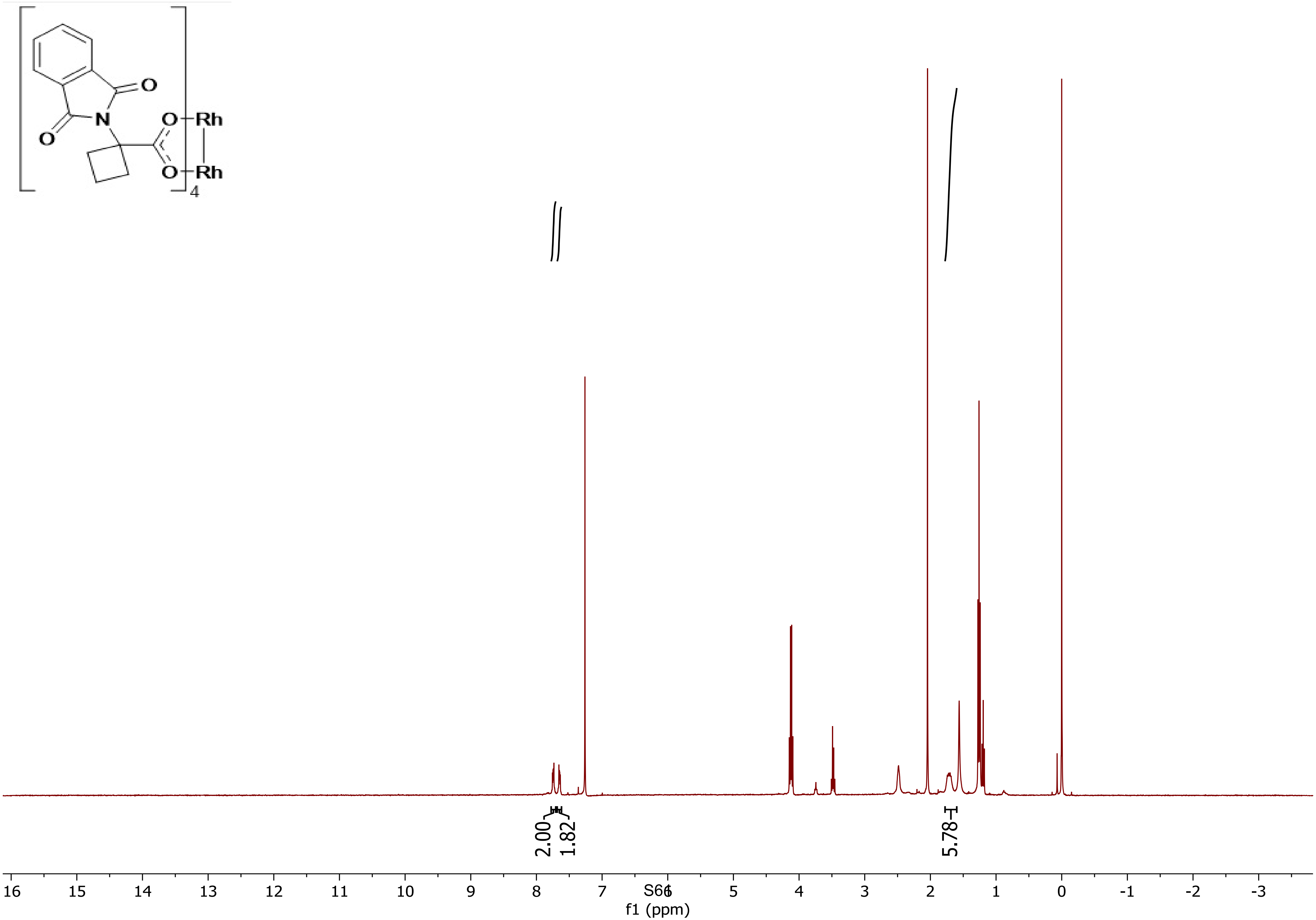
O
N
O
O
Rh
O
Rh
4
2.00
1.82
5.78
16
15
14
13
12
11
10
9
8
7
6
5
4
3
2
1
0
-1
-2
-3
f1 (ppm)

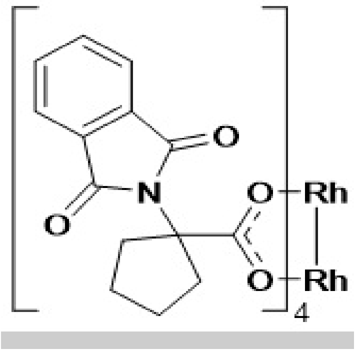
O
N
O
O
O
Rh
Rh
4

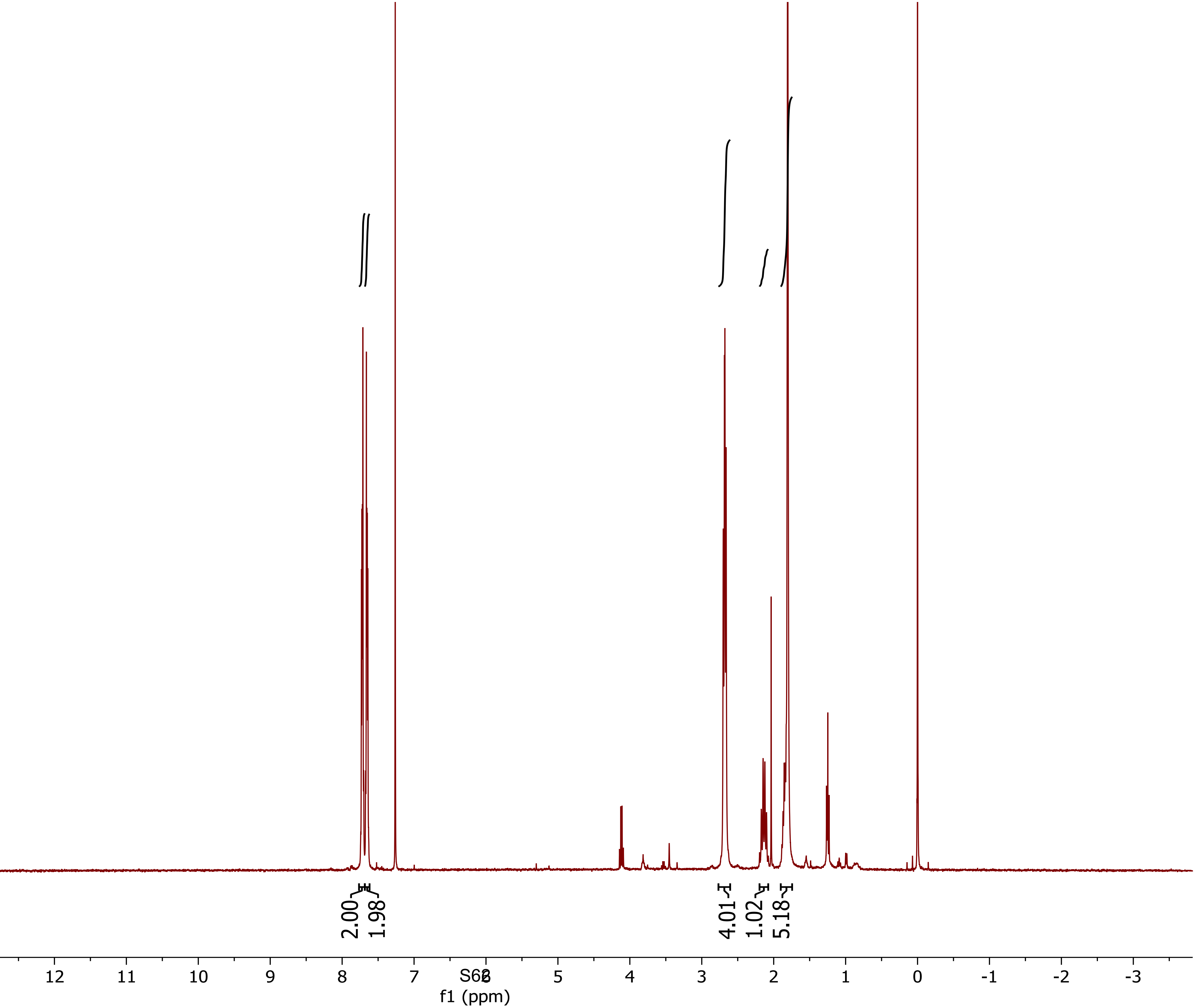
2.00
1.98
4.01
1.02
5.18
16 15 14 13 12 11 10 9 8 7 6 5 4 3 2 1 0 -1 -2 -3
f1 (ppm)

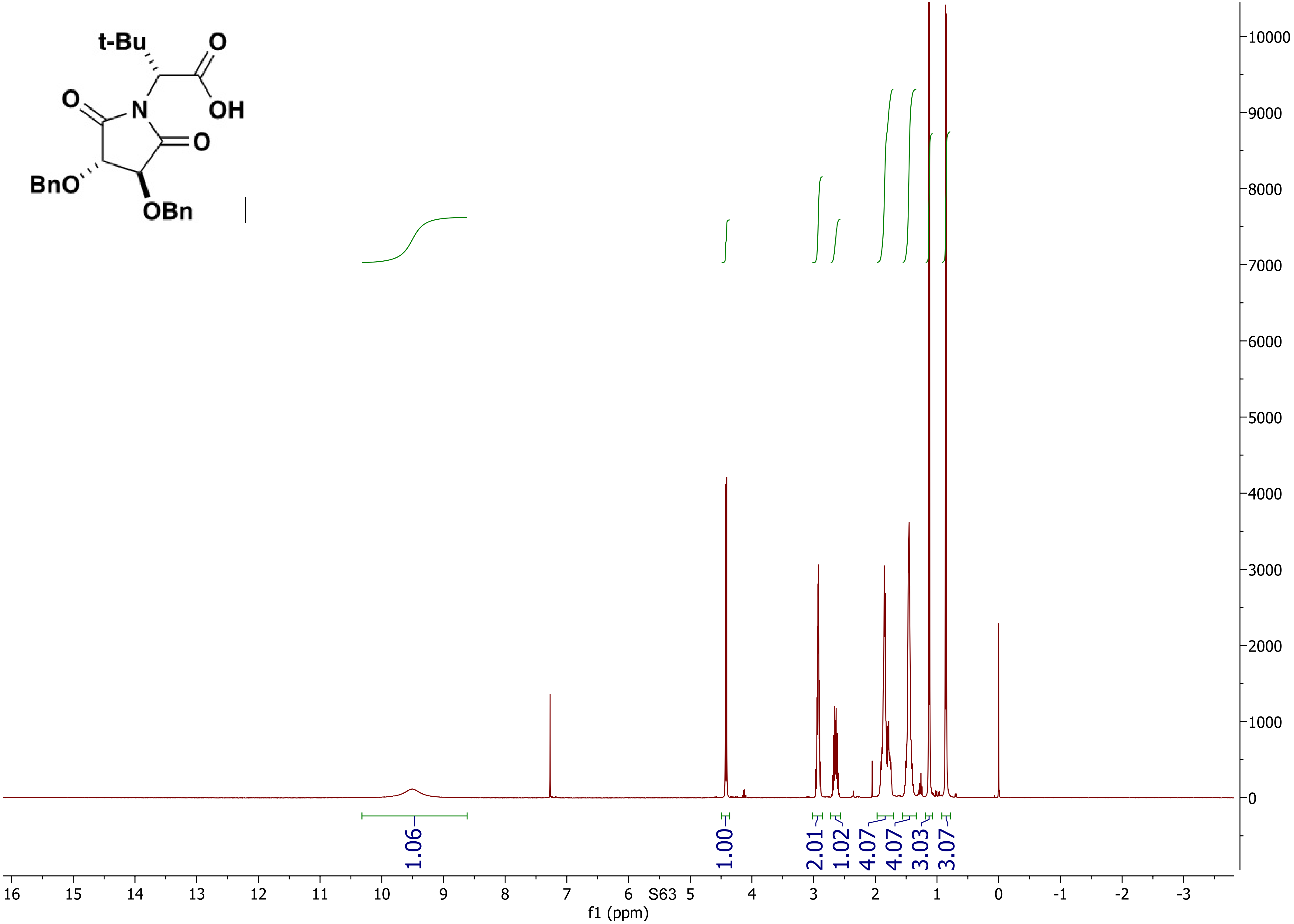
t-Bu
O
OH
O
N
O
BnO
OBn
1.06
1.00
2.01
1.02
4.07
4.07
3.03
3.07
16
15
14
13
12
11
10
9
8
7
6
5
4
3
2
1
0
-1
-2
-3
f1 (ppm)
10000
9000
8000
7000
6000
5000
4000
3000
2000
1000
0

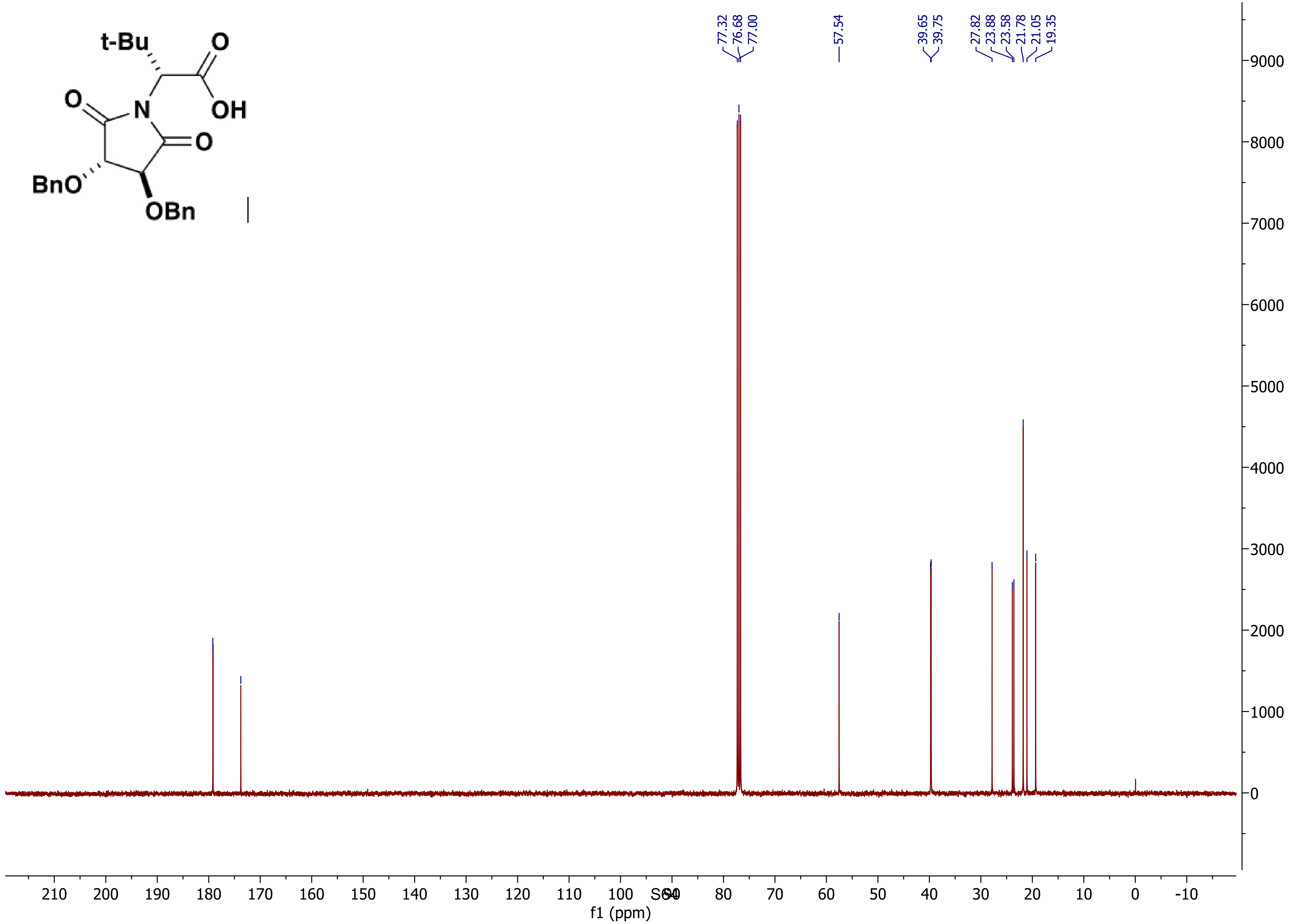
77.32
76.68
77.00
57.54
39.65
39.75
27.82
23.88
23.58
21.78
21.05
19.35
t-Bu
O
OH
O
N
O
BnO
OBn
f1 (ppm)
210 200 190 180 170 160 150 140 130 120 110 100 90 80 70 60 50 40 30 20 10 0 -10
9000 8000 7000 6000 5000 4000 3000 2000 1000 0

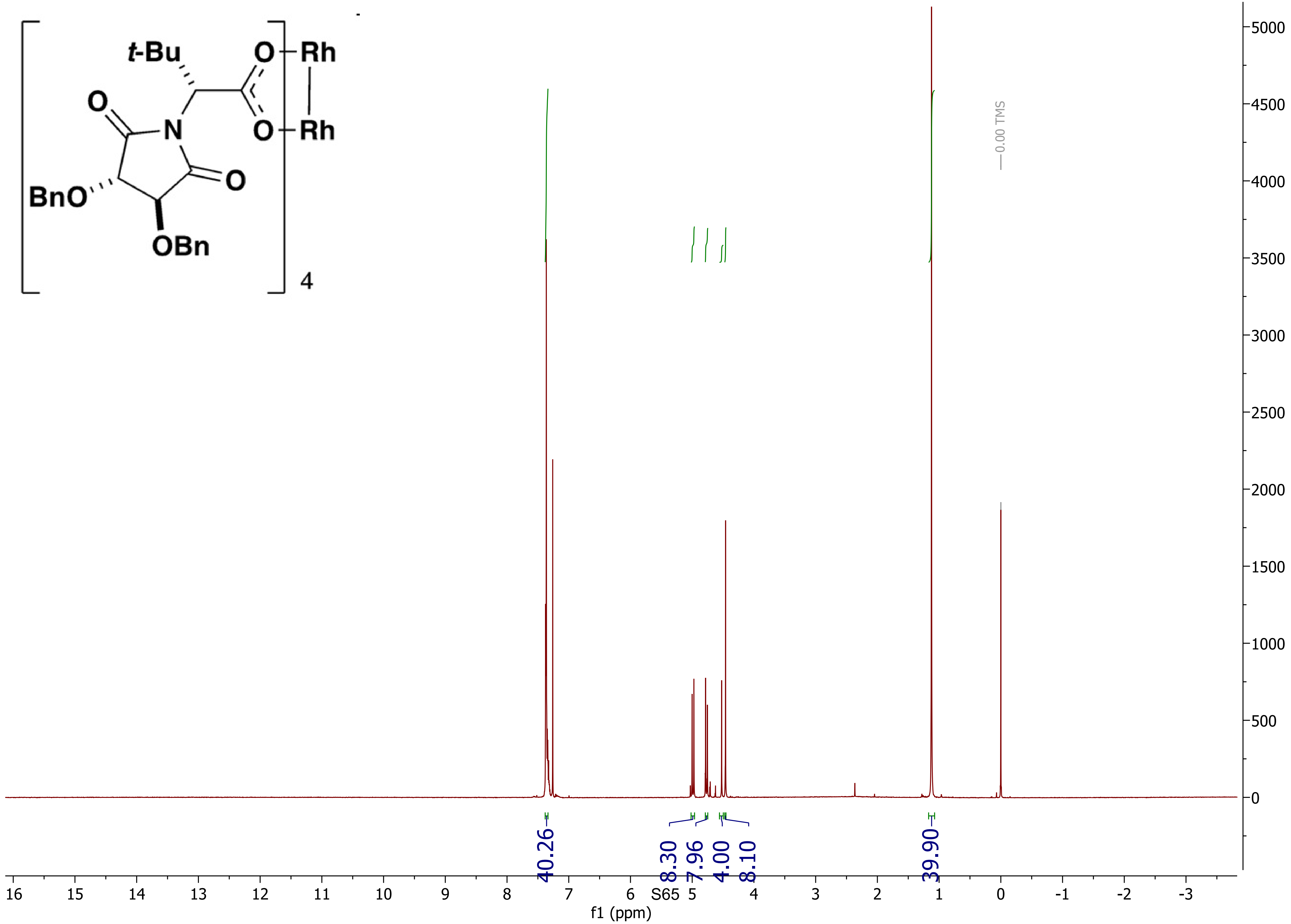
t-Bu
O–Rh
O–Rh
O
N
O
BnO
OBn
4
0.00 TMS
40.26
8.30
7.96
4.00
8.10
39.90
f1 (ppm)

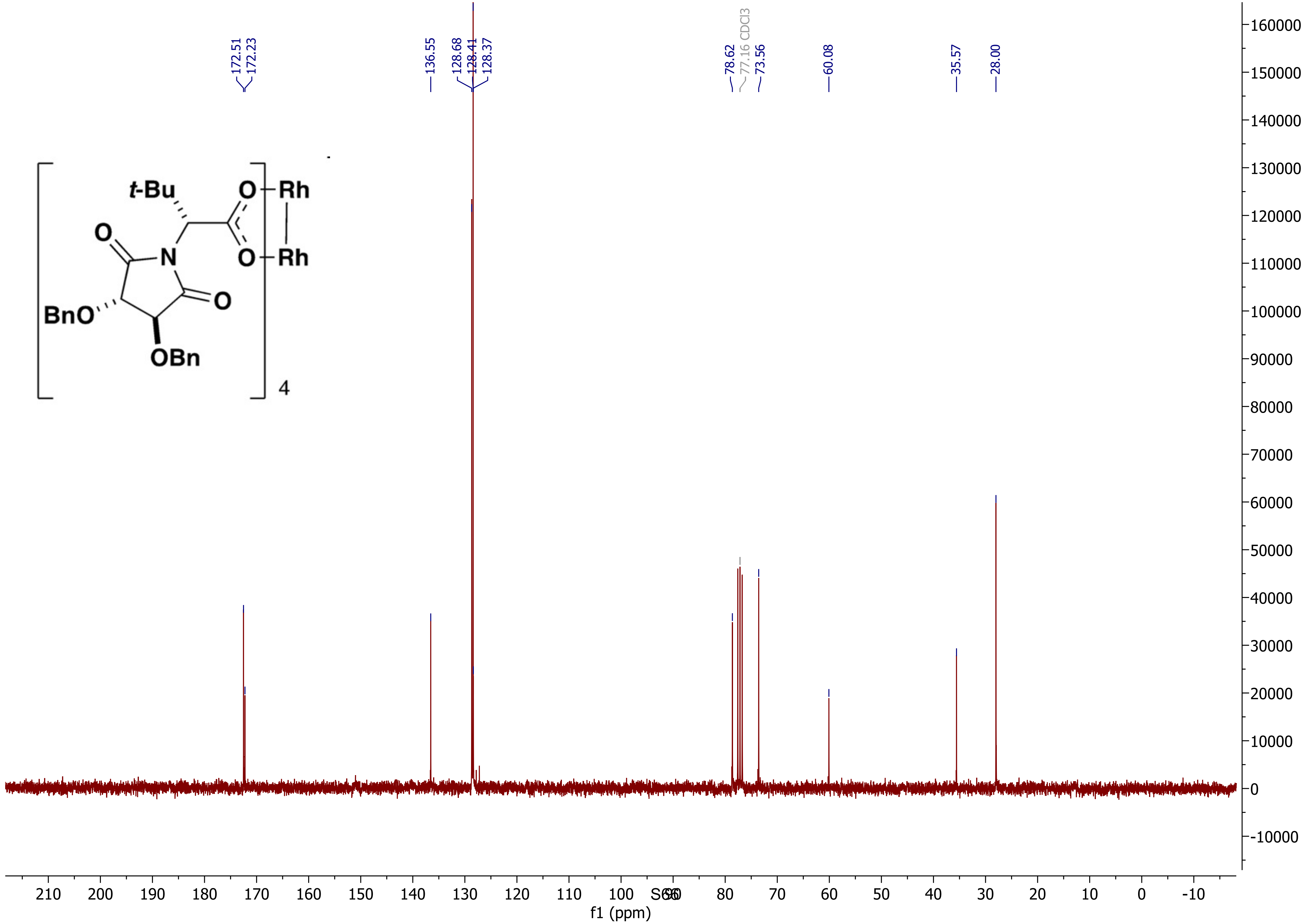

172.51
172.23
136.55
128.68
128.41
128.37
78.62
77.16 CDCl3
73.56
60.08
35.57
28.00
t-Bu
O
N
O
O
O
Rh
Rh
BnO
OBn
4
f1 (ppm)
210 200 190 180 170 160 150 140 130 120 110 100 90 80 70 60 50 40 30 20 10 0 -10
160000 150000 140000 130000 120000 110000 100000 90000 80000 70000 60000 50000 40000 30000 20000 10000 0 -10000
